# Shifted PAMs generate DNA overhangs and enhance SpCas9 post-catalytic complex dissociation

**DOI:** 10.1038/s41594-023-01104-6

**Published:** 2023-10-12

**Authors:** Jinglong Wang, Julien Le Gall, Richard L. Frock, Terence R. Strick

**Affiliations:** 1 Institut Jacques Monod, Université de Paris Cité, Paris, France; 2 Institut de Biologie, Ecole Normale Supérieure, Université PSL, Paris, France; 3 Department of Radiation Oncology, School of Medicine, Stanford University, Stanford, CA USA; 4 Programme « Equipe Labélisée » de la Ligue Nationale Contre le Cancer, Paris, France; 5 Present Address: School of Medicine, Stanford University, Stanford, CA USA

**Keywords:** Single-molecule biophysics, Enzyme mechanisms, Biophysical methods, DNA restriction-modification enzymes

## Abstract

Using Sanger sequencing and high-throughput genome sequencing of DNA cleavage reactions, we find that the *Streptococcus pyogenes* SpCas9 complex responds to internal mechanical strain by robustly generating a distribution of overhanging, rather than blunt, DNA ends. Internal mechanical strain is generated by shifting (increasing or decreasing) the spacing between the RNA-DNA hybrid and the downstream canonical PAM. Up to 2-base 3′ overhangs can be robustly generated via a 2-base increase in the distance between hybrid and PAM. We also use single-molecule experiments to reconstruct the full course of the CRISPR–SpCas9 reaction in real-time, structurally and kinetically monitoring and quantifying R-loop formation, the first and second DNA-incision events, and dissociation of the post-catalytic complex. Complex dissociation and release of broken DNA ends is a rate-limiting step of the reaction, and shifted SpCas9 is sufficiently destabilized so as to rapidly dissociate after formation of broken DNA ends.

## Abstract (web summary)

Here, using single-molecule experiments, the authors show that SpCas9 responds to shifted PAMs by inducing overhanging DNA ends. Such shift-PAM targeting is enhanced by physiological levels of DNA supercoiling and in turn promotes dissociation of the complex after catalysis.

## Main

CRISPR is a bacterial and archaeal adaptive immune mechanism that mobilizes Cas protein and guide RNAs to interact, recognize and cleave invasive DNA^1–4^. SpCas9, a well-characterized member of the type II-A CRISPR–Cas family, has been widely used to edit the genomes of prokaryotes and eukaryotes^5–7^. SpCas9 is a single protein of about 160 kDa with divalent ion-dependent HNH and RuvC endonuclease domains. SpCas9 can bind dual guide RNAs (dgRNAs), which are composed of the CRISPR RNA (crRNA) and the *trans*-activating CRISPR RNA (tracrRNA). crRNA and tracrRNA pair to form a ribonucleoprotein (RNP). tracrRNA is composed of 86 nucleotides (nt) and facilitates the maturation of the ~40-nt crRNA^8^. The 5′ region of tracrRNA and 3′ region of crRNA pair via complementary bases to form dgRNA, and in practice, the tracrRNA and crRNA can be engineered to form single guide RNA (sgRNA)^5^. The ~20-nt spacer of the guide RNA displaces the non-complementary DNA strand, and its hybridization with the protospacer of the complementary DNA strand results in formation of an R-loop. Cas9’s PAM-interaction domain (PID)^9^, which scans for NGG PAMs on the non-complementary strand, has also been found to be crucial for R-loop initiation and extension^10–12^. During R-loop formation, the targeted DNA and dgRNA–SpCas9 undergo dramatic and ordered conformational changes^11,13^. The HNH endonuclease domain aligns with the R-loop at the beginning of pre-catalytic complex formation between DNA and dgRNA–SpCas9, whereas the RuvC endonuclease domain becomes positioned on the non-complementary strand facing the HNH site later in the maturation of the catalytic complex^14^.

Early structural and single-molecule studies have revealed the importance of the NGG PAM in regulating SpCas9 catalytic activity^10^, as well as poor tolerance of nucleotide mismatches between the bases of guide RNA that seed the R-loop^15^. However, the PID can be engineered to display a certain level of plasticity—NGAN, NGNG, NGCG, and NAAG PAM variants have been characterized in SpCas9 mutants^16,17^, and off-target double-strand breaks (DSBs) formed by wild-type SpCas9 correlate to PAM variants such as GAG or AAG^18,19^.

Here, we find that SpCas9 responds to offsets of several nucleotides in the spacing between the crRNA-DNA hybrid and PAM by generating overhanging DSBs, and that this ‘shift-PAM’ targeting is enhanced by physiological levels of DNA supercoiling. We reach this conclusion by examining DNA end structures formed in ensemble biochemical and cell assays and by using single-molecule experiments to interrogate the SpCas9 catalytic mechanism: R-loop formation, first and second DNA-incision events, and dissociation of the post-cleavage complex from DNA.

## Results

### Shift-PAM-targeted SpCas9 robustly cleaves linear and supercoiled DNA

**Fig. 1:**
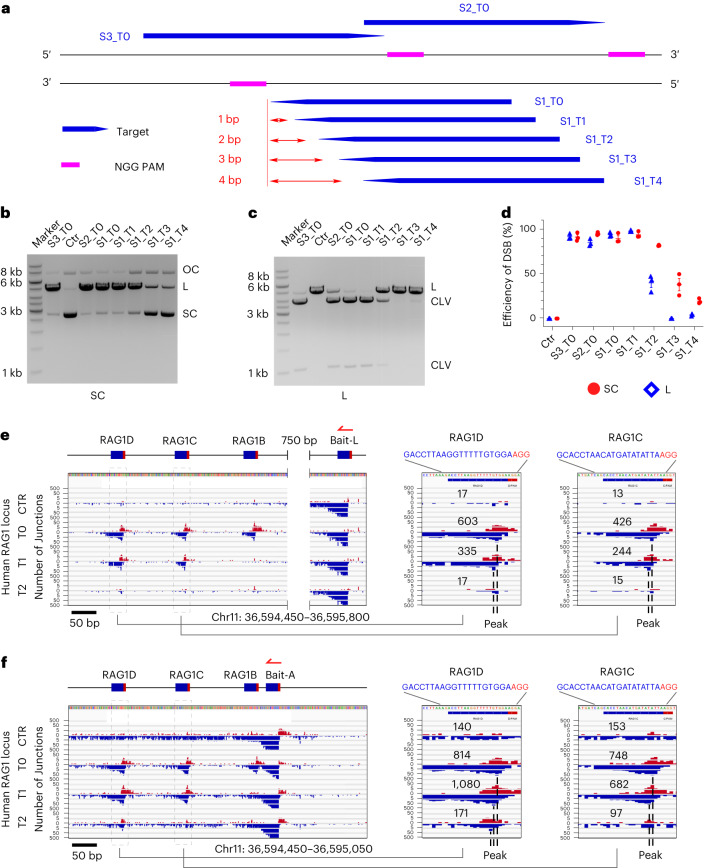
SpCas9 cleavage obtained by varying the spacing between the hybridized target and PAM. **a**, DNA loci targeted by RNPs with canonical (S1_T0 SpCas9, S2_T0 SpCas9, S3_T0 SpCas9) or shift-PAM spacing (S1_T1 SpCas9, S1_T2 SpCas9, S1_T3 SpCas9, S1_T4 SpCas9) between the hybridized target and PAM. Blue arrows represent the 20-nt targeting region for each RNP, and magenta rectangles represent the NGG PAM. **b**, RNPs with canonical or shift-PAM spacing process targeted supercoiled DNA (SC) first into nicked open circular DNA (OC) and then into linear DNA (L). **c**, As in **b**, but the linear DNA substrate (L) is processed into two cleaved products (CLV). **d**, The normalized efficiencies of quantified cleavage of circular DNA (red dot) and linear DNA (blue diamond), with error bars (s.e.m., *n* = 3). **e**,**f**, LAM-HTGTS IGV plots in the RAG1 locus of HEK293T cells using two different T0-PAM guides as bait (red harpoon), bait-L (**e**) and bait-A (**f**), to obtain the DSB profiles at the sites RAG1B, RAG1C, and RAG1D, generated by CTR (bait alone) or T0, T1 or T2 guides, respectively. Enlarged figures of RAG1D and RAG1C targets (dashed boxes on left) with the numbers of junctions are displayed in the right panels. Dashed lines indicate the peaks for the T0, T1, and T2 targets. Plots are displayed in a customized 0, 20, 200 scale for plus (red) and minus (blue) chromosome orientations. Supplementary Table 1 contains total number of junctions in the library and the data from the replicates of each bait. Source data.

**Extended Data Fig. 1:**
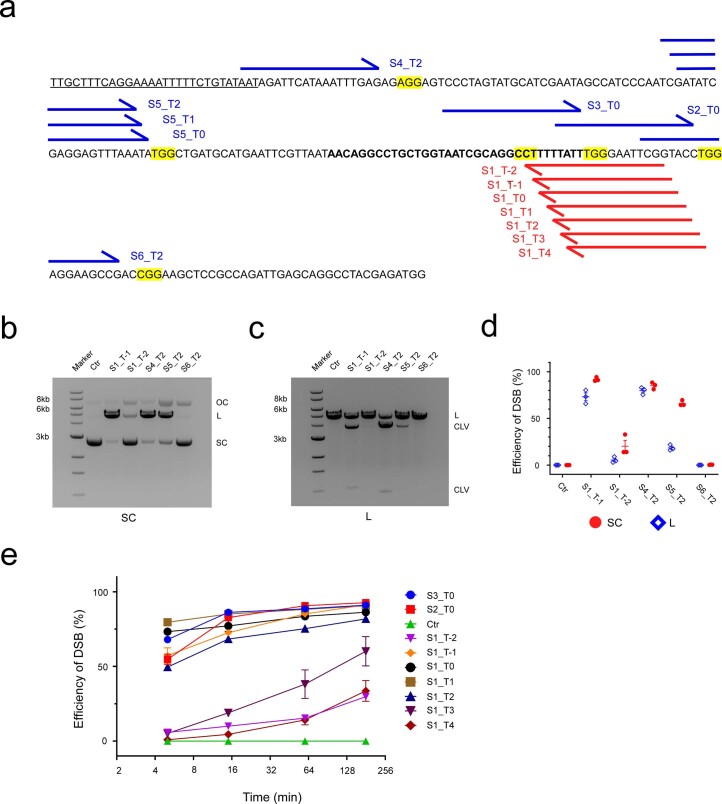
Cleaving DNA substrates using SpCas9-coupled guides that recognize canonical and shifted PAMs. (**a**) Guide RNAs were designed to target a bacterial transcript (shown 5’-3’) that contains an N25 promoter (underlined), a transcribed region and a tR2 terminator (bold). The 5’→3’ blue and red arrows represent the 20 bp protospacers located on the template and non-template strands of the bacterial transcript, respectively. The corresponding PAMs are highlighted in yellow. (**b-d**) S1_T1, S1_T2, S4_T2, S5_T2, and S6_T2 SpCas9 RNPs that generate (**b**) open circular (OC) and linear (L) DNA on supercoiled (SC) DNA substrates, and (**c**) two cleaved (CLV) fragments on linearized DNA substrates. (**d**) Efficiencies of DSB formation on supercoiled circular (SC) and linear (L) DNA are quantified and normalized (independent experiments, n = 3). (**e**) Ensemble assay of SpCas9 with various guides on supercoiled DNA substrates. Cleaved samples were taken at 5, 15, 60 and 180 min. time-points, loaded on an agarose gel and quantified by ImageJ. Means and corresponding SEMs are shown in the plot (independent experiments, n = 2). Source data

We used canonical and shift-PAM guide RNAs to induce cleavage within a synthetic gene^20^. We first designed a canonical crRNA targeting a 20-nt protospacer with a consensus NGG PAM located on the coding strand of the synthetic gene (S1_T0 crRNA). We then designed 20-nt shift-PAM crRNAs targeting DNA in base-pair increments upstream of the canonical S1_T0 crRNA target (S1_T1, S1_T2, S1_T3, and S1_T4 crRNAs). To control for cleavage, we examined two canonically targeted SpCas9 sites on the non-coding strand (S2_T0 and S3_T0) (Fig. 1a and Extended Data Fig. 1a). The various crRNAs were hybridized with conserved tracrRNAs to form dgRNAs and were subsequently combined with SpCas9 to form enzymatic dgRNA–SpCas9 RNP complexes. To assess DNA-strand cleavage activity in the presence or absence of supercoiling, the differentially targeted dgRNA-SpCas9s were added in ensemble assays to either supercoiled or SbfI-linearized ~5-kb double-stranded DNA substrates and then were assessed for single- or double-strand cleavage using gel electrophoresis (see Methods).

As predicted, canonically targeted RNPs (S1_T0, S2_T0, and S3_T0) efficiently cleaved both DNA strands of supercoiled and linear DNA substrates within 3 h (Fig. 1b–d). Unexpectedly, shift-PAM RNPs (S1_T1 and S1_T2) generated robust levels of DSBs for supercoiled substrates, comparable to levels observed on canonically targeted controls (Fig. 1b,d). Double-strand cleavage efficiency progressively decreased with increasing shift-PAM spacing, with an increasing ratio of incised (nicked) versus linearized fragments from S1_T3 and S1_T4 RNPs (Fig. 1b,d), reflecting an accumulation of partially processed intermediates. In contrast to supercoiled substrates, the decline in cleavage efficiency of the linearized substrate was much more pronounced: S1_T1 SpCas9 DSB cleavage of linear DNA was indistinguishable from that of supercoiled DNA, whereas S1_T2 SpCas9 DSB cleavage was substantially reduced and very little cleavage was detected for S1_T3 and S1_T4 RNPs (Fig. 1c,d). Overall, the increased spacing between the PAM and the RNA-targeted sequences was generally more tolerated on supercoiled DNA than on linearized DNA, in a manner reminiscent of the way in which mutations in the spacer are better tolerated on negatively supercoiled DNA^21^.

To get a better sense of how shift-PAM cleavage varies across sequence contexts, we targeted supercoiled and linear DNA at three additional sites using crRNAs implementing 2-base-pair (bp) spacing (S4_T2, S5_T2, S6_T2) (Extended Data Fig. 1a). S5_T2 SpCas9 complexes acted robustly on the supercoiled DNA substrate, but much less so on the linear DNA substrate, as was observed with S1_T2 SpCas9. The S4_T2 and S6_T2 RNPs displayed extreme opposite activities, with the former robustly generating DSBs in both supercoiled and linear contexts, and the latter nicking supercoiled DNA only slightly (Extended Data Fig. 1b–d). We also investigated whether SpCas9 cleaved DNA strands when the 20-nt target sequence was moved closer to the PAM in base-pair increments. Thus, we also generated S1_T-1 and S1_T-2 crRNAs (Extended Data Fig. 1a) and assayed them in the ensemble assays described above. For supercoiled substrates, we found robust DSB generation with S1_T-1 SpCas9 and low DSB generation with S1_T-2 SpCas9, and both of these activities were attenuated in the linear-substrate ensemble (Extended Data Fig. 1b–d). Collectively, our results suggest that SpCas9 is capable of cleaving both DNA strands when the targeted sequence and the PAM are offset in either direction by up to 2 bp when the DNA substrate is supercoiled.

A time-course of supercoiled DNA cleavage showed that canonically targeted RNPs (S1_T0, S2_T0, and S3_T0 SpCas9) rapidly reached DSB saturation on the timescale of several minutes. In general, the kinetics of DSB formation in shift-PAM-targeted RNPs with a mild shift (S1_T1, S1_T2, and S1_T-1, but not S1_T-2, SpCas9) were similar to those in canonically targeted RNPs, whereas shift-PAM RNPs with larger shifts (S1_T3 and S1_T4, SpCas9) slowly accumulated DSBs over 3 h (Extended Data Fig. 1e).

### The DSB-generating activity of shift-PAM-targeted RNPs depends on the canonical PAM

**Extended Data Fig. 2:**
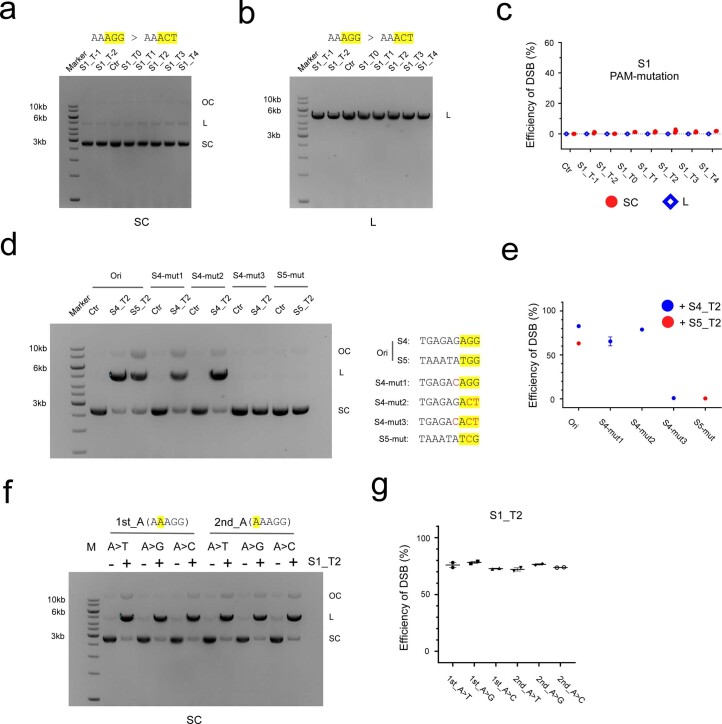
Different mutations were introduced into PAM or PAM-adjacent sites to assess the impact on SpCas9 cleavage activity using various guides. (**a-c**) The AGG PAM of the S1 locus was mutated to ACC and supercoiled DNA (‘SC’, a) or linearized DNA (‘L’, b) bearing this mutation was cleaved by S1_T1, S1_T2, S1_T0, S1_T1, S1_T2, S1_T3 and S1_T4 SpCas9, respectively. Cleavage efficiencies were quantified, normalized, and plotted (c), with error bar (SEM, independent experiments n = 3). (**d-e**) Three mutants for the S4 locus were generated: S4-mut1 (PAM-adjacent guanine mutation), S4-mut2 (PAM mutation), and S4-mut3 (double mutation). One mutant for the S5 locus was generated, S5-mut. These mutants together with the original DNA (Ori) were cleaved by S4_T2 or S5_T2 SpCas9 (d), and the efficiency of DSB was calculated and normalized by subtracting the controls and shown in percentage with SEM (e, independent experiments n = 3). (**f-g**) The first and second adenines adjacent to the canonical PAM of the S1 locus were mutated to T, G, C respectively. The supercoiled forms of the corresponding mutants were cleaved by S1_T2 SpCas9 (f), and the efficiencies were calculated and shown in the right panel with SEM (g, independent experiments n = 2). Source data

We reasoned that if the shift-PAM mechanism relies on the nearby canonical PAM, mutation of the NGG PAM would eliminate the DSB cleavage activity of shift-PAM-targeted RNPs. Indeed, mutating both guanines of the PAM in the S1 locus abolished the DSB-generating activity of all canonically and shift-PAM-targeted RNPs in both supercoiled and linearized DNA substrates (Extended Data Fig. 2a–c). While the same result was achieved for S5_T2, S4_T2 required additional mutations to remove alternative PAMs^16^ to abolish the DSB cleavage completely (Extended Data Fig. 2d,e). We next addressed whether changes within the intervening 2-nt offset sequence resulting from use of the S1_T2 crRNA influenced DNA processing. Mutating the first and second adenines adjacent to the canonical PAM to T, G, or C did not affect DSB cleavage by S1_T2 SpCas9 (Extended Data Fig. 2f,g). Thus, shift-PAM targeting strictly requires the contribution of the canonical PAM and places additional strain on the non-complementary strand.

### Plasticity of endonuclease activities for shift-PAM-targeted complexes

**Extended Data Fig. 3:**
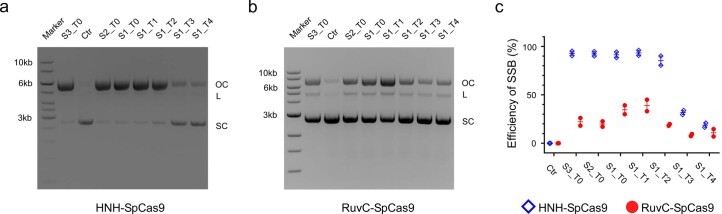
Supercoiled DNA with canonical and spaced PAMs were nicked using single-nuclease SpCas9 mutants. (**a**) HNH-SpCas9 (D10A) nickase (containing an active HNH domain but inactive RuvC domain) was assembled with various dgRNAs into RNPs to incise the corresponding target double-stranded circular DNA containing either a canonical PAM or a shifted PAM. (**b**) As with the prior panel but using RuvC-SpCas9 (H840A) nickase (containing an active RuvC domain but inactive HNH domain). (**c**) Efficiencies of SSB formation are quantified, normalized and shown as blue diamonds for HNH-SpCas9(D10A) and red dots for RuvC-SpCas9(H840A). Error bars indicate SEM (independent experiments, n = 2). Source data

Single-strand breaks accumulated in supercoiled DNA substrates with 3–5-bp PAM spacing, suggesting that either the HNH or RuvC domain could be solely responsible for generating such breaks. To test this, we employed two widely used nickases, SpCas9-D10A, which possesses a catalytically active HNH domain but a dead RuvC domain (denoted HNH-SpCas9), and SpCas9-H840A, which possesses a catalytically dead HNH domain but an active RuvC domain (denoted RuvC–SpCas9). The ability of HNH-SpCas9 to robustly nick substrate was unaffected as spacing initially increased (S1_T0, S1_T1, S1_T2), but this activity dropped by roughly threefold at S1_T3 spacing and decayed to nearly zero with S1_T4 spacing (Extended Data Fig. 3a–c). Spacing-dependent incision by RuvC–SpCas9 displayed a slightly more complex pattern, with an increase in incision from S1_T0 to S1_T1 spacing but a progressive decay as spacing increased to S1_T4, at which incision nevertheless remained at ~60% of the level observed for S1_T0 (Extended Data Fig. 3a–c). Overall, we find that incision of the supercoiled substrate by the HNH domain is robust up to S1_T2 spacing but slows beyond that, whereas incision by the RuvC domain is more tolerant of increased spacing over the same scale. Comparing the pattern of incision for increased spacing observed in the two mutants with that observed in wild-type SpCas9 (Fig. 1b), we note that incision of supercoiled substrate by the wild-type enzyme also dropped significantly, by threefold, upon increasing from S1_T2 to S1_T3, pointing to a dominant role for HNH incision in the response of DNA processing to increased spacing and its enhancement by DNA supercoiling (Fig. 1d and Extended Data Fig. 1a,c).

### Shift-PAM-targeted SpCas9 generate DSBs in living cells

**Extended Data Fig. 4:**
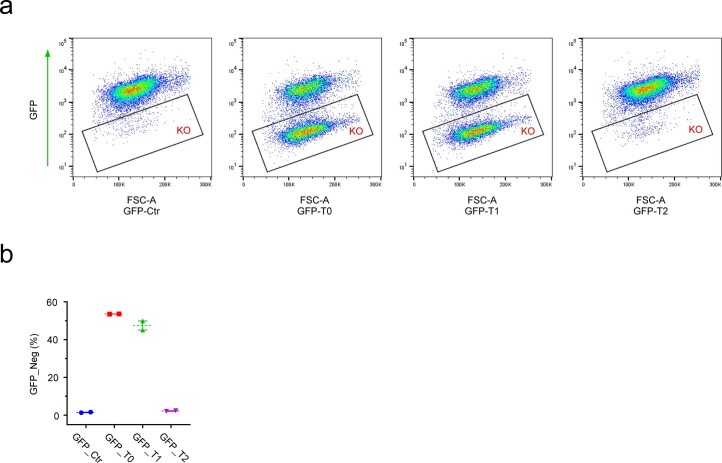
Canonical and PAM-shifted RNPs applied to gene knockout. (**a**) Flow cytometry measurement of GFP in K562-iCas9-GFP cells nucleofected by pMCB320 bearing sgRNA genes of GFP-Ctr, GFP_T0, GFP_T1 and GFP_T2, respectively. (**b**) The percentages of GFP-negative cells, GFP_Neg (‘KO’ in **a**) are plotted with SEM (independent experiments, n = 2).

**Extended Data Fig. 5:**
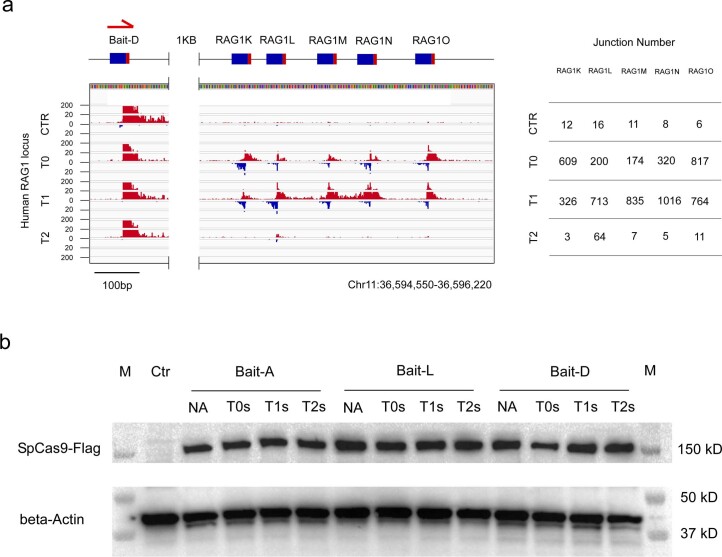
Comparing DSBs obtained using canonical and shifted-PAM guides at five additional sites. (**a**) Bait-D (harpoon) targets a locus > 1 kb upstream of the five prey sites RAG1K-O, and was delivered alone as negative control (CTR) or together with canonical PAM (T0) and shifted-PAM (T1, T2) sgRNAs into HEK293T cells, respectively. IGV-plot shows both T0 and T1 can generate decent amounts of cleavage at all five sites, while cleavage generated by T2 was barely detected except with the RAG1L_T2 guide. The number of junctions for each cluster is provided in the right panel. (**b**) Expression levels of flag-tagged SpCas9 (~150 kD) for all conditions including Bait-A, Bait-L and Bait-D series were determined by probing with an anti-flag antibody, and beta-actin (~40 kD) was used as a loading control. Source data

To determine whether spaced SpCas9 cleavage can be detected in human cells, we first used it to knock out GFP in K562-iCas9-GFP cells. First we compared the knock-out efficiency of canonical (GFP_T0) with that of shift-PAM (GFP_T1, GFP_T2) guides, using a generic canonical target in the RAG1 locus (RAG1L_T0) as a negative control. GFP_T1 sustained slightly weaker knock-out efficiency (~45%) than GFP_T0 (~55%), while GFP_T2 generated marginal knock-out efficiency (Extended Data Fig. 4a,b). Second, we used high-throughput genome-wide translocation sequencing (linear amplification mediated high-throughput genomic translocations sequencing (LAM-HTGTS))^22^ in HEK293T cells to compare translocations to three canonically targeted (T0) SpCas9 prey DSBs in the RAG1 locus (RAG1B, RAG1C, and RAG1D)^18^ and their shift-PAM-targeted variants (T1, T2) using two independent and canonically targeted bait DSBs (bait-A and bait-L) located within 1 kb of the SpCas9 prey DSBs (Fig. 1e,f). From both baits, we recovered several hundreds of translocations from RAG1C_T1 and RAG1D_T1, but not RAG1B_T1, prey sites that were at a similar or modestly reduced level compared with translocations recovered from RAG1C_T0 and RAG1D_T0 prey sites (Fig. 1e,f and Supplementary Table 1). Notably, the translocation peaks for the complementary-strand breakpoints observed with the T1-targeted SpCas9 prey DSBs were shifted by 1 bp relative to those observed with T0 spacing (Fig. 1e,f). At this series of sites we observed no translocations from RAG1B_T2 and RAG1C_T2 but did observe translocations from RAG1D_T2, and the corresponding translocation peaks on the complementary strand displayed a corresponding 2-bp shift (Fig. 1f). Five additional canonically and shift-PAM-targeted sites (RAG1K–RAG1O), using RAG1D_T0 as bait (Bait-D), displayed similar T0 versus T1 profiles, with only RAG1L_T2 forming substantial translocations (Extended Data Fig. 5a and Supplementary Table 1). Bait–prey LAM-HTGTS experiments generally expressed comparable levels of SpCas9 protein across all conditions (Extended Data Fig. 5b). Therefore, T1 and T2 shift-PAM targeted DSBs can form in cells at levels lower than their canonically-targeted counterparts, yet robustly enough to be detected by translocation.

### Shift-PAM-targeted SpCas9 cleavage leads to 3′ overhangs

**Extended Data Fig. 6:**
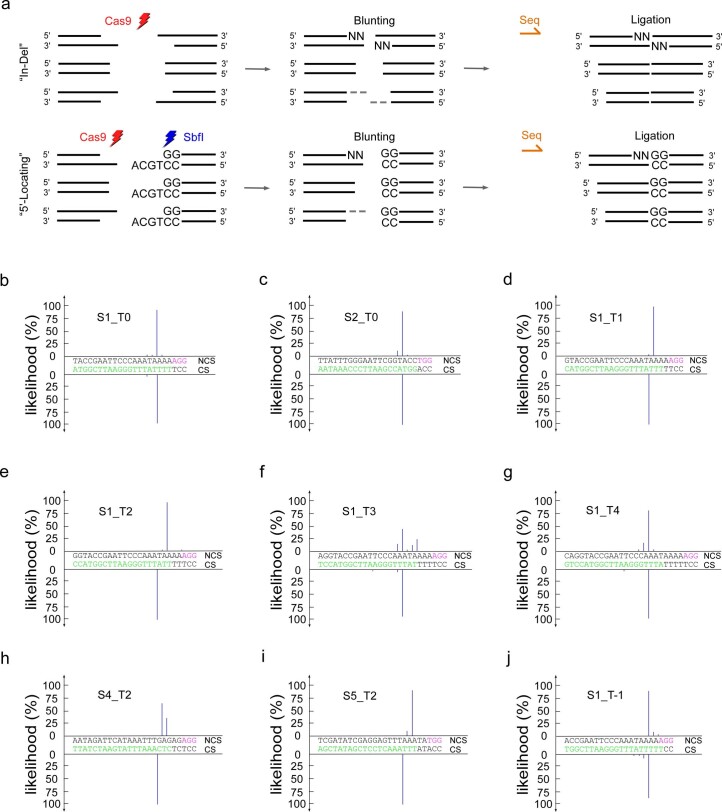
Identifying cleavage sites by ‘In-Del’ and ‘5’-locating’ approaches. (**a**) Sketch of ‘In-Del’ and ‘5’-locating’ end-mapping methods, in which supercoiled DNA is processed by either Cas9 or by Cas9 followed by Sbf-I digestion and blunting (either by 5’→3’ nucleotide addition at 3’-recessed ends or 3’→5’ nucleotide deletion at 3’-overhang ends. DNA is then ligated and Sanger-sequenced (Seq; see Methods for details). (**b-j**) Resulting likelihood plots of cleavage sites for (b) S1_T0, (c) S2_T0, (d) S1_T1, (e) S1_T2, (f) S1_T3, (g) S1_T4, (h) S4_T2, (i) S5_T2 and (j) S1_T-1 SpCas9. The height of the blue bar represents the likelihood of incision at the position of the DNA strand indicated. Green and magenta letters denote the protospacers and nearby canonical PAMs, respectively. At least 30 samples were sequenced for each condition (See Supplementary Table 1).

SpCas9 generates DSBs described predominantly as blunt-ended^5,23^, but can also generate DSBs with short 5′ overhangs in cells^24,25^ that are likely due to increasing variance in cleavage position on the non-complementary strand over time^5^. However, it is unclear whether the cleaved products using shift-PAM spaced sites would predominantly bear blunt ends or overhangs. To avoid the extensive end processing present in cells, we used a combination of two Sanger sequencing approaches, In-Del and 5′-locating (Extended Data Fig. 6a), to obtain the likelihood of end structures from RNPs with shift-PAM guides in biochemistry assays (Extended Data Fig. 6b–j and Supplementary Table 2). The In-Del method is designed to capture the insertion, deletion or direct joining of each targeted DSB, and 5′-locating takes advantage of an upstream SbfI site to identify the 5′ ends generated by the SpCas9 RNP (Extended Data Fig. 6a).

Using these sequencing approaches, almost all incision sites on the complementary strand were identified at position 17 from the 5′ end of the 20-nt targeting sequence in all instances assayed (Extended Data Fig. 6b–j and Supplementary Table 2), faithfully positioned within the RNA-DNA hybrid even as the hybrid was shifted away from the PAM in base-pair increments. The incision site on the non-complementary strand also remained faithfully positioned with respect to the PAM site for up to 2-bp shifts (such as for S1_T0, S2_T0, S1_T1, S1_T2, and S5_T2), while more than half of the observed incision sites for S4_T2 shifted by 1 bp. However, S1_T3 RuvC–SpCas9 incision was promiscuously distributed across the 3-bp spacer region and was also in alignment with the HNH cleavage site, whereas S1_T4 RuvC strand cleavage was nearly entirely in alignment with the HNH cleavage position (Extended Data Fig. 6b–i and Supplementary Table 2). As a result, whereas S1_T0 and S2_T0 targets had blunt ends, the majority of S1_T1, S1_T2, S4_T2, and S5_T2 targets had 1–2-nt 3′ overhangs. S1_T3 generated a mix of overhangs and blunt ends, and S1_T4 generated blunt ends. This is reminiscent of earlier research demonstrating shift-PAM-mediated cleavage by *Streptococcus thermophilus* LMG18311 (LMG18311 Cas9) in a biochemical assay^26^.

We thus also investigated whether shifting the RNA-DNA hybrid towards the PAM produced 5′ overhangs; however, S1_T-1 targeting resulted nearly entirely in blunt ends that were 2 bp away from the PAM (Extended Data Fig. 6j), indicating steric hindrance by HNH targeting. The altered positional cleavage of the non-template strand in the contexts of S1_T3, S1_T4, and S1_T-1 shows that RuvC endonuclease activity does not require consensus spacing to the NGG sequence recognized by the PID for incision. These combined observations suggest that shift-PAM-targeted SpCas9 cleavage relies on a DNA-mediated induced fit mechanism.

### SpCas9 RNP dynamics on single supercoiled DNA molecules

**Fig. 2:**
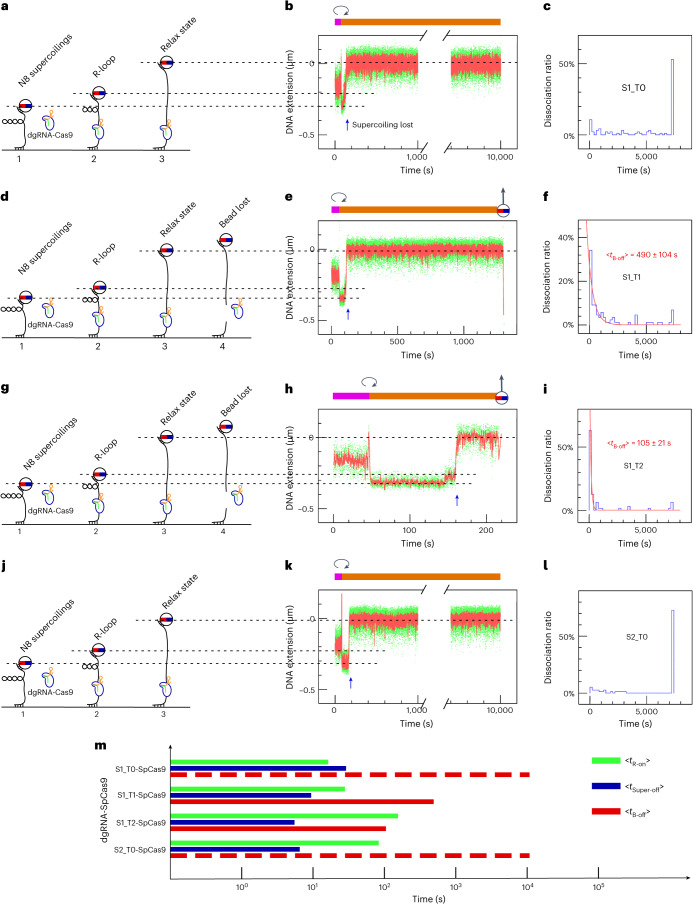
Real-time observation of pre-catalytic and post-catalytic steps by SpCas9 coupled with canonical and shift-PAM guides. **a**–**c**, Experiment carried out using S1_T0 SpCas9 RNPs. **a**, Sketch of the assay. SpCas9 RNPs are introduced under non-permissive conditions (positive supercoiling), and (1) activity is initiated by generating eight negative supercoils in the DNA (see Methods and Extended Data Fig. 7). (2) R-loop formation is detected as an increase in DNA extension, and is followed by (3) loss of DNA supercoiling. **b**, Typical time-trace for the assay. The magenta bar highlights the phase during which DNA is positively supercoiled for the purposes of RNP injection; the clockwise arrow denotes the moment at which the DNA is negatively supercoiled to initiate the reaction, and the orange bar highlights the phase during which DNA is negatively supercoiled. The magnetic bead with a vertical arrow represents irreversible loss of the magnetic bead. Dash lines are guides highlighting the bead position in the different states, including initial negative supercoiling, the R-loop state, and the torsionally relaxed state (supercoiling lost, blue arrow). The time-trace y-axis is labelled such that the maximal bead position above the surface is set to zero, and lower extensions are reported as negative values. **c**, The fraction of molecules forming an R-loop that go on to display terminal bead loss (monitored over 7,200 s). The subsequent rows are structured in the same way as the initial row containing **a**–**c**. In **d**–**f**, S1_T1 SpCas9 was used and as a result we now frequently observe irreversible loss of the magnetic bead (depicted in panel **d** as step 4 and indicated in panel **e** as the magnetic bead topped by a vertical arrow); in **g**–**i**, S1_T2 SpCas9 was used; and in **j**–**l**, S2_T0 SpCas9 was used. **m**, The timeline summary of events after initiating RNP activity is displayed in log scale: mean time required for R-loop formation (<*t*_R-on_>, green lines), mean time required for supercoil loss after R-loop formation has been observed (<*t*_Super-off_>, blue lines) and mean time required for irreversible loss of magnetic bead after loss of supercoiling has been observed (<*t*_B-off_>, red lines (quantified) and dotted red lines (estimated)). See Supplementary Table 3 for detailed values. Source data

**Extended Data Fig. 7:**
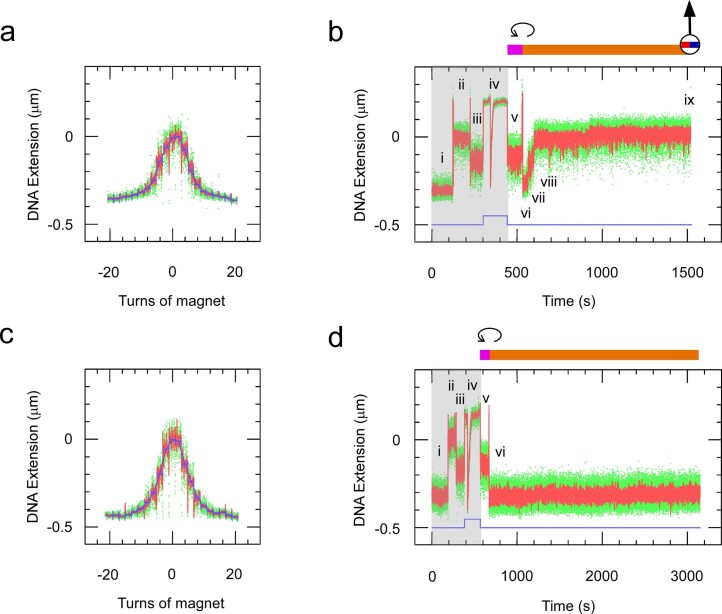
Procedure for DNA molecule calibration and dgRNA-SpCas9 injection using single-molecule magnetic trapping. (**a**) Typical extension vs. supercoiling curve showing a DNA molecule rotated from 20 turns of positive supercoiling to 20 turns of negative supercoiling under a constant ~0.2 pN extending force, allowing us to identify un-nicked dsDNA molecules and also to calibrate the change in DNA extension for a unit change in DNA writhe (Strick et al. 1996), here typically ~55 nm/turn. (**b**) Extension time-trace for the molecule calibrated in (a), providing real-time references at a constant 0.2 pN extending force (blue line) for its extension at (i) 8 turns of negative supercoiling, (ii) zero supercoiling (that is the torsionally-relaxed state), and (iii) 5 turns of positive supercoiling before (iv) increasing the applied force to 1 pN and injecting S1_T0 SpCas9 RNPs (seen as a downward spike in extension as flow pushes the bead towards the surface). Once flow ends and the bead returns to its extension prior to injection (v) the applied force is returned to 0.2 pN and the extension is verified to return to that see in (iii). After a few minutes (vi) DNA molecules are returned to 8 turns of negative supercoiling. (vii) R-loop formation and (viii) supercoil loss are followed by (ix) terminal loss of the magnetic bead. (**c**, **d**) as in (a,b) but with injection of only the S1_T0 guide (that is without SpCas9) shows that no R-loops are formed within 2000 seconds of detection window. This experiment was repeated on a total of 72 molecules.

To kinetically characterize the succession of DNA-processing steps by shift-PAM targeting RNPs and compare them with processing by canonically targeted RNPs, we carried out single-molecule assays based on the magnetic trap (Fig. 2a). Single DNA molecules containing target sequences and bearing biotin labels at one end and digoxigenin (dig) labels at the other are first attached to streptavidin-coated magnetic beads and then deposited on and attached to an anti-dig-coated glass slide^27^. The slide is placed under a microscope, and two magnets are used to apply a force on and rotate the magnetic bead. Imaging of the bead position above the surface allows measurement of DNA extension, and thus conformation, in real-time. For a given, low, fixed force (*F* = 0.2 pN for the measurements herein), the extension of torsionally relaxed DNA represents a maximum (Extended Data Fig. 7). Supercoiling the DNA causes it to compact, which is observed in the magnetic trap as a decrease in the ‘altitude’ of the bead above the surface. DNA incision results in an abrupt loss of supercoils, without which the DNA is no longer compact and the bead recovers its position in the torsionally relaxed state. DNA molecules that do not compact upon supercoiling during the initial calibration steps of the assay are pre-nicked and are excluded from analysis.

Because the torque from positive supercoiling inhibits R-loop formation, RNP injection took place on positively supercoiled DNA extended by 1 pN force. After RNP injection, the force was reduced to 0.2 pN, and eight negative supercoils were applied to the DNA to initiate single-molecule detection of SpCas9 RNP dynamics: R-loop formation (apparent as DNA unwinding^21^, as in Fig. 2b,e,h,k and Extended Data Fig. 7b,d(state vii)), single-strand DNA incision (apparent as a sudden loss of supercoiling^28^, blue arrows in Fig. 2b,e,h,k and Extended Data Fig. 7b,d(state viii)), and double-strand DNA breakage (apparent as terminal loss of the magnetic bead^29^, see Fig. 2e,h, and Extended Data Fig. 7b,d(state ix)). Using this assay, we could specify the waiting time required to form R-loops (<*t*_R-on_>, the time spent in in state vi), the time elapsed from R-loop formation to supercoil dissipation (<*t*_Super-off_>, the time spent in state vii), and the time elapsed from supercoil dissipation to terminal loss of the magnetic bead (<*t*_Bead-off_>, the time spent in state viii prior to ix, at which point in time the bead is lost).

### Unstable shift-PAM-targeted SpCas9 post-cleavage complexes

**Extended Data Fig. 8:**
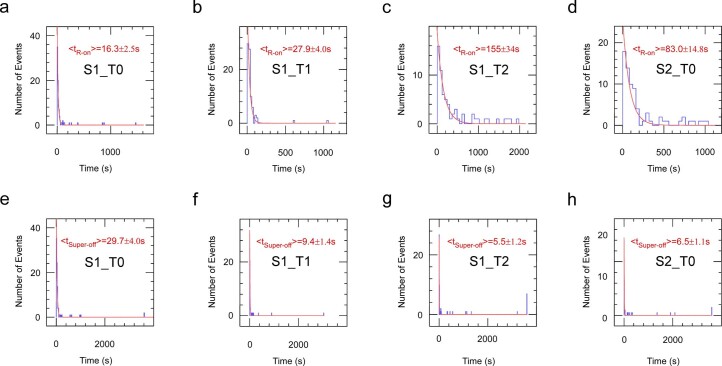
Characterization of the kinetics of R-loop formation and supercoiling loss using various dgRNA-SpCas9 RNPs. (**a-d**) Distributions of waiting times for R-loop formation obtained for four different RNPs (blue) are fitted to single-exponential decays (red) to obtain the corresponding average waiting time, <t_R-on_ > (± SEM), for (**a**) S1_T0 SpCas9, (**b**) S1_T1 SpCas9, (**c**) S1_T2 SpCas9 and (**d**) S2_T0 SpCas9. (**e-h**) Similarly, the mean time elapsed between R-loop formation and supercoil dissociation, <t_Super-off_>, was calculated for (**e**) S1_T0 SpCas9, (**f**) S1_T1 SpCas9, (**g**) S1_T2 SpCas9 and (**h**) S2_T0 SpCas9. See Supplementary Table 2 for further details. Source data

The first key observation from these measurements is that progressive increases in distance from S1_T0 to S1_T1 and then S1_T2 results in a progressive increase in <*t*_R-on_> and a progressive decrease in <*t*_Super-off_> (Fig. 2b,e,h,m, Extended Data Fig. 8a–c,e-g, and Supplementary Table 3). The increase in <*t*_R-on_> is consistent with the notion that increased mechanical stress in shift-PAM SpCas9–DNA complexes increases the energetic cost and hence time required to form the R-loop state. The decrease in <*t*_Super-off_> suggests that mechanically stressed complexes lead to faster dissipation of supercoils. This could result, somewhat counterintuitively, from faster kinetics for single-strand incision, or alternatively from a reduced ability of stressed complexes to ‘grasp’ the DNA on either side of the nick to prevent DNA from swiveling freely and dissipating supercoils.

**Extended Data Fig. 9:**
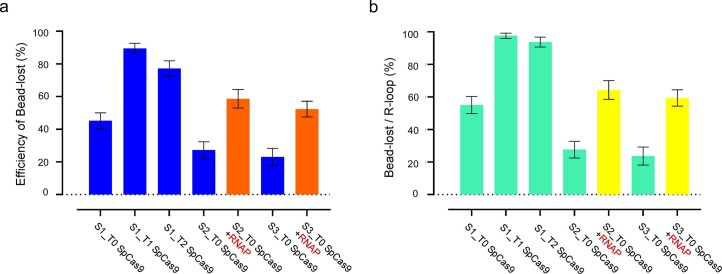
Apparent efficiency of DSB formation via magnetic trapping microscopy as determined by terminal loss of the magnetic bead within a three-hour window. (**a**) Raw percentages of all observed DNA molecules displaying terminal bead loss in assays including S1_T0 SpCas9 (45.2 ± 4.9% SEM or 47 of n = 104 molecules), S1_T1 SpCas9 (89.5 ± 3.1% SEM, or 85 of n = 95 molecules), S1_T2 SpCas9 (77.2 ± 4.7% SEM, or 61 of n = 79 molecules), S2_T0 SpCas9 (27.3 ± 5.1% SEM, 21 of n = 77 molecules) and S3_T0 SpCas9 (23.1 ± 5.2% SEM, or 15 of n = 65 molecules) (blue bars). Experiments combining S2-T0 SpCas9 and RNAP (58.7 ± 5.7% SEM, or 44 of n = 75 molecules), and S3_T0 SpCas9 and RNAP (52.3 ± 4.8% SEM, or 56 of n = 107 molecules) are also quantified (orange bars). (**b**) Normalized percentages of those R-loop-forming DNA molecules which go on to display terminal bead loss for: S1_T0 SpCas9 (55.1 ± 5.3%, n = 89), S1_T1 SpCas9 (97.7 ± 1.6%, n = 86), S1_T2 SpCas9 (93.8 ± 3.0%, n = 64), S2_T0 SpCas9 (27.6 ± 5.1%, n = 76) and S3_T0 SpCas9 (23.7 ± 5.5%, n = 59), are normalized by dividing the number of molecules that formed R-loops (cyan bars with SEMs). Similarly, the normalized percentages for S2_T0 SpCas9 plus RNAP (64.3 ± 5.7%, n = 70) and NT00-SpCas9 plus RNAP (59.4 ± 5.0%, n = 96) are shown in yellow bars. SEM was derived from the binomial distribution with a 95% confidence interval. Source data

**Extended Data Fig. 10:**
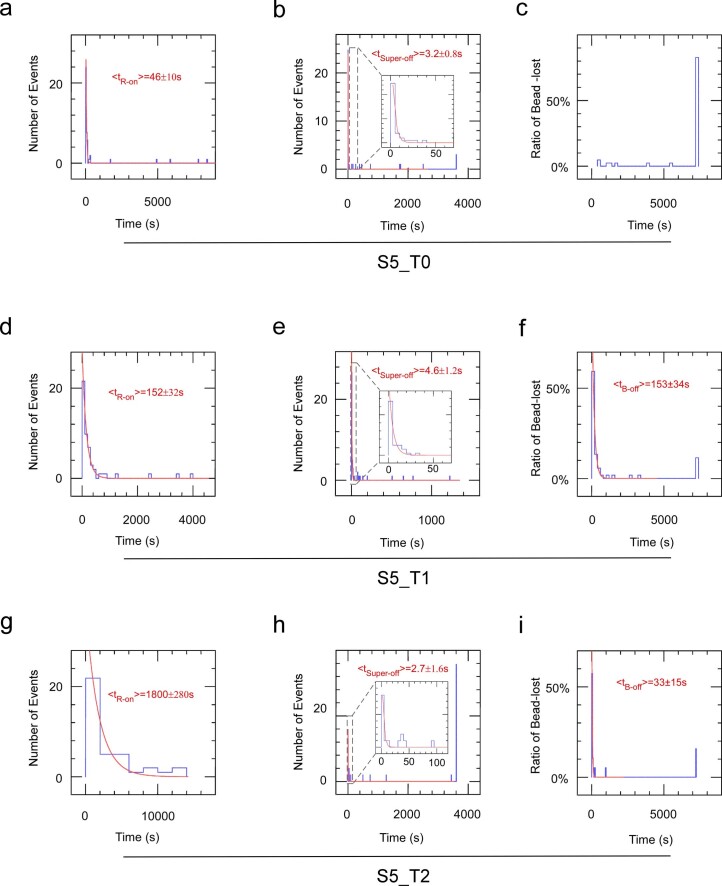
Comparing the kinetics of <t_R-on_>, <t_Super-off_> and <t_B-off_> on extended site S5 using canonical and PAM-shifted guides. (**a–c**) Canonical PAM S5_T0 RNPs generated (a) R-loops within 46 ± 10 s on average (n = 49), caused (b) loss of supercoiling within 3.2 ± 0.8 s on average (n = 47), and resulted in (c) a long-lasting post-catalytic complex as 83.3% of molecules were broken within a 7200-second window. (**d**–**f**) Shifted PAM S5_T1 RNPs generated (d) R-loops more slowly, that is within 152 ± 32 s on average (n = 52), caused € loss of supercoiling within 4.6 ± 1.2 s on average (n = 52) and resulted in (f) an unstable post-catalytic complex as 88.5% of molecules were broken within a 7200-second window. <t_B-off_> was characterized as 153 ± 34 s (n = 52). (**g-i**) Shifted-PAM S5_T2 RNPs generated (g) R-loops in the slowest manner, that is within 1800 ± 280 s on average (n = 38), caused (h) loss of supercoiling within 2.7 ± 1.6 s on average (n = 37) and resulted in (i) an unstable post-catalytic complex as 84.2% of molecules dissociated within a 7200 second window. <t_B-off_> was characterized as 33 ± 15 s (n = 19). See Supplementary Table 2 for specific parameters.

Consistent with this alternative explanation, the second key observation is that, for canonical S1_T0 and S2_T0 SpCas9 RNPs, <*t*_Bead-off_> was often too long to measure, typically greater than 7,000 s (Fig. 2c,l), which aligns with the findings of a recent single-molecule study^30^. In stark contrast, S1_T1 and S1_T2 complexes resulted in terminal bead loss in only hundreds of seconds (Fig. 2f,i). This higher efficiency of DSB induction using S1_T1 and S1_T2 SpCas9 than when using S1_T0 SpCas9 and S2_T0 SpCas9 RNPs is also somewhat counterintuitive given the bulk assay (Fig. 1b–d and Extended Data Fig. 9a), which showed comparable levels of DSBs. To monitor the efficiency of this reaction, we calculated the fraction of molecules that progressed from R-loop formation to apparent DSB induction by dividing the number of bead-loss events over a 2-h period by the total number of molecules displaying R-loop formation. With this metric, bead-loss fractions were still significantly higher in S1_T1 and S1_T2 than in S1_T0 and S2_T0 (Extended Data Fig. 9b). Analysis carried out on another site (S5, see Extended Data Fig. 10a–i) confirmed that the canonical PAM-mediated post-catalytic complex is more stable than the shift-PAM variation. These findings led us to predict that SpCas9 RNPs confirmed to have incised DNA (that is, those observed to be in the torsionally relaxed state), and in particular canonical SpCas9 complexes that result in relatively fewer lost beads, could perhaps harbor double-strand breaks held together by the SpCas9 RNP.

**Fig. 3:**
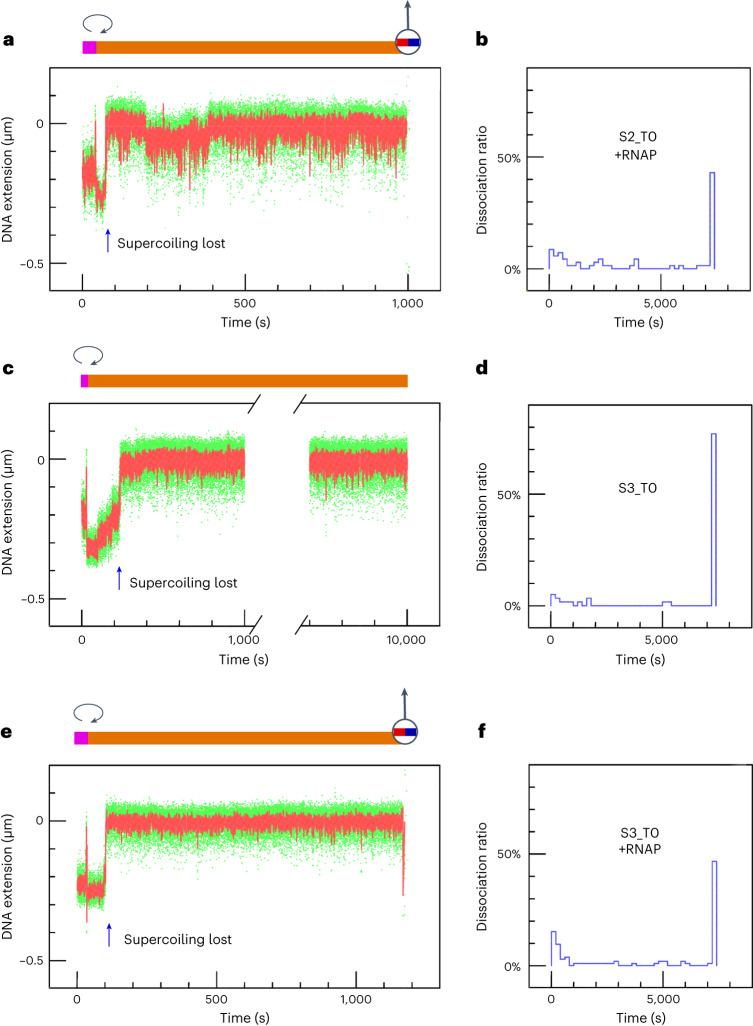
Observation and quantification of terminal bead loss for targeted DNA regions with canonical PAM spacing in the absence or presence of RNAP. **a**, Typical DNA extension time-trace, obtained using S2_T0 SpCas9 combined with RNAP. DNA was initially positively supercoiled by five turns to allow injection of the reagent to the sample chamber under conditions in which the reaction is inhibited, after which the DNA was unwound by eight negative turns (clockwise arrow) to permit the sequence of events including R-loop formation, supercoil loss (blue arrow), and terminal bead loss (as per Extended Data Fig. 7b). The moment of terminal bead loss is represented above the time-trace as a magnetic bead topped by an up arrow. The time-trace y-axis is labelled such that the maximal bead position above the surface is set to zero, and lower extensions are reported as negative values. **b**, The percentage of bead-loss events, normalized by the number of molecules that formed R-loops (as per Extended Data Fig. 7b), shows a bimodal distribution. **c**,**d**, As in **a** and **b**, but for S3_T0 SpCas9 without RNAP. No bead loss was observed on this time-trace. **e**,**f**, As in **c** and **d**, but for S3_T0 SpCas9 with RNAP. The mean bead-loss fractions observed within a 2-h window for S2_T0 SpCas9 plus RNAPs, S3_T0 SpCas9, and S3_T0 SpCas9 plus RNAP are 56.5% (*n* = 69), 22% (*n* = 59), and 52.4% (*n* = 103), respectively. All events lasting for more than 10,800 s are reported as 10,800 s. See Supplementary Table 3 for detailed values. Source data

To examine this, we took advantage of the fact that S2_T0 and S3_T0 SpCas9 target the template strand used by RNA polymerase to transcribe the nanomanipulated DNA. We reasoned that, if dissociation of the post-cleavage complex is the rate-limiting step in processing a target via canonical PAM usage, then transcription by RNA polymerase may result in collision with the post-cleavage complex, facilitating its dissociation. Indeed, transcribing RNAP almost doubled the fraction of beads lost (Fig. 3a–f and Extended Data Fig. 9a,b). We thus propose that the low rate of bead loss from canonical SpCas9 targeting is due to the relatively higher stability of the post-cleavage DNA–dgRNA–SpCas9 complex than that of shift-PAM targeted SpCas9.

### HNH domain stability protects incised DNA from rapid loss of supercoiling

Similarly, we propose that the faster supercoil dissipation observed in stressed SpCas9 complexes than in unstressed ones is due to the relatively higher stability of canonically spaced, unstressed SpCas9 complexes. To explore this hypothesis, we analyzed supercoil-loss times for unstressed and stressed HNH-SpCas9 and RuvC-SpCas9, which were described earlier^31^.

**Fig. 4:**
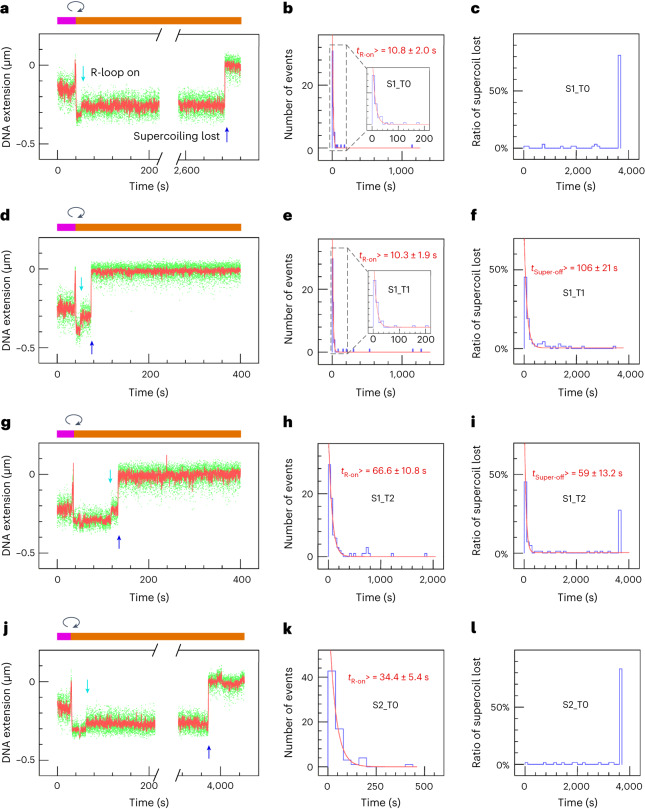
R-loop formation and supercoil loss in DNA-nicking assays using HNH-SpCas9 (D10A). **a**–**c**, The experiment carried out using S1_T0 HNH-SpCas9. **a**, Bead position time-trace. The magenta bar highlights the phase during which DNA is positively supercoiled for the injection of RNPs; the clockwise arrow denotes the moment at which the DNA is negatively supercoiled to initiate the reaction; and the orange bar highlights the phase during which the DNA is negatively supercoiled. The moment of R-loop formation is indicated by a vertical cyan arrow, and the moment of supercoil loss is indicated by a vertical blue arrow. The time-trace y-axis is labelled such that the maximal bead position above the surface is set to zero, and lower extensions are reported as negative values. **b**, Distribution of times for R-loop formation (marked by the cyan arrow in **a**) is fit to a single exponential distribution (red line), yielding the mean lifetime indicated. **c**, The distribution of time elapsed between R-loop formation and loss of supercoiling (marked by the blue arrow in **a**) is also fit to a single exponential distribution (red line), with the mean lifetime indicated. The subsequent panels are structured as in **a**–**c**, but different guide RNAs were used: S1_T1 HNH-SpCas9 (**d**–**f**); S1_T2 HNH-SpCas9 (**g**–**i**); and S2_T0 HNH-SpCas9 (**j**–**l**). For S1_T1 HNH-SpCas9 and S1_T2 HNH-SpCas9, the first modal, that is, the fast-dissociated supercoil-loss events, are fit to single exponential distributions (red line) with indicated lifetime of means (f,i). All events lasting for more than 3,600 s are reported as 3,600 s. The populations of molecules that remain supercoiled for >3,600 s are 81.4%, 0%, 28%, and 84.1% for S1_T0 HNH-SpCas9, S1_T1 HNH-SpCas9, S1_T2 HNH-SpCas9, and S2_T0 HNH-SpCas9, respectively. See Supplementary Table 3 for detailed values. Source data

**Fig. 5:**
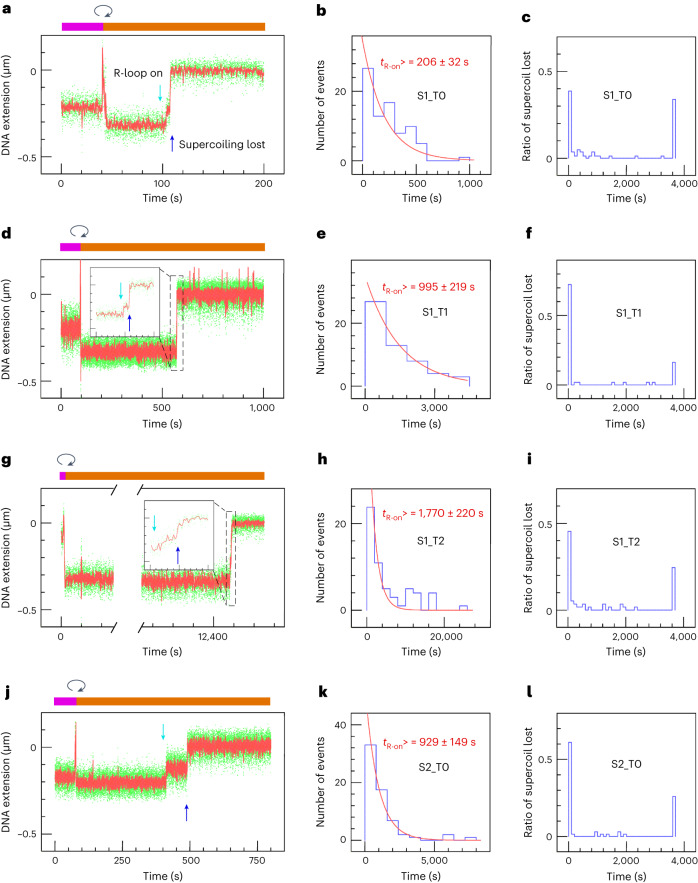
R-loop formation and supercoil loss in DNA-nicking assays using RuvC-SpCas9-H840A. The panels are structured as in Figure 4, but RuvC-SpCas9 was used. **a**–**c**, The experiment was carried out using S1_T0 RuvC-SpCas9. **a**, Bead position time-trace. The magenta bar highlights the stage at which DNA is positively supercoiled for the injection of RNPs; the clockwise arrow denotes the moment at which the DNA is negatively supercoiled to initiate the reaction; and the orange bar highlights the stage at which the DNA is negatively supercoiled. The moment of R-loop formation is indicated by a vertical cyan arrow, and the moment of supercoil loss is indicated by a vertical blue arrow. The time-trace y-axis is labelled such that the maximal bead position above the surface is set to zero, and lower extensions are reported as negative values. **b**, Distribution of times for R-loop formation (marked by the cyan arrow in **a**) is fit to a single exponential distribution (red line), yielding the indicated mean lifetime. **c**, The distribution of times elapsed between R-loop formation and loss of supercoiling (marked by the blue arrow in **a**) is also fit to a single exponential distribution (red line), with the mean lifetime indicated. The subsequent panels are structured as in **a**–**c**, but different guide RNAs were used: S1_T1 RuvC-SpCas9 (**d**–**f**); S1_T2 RuvC-SpCas9 (**g**–**i**); and S2_T0 RuvC-SpCas9 (**j**–**l**). The steps of R-loop formation (cyan arrow) and supercoil loss (blue arrow) are readily distinguished in the time-traces for S1_T0 and S2_T0, but occur in more rapid succession and are thus only resolved in the insets presented for S1_T1 and S1_T2 RuvC-SpCas9 catalytic events. All events lasting more than 3,600 s are reported as 3,600 s. The populations of molecules that remain supercoiled for >3,600 s are 34.6%, 16.4%, 24.6%, and 26.2% for S1_T0 RuvC-SpCas9, S1_T1 RuvC-SpCas9, S1_T2 RuvC-SpCas9, and S2_T0 RuvC-SpCas9, respectively. See Supplementary Table 3 for detailed values. Source data

Results obtained using HNH-SpCas9 closely resemble those obtained with wild-type SpCas9, with PAM-shifting resulting in an increase in time required to form the R-loop and a decrease in time required to observe loss of supercoiling (Fig. 4). PAM-shifting with RuvC-SpCas9 also resulted in an increase in time required to form the R-loop and a decrease in time required to observe loss of supercoiling, although it is interesting to note that with this variant, a significant fraction of molecules displayed rapid loss of supercoiling even with the non-shifted PAM (Fig. 5 and Supplementary Table 3). Differences in the rates of R-loop formation between wild-type SpCas9 and the variants should not be interpreted, because they may simply be attributed to differences in specific activities because the enzyme preparations are distinct.

The rate of supercoil loss using the wild-type complex and its progressive increase as the complex is shifted tracks the rate of supercoil loss using the RuvC–SpCas9 complex and its progressive increase as the mutant complex is shifted. Increases in the rate of supercoil loss obtained by shifting HNH-SpCas9 do not appear to contribute to increases in the rate of supercoil loss obtained by shifting wild-type complexes. Yet the higher rate of supercoil loss by shifted HNH-SpCas9 as compared to unshifted HNH-SpCas9 contrasts with the real comparable incision efficiencies that this mutant displays in ensemble assays (using SDS or heat denaturation). This suggests that HNH-SpCas9 helps to maintain supercoiling even after it performs strand cleavage, presumably because of the RNA ‘splint’ and contacts between RNP and the downstream PAM, and that straining the RNP complex with shifted PAM spacing causes it to rapidly lose its grip on the incised yet still supercoiled DNA. This is consistent with the fact that increases in the rate of supercoil release that occur after shifting HNH-SpCas9 track increases in the rate of bead release obtained by shifting wild-type SpCas9. These results point to destabilization and disassembly, via conformational distortion of the post-catalytic complex at the level of the R-loop itself, as a key rate-limiting feature of the complete processing reaction.

## Discussion

**Fig. 6:**
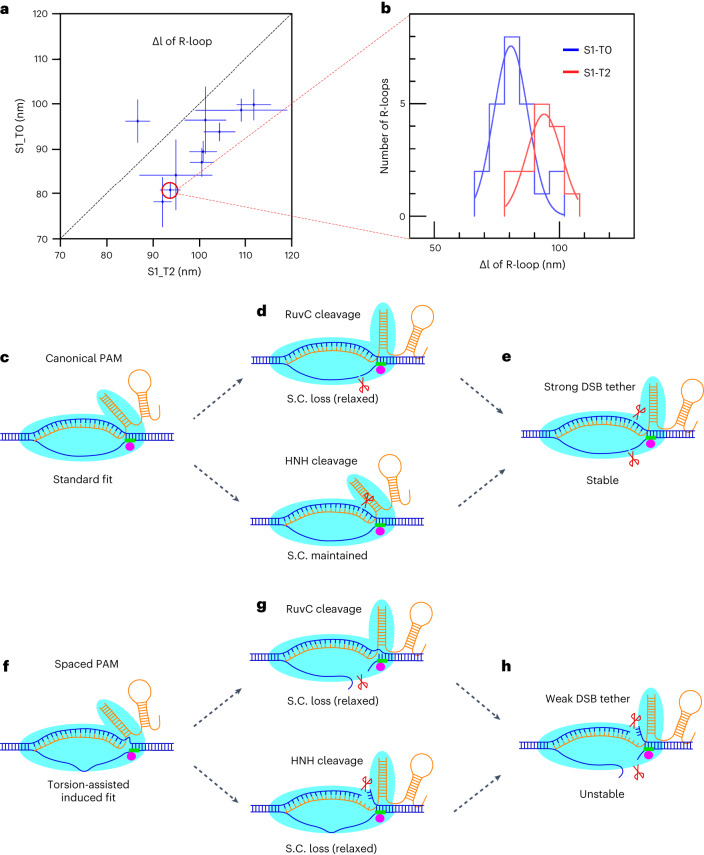
DNA scrunching in shift-PAM complexes and a model for catalytic steps on target sequences with canonical and shifted PAMs. **a**, Correlation between mean extension changes, Δl, observed on the same DNA molecules forming R-loops with S1_T0 or S1_T2 dSpCas9. Error bars represent s.e.m. **b**, Histograms of DNA extension changes observed on a given DNA molecule forming R-loops with S1_T0 or S1_T2 RNPs are fit to Gaussian distributions, yielding 81 ± 2 nm (s.e.m., n = 26 events) and 94 ± 3 nm (s.e.m., n = 19 events), respectively, corresponding to an extra ~2 bp of unwinding for the S1_T2 dSpCas9-based R-loops (see Methods). A minor fraction (15%) of presumably immature R-loops with only about half of the full unwinding, only observed using dSpCas9, were excluded from analysis. **c**–**e**, Model for catalytic steps on target sequence with canonical PAMs. **c**, The first 20 nt of crRNA forms an R-loop with the complementary strand, and the PAM interacts with the PAM-interacting motif of SpCas9. **d**, The complementary strand can be incised by the HNH nuclease (bottom) and the non-complementary strand can be incised by the RuvC nuclease (top). Incision of the complementary strand by the HNH nuclease does not favor loss of supercoiling (S.C.) because the RNA:DNA hybrid acts as a splint across the cut site. By contrast, incision of the non-complementary strand by the RuvC domain tends to result in loss of supercoiling. **e**, Upon cleavage of both strands, the stable post-cleavage complex can tether the broken DNA ends for a long period of time. **f**–**h**, Model for catalytic steps on target sequence with shifted PAMs. **f**, In the pre-catalytic complex formed with a shifted PAM, mechanical strain slows down R-loop formation and PAM binding. **g**, However, mechanical strain also results in the instability of the complex, with signature loss of supercoiling upon incision by the HNH domain (bottom) as well as the RuvC domain (top). **h**, Once double-strand cleavage is completed, the strained, destabilized post-cleavage complex holds the broken DNA ends together more weakly, favoring terminal separation of DSB ends. Graphical code: DNA, blue; crRNA–tracrRNA duplex, orange; PAM, green; cleavage sites, scissors; SpCas9, cyan rabbit-like shape; PAM-interacting motif, magenta dot. Source data

In this study, we characterize shifted PAM-mediated DNA processing by SpCas9. We propose that when NGG PAMs are not canonically positioned adjacent to the RNA-guided target sequence, SpCas9 retains the ability to ‘scrunch’ several nucleotides of substrate^32^, achieving double-strand DNA cleavage by off-set incision of the two strands and destabilizing the post-cleavage complex. Consistent with this ‘scrunching’ hypothesis for R-loop formation using shifted PAMs, we compared the size of the R-loops generated by canonical (S1_T0) and shifted (S1_T2) SpCas9–DNA complexes and found that the those generated by the latter are ~2 bp larger, as predicted, than those generated by the former (Fig. 6a,b)^33,34^.

Using bulk assays and sequencing assays, we show that shifted SpCas9 complexes generate DNA overhangs of up to a few bases in length. Even as strain increases, HNH domain incision of the complementary strand faithfully tracks the RNA-DNA hybrid position as it moves away from the PAM, maintaining incision at 17 nt from the 5′ end of the hybrid. Similarly, RuvC incision remains adjacent to the PAM for shifts of up to 2 bases, resulting in robust generation of a 3′ overhang of up to 2 bases at the DNA ends. However, beginning at shifts of 3 bases, RuvC domain incision ‘slips’ from PAM-adjacent to HNH-adjacent, presumably highlighting the distinct sets of interactions between the RuvC domain and the HNH domain and the RuvC domain and the PID. The RuvC domain can still incise the non-complementary strand in the ‘slipped’ state, but these incision events face the HNH incision site and once again result in formation of a blunt overhang. In human cells, the 1-bp-shifted PAMs lead to robust DSBs; however, 2-bp-shifted PAMs generate much weaker cleavage, probably owing to the nucleosome-bound and less-negatively-supercoiled genomic DNA in cells.

Single-molecule interrogation of the kinetics of R-loop formation, DNA single-strand incision, and DNA double-strand cleavage by SpCas9 and its nuclease domain mutants in the context of canonical and shift-PAM targeting provides us with a deeper understanding of SpCas9 cleavage and how the pre-catalytic and post-catalytic complexes are affected by offsetting the guide and PAM spacing. This is consistent with earlier findings that the DNA bubble within the seeding region can tolerate RNA-DNA mismatches^11^. Taken together, these results lead us to propose a unified catalytic mechanism of SpCas9 action that is modulated by the spacing between the PAM and the R-loop. For canonically spaced PAMs (Fig. 6c–e), PID binding contributes to stabilizing the pre-catalytic complex (Fig. 6c) and is followed by complementary-strand cleavage by the HNH domain (Fig. 6d, bottom) or non-complementary strand cleavage by the RuvC domain (Fig. 6d, top) in a stochastic manner. Although HNH nuclease incision within the R-loop does not lead to the immediate loss of supercoils, presumably because the RNA can act as a ‘splint,’ RuvC nuclease incision in the non-complementary strand leads to rapid loss of supercoils. Once both strands are incised, the two broken DNA ends remain tethered owing to the overall stability of the complex bridging the break (Fig. 6e). In the case of shifted PAMs, additional mechanical stress is introduced in the R-loop state, overcoming the stabilizing effect of the splint and resulting in rapid loss of supercoils, even upon HNH-domain incision and rapid dissociation of the post-catalytic complex once both strands have been cleaved (Fig. 6f–h).

In accordance with our model, recent studies have determined that the complementary strand is more stable than the non-complementary strand in the post-catalytic complex^35,36^. From the single-molecule and bulk assays carried out here, we conclude that SpCas9-mediated double-strand cleavage of DNA does not necessarily lead to rapid dissociation of broken ends. Supercoiling is rapidly lost in unshifted, wild-type Cas9, with both RuvC and HNH catalytic activities participating in the first incision event. The rate of supercoiling loss increases with shifting. For unshifted SpCas9 in just over half of all events, secondary incision appears to be hidden from detection by SpCas9 remaining on the DNA break, most likely bridging the break by maintaining the R-loop and the PID engaged in the post-incision complex. The complex can be destabilized by altering the spacing between the RNA-DNA hybrid and the PAM, leading to a much higher rate of terminal bead loss for shifted complexes. The complex can also be driven off DNA using transcribing RNA polymerase. Shifted complexes thus result in enhanced disassembly from DNA, which here appears to be a key rate-limiting step to DNA processing.

Our work complements and extends previous studies demonstrating that SpCas9 is much less tolerant of guide-DNA mismatches in the PAM-proximal seeding region than those in PAM-distal non-seed regions^13^, and that directed mutations in SpCas9’s PAM-interacting domain can result in variants preferring non-canonical PAM combinations^16,17,19^. The shift-PAM targeting described here further expands options when designing sgRNAs and will be useful when applied in various genome-editing contexts.

The combination of ensemble assays, next-generation sequencing tools and single-molecule studies presented here allowed us to dissect the sequence of steps from R-loop formation, to single-strand-break induction, to double-strand-break formation, to post-incision complex dissociation by SpCas9 acting on its target DNA using both canonical and strained R-loop–PAM spacing. Our results demonstrate that the protein–DNA complex is sufficiently deformable as to tolerate modulation of the distance between the PAM and the seed region, and that this is sufficient to cause a single mechanically stressed dgRNA–SpCas9 to generate overhanging ends and dissociate rapidly. This provides an additional degree of freedom in choosing SpCas9 targets, and an added layer to discern potential off-target effects during design of guide RNAs.

### Limitations of the study

The single-molecule assays revealed that post-catalytic complex dissociation is a rate-limiting step for separating DNA ends, and that shifted PAMs speed up dissociation. To directly visualize the SpCas9 dynamics and to determine whether SpCas9 remains on either of the two ends after end separation, other single-molecule tools, including total internal reflection fluorescence microscopy and fluorescence resonance energy transfer, need to be used.

SpCas9 can generate short, templated insertions, likely involving staggered cleavage and 5′ overhangs, but the molecular mechanism underlying this remains unclear^24,37–41^. Cleavage assays in living cells combined with LAM-HTGTS show that both canonical PAM and 1-bp-shifted PAM generate comparable cleavage frequencies, although larger shifts significantly decrease cleavage efficiency. Thus, more effective shift-PAM engineering would likely be necessary to potentially enhance gene knock-in efficiency.

## Methods

### Cell lines

HEK293T cells were obtained from ATCC and cultured at 37 °C and 5% CO_2_ in DMEM supplemented with 10% (vol/vol) FCS, 50 U mL^–1^ penicillin–streptomycin, 2 mM l-glutamine, 1× MEM-NEAA, 1 mM sodium pyruvate, 50 μM 2-mercaptoethanol, and 20 mM HEPES (pH 7.4). HEK293T cells were maintained in 2.5% FCS (vol/vol) DMEM medium only in the period of 2 h before transfection and 6 h post-transfection.

The K562-iCas9-GFP cell line was established by infecting the K562 cells (originated from ATCC) with lentivirus-carrying doxycycline-inducible SpCas9-T2A-GFP and BSD gene segments. Briefly, the donor plasmid was engineered by replacing the NeoR of Lenti-iCas9-Neo (Addgene no. 85400)^42^ with BSD. Infected K562 cells were seeded into 96-well plates at a concentration of 0.3 cells well^–1^. Cell colonies were picked up 10 d later and subjected to genotyping, fluorescence microscopy, flow cytometry, and western blotting to choose a stable K562-iCas9-GFP cell line. The K562-iCas9-GFP cells were maintained in Roswell Park Memorial Institute (RPMI)-1640 medium supplemented with 10% (vol/vol) FCS, 50 U mL^–1^ penicillin–streptomycin, 2 mM l-glutamine, 1× MEM-NEAA, 1 mM sodium pyruvate, 50 μM 2-mercaptoethanol, 20 mM HEPES (pH 7.4), and 10 μg mL^–1^ blasticidin; 1 μg mL^–1^ doxycycline was added only when expression of Cas9 was required. Doxycycline-treated cells were not used in experiments, to preserve the cell line.

### Reconstitution of RNP and RNAP particles in vitro

The crRNAs and tracRNA (see Supplementary Table 4) were mixed 1:1 to 1 μM in supplied buffer 3.1 (NEB) with 0.2 U µL^–1^ RNaseOUT (Invitrogen), denatured at 90 °C for 2 min, and cooled at room temperature for 30 min to allow RNA duplex dgRNA to form. SpCas9 and its mutants were added in a 1:1 ratio with the dgRNA dimer and incubated at room temperature for another 30 min to allow the formation of stable RNP particles.

*Escherichia coli* RNAP core subunits and σ factor were expressed, purified, and reconstituted in holo-RNAP enzymes, as has been described^43^.

### DNA substrate preparation

The targeting DNA is a transcript unit of about 200 bp (shown in Extended Data Fig. 1), which was inserted into the unique Kpn1 site of the Charomid-9-5 vector. The targeting DNA transcript, with ~1-kb arms flanking each site, was amplified by PCR to obtain 2.2-kb DNA inserts and was sub-cloned into the SbfI and XbaI sites of the pUC18 vector^20^. The pUC18 bearing the 2.2-kb DNA inserts and its SbfI-linearized products were the substrates used for the ensemble assays. The site-directed mutants were generated by paired PCR and confirmed by Sanger sequencing.

The pUC18 plasmids bearing 2.2-kb targeting DNA were transformed, amplified using *E. coli* Turbo (NEB), and extracted by NucleoBond midi-prep (Macherey Nagel). The plasmid was further purified by 1% agarose gel electrophoresis, and a NucleoSpin kit (Macherey Nagel) was used to obtain supercoiled double-stranded circular plasmids. Linearized DNA substrates were generated by treating the supercoiled plasmids with SbfI and were purified using 1% agarose gel electrophoresis and a gel extraction kit.

### Ensemble biochemistry assays

Supercoiled and linearized plasmids were mixed with dgRNA–SpCas9 or dgRNA–SpCas9 mutants to a final concentration of 10 nM (substrate) and 100 nM (RNPs) in 10 µL of reaction buffer (20 mM HEPES-K pH 7.8, 150 mM KCl, 5 mM MgCl_2_, 2 mM DTT, 0.5 mg mL^–1^ BSA, 0.05% Tween-20, 0.2 U µL^–1^ RNaseOUT), which was incubated at 34 °C. The reactions were quenched by adding 6× loading dye (with 1% SDS) and were incubated at 65 °C for 3 min before 1% agarose-Etbr gel electrophoresis was run to detect the cleavage efficiency using the ChemiDoc system (Bio-Rad).

### Junction mapping via Sanger sequencing

For the In-Del assay, the supercoiled plasmids were mixed with dgRNA–SpCas9 to a final concentration of 10 nM (substrate) and 100 nM (RNP) in a final volume of 100 μL, and were incubated at 37 °C overnight. Then, 6× loading dye was added and incubated at 65 °C for 3 min; this was followed by 1% agarose gel electrophoresis to purify the plasmid with DSBs. The purified DNA molecules were treated with a quick-blunting kit (NEB) and ligated using either the quick-ligase kit (NEB) or T4-DNA ligase (NEB) following the corresponding protocols. The re-ligated plasmids were transformed into Turbo competent cells (NEB), incubated on ice for 30 min, heat-shocked at 42 °C for 45 s, cooled on ice for another 2 min, briefly recovered in SOC (super optimal broth with catabolite repression) medium at 37 °C for 10 min, and finally spread on LB agar with ampicillin and incubated at 37 °C overnight. The next day, single colonies were picked, amplified, and plasmid-purified for Sanger sequencing.

For the 5′-locating assay, all the procedures are the same as for the In-Del assay, except the substrate DNA molecules were SbfI-linearized after SpCas9 cleavage. The cleaved products by dgRNA–SpCas9 RNPs were about 4 kb and 1 kb, and the 4 kb DNA molecules were subjected to the same blunting, re-ligation, transformation, and sequencing as described above. Because the 5′ ends of cleaved DNA products would be fixed, the 5′ ends could be determined.

The In-Del assay can be used to obtain single-nucleotide resolution in the case of deletion or insertion of nucleotides in a ATGC-diverse micro-locus (S4_T2 in Supplementary Table 2); however, it cannot be used to determine the precise cleavage site in the cases dominated by perfect repair (S2_T0, S1_T0, S1_T4 and S1_T-1 in Supplementary Table 2) or in the cases of insertion or deletion of nucleotides in a micro-locus with consecutively identical nucleotides (S1_T1, S1_T2, S1_T3 and S5_2 in Supplementary Table 2). Thus the 5′-locating assay is required to rule out other cleavage possibilities and to pinpoint the cleavage sites.

### DSB detection in living cells by LAM-HTGTS

HEK293T cells were split at 30% confluence in a 6-well plate, cultured in DMEM medium with 10% FCS. After 16 h, the culture medium was replaced by low serum medium DMEM with 2.5% FCS and maintained for 1–2 h. Meanwhile, plasmid PEI (polyethylenimine) mixture was prepared. In brief, 15 µL PEI (1 mg mL^–1^) was added into 110 µL NaCl (150 mM) to form PEI working solution, and in parallel, pX330 plasmid (about 1 μg µL^–1^ each) bearing three T0-sgRNAs, RAG1B_T0, RAG1C_T0, and RAG1D_T0 (Supplementary Table 5), was mixed with bait-L or bait-A in a ratio of 1:1:1:2 (for bait-L, 5 μg:5 μg:5 μg:10 μg; for bait-A, 4 μg:4 μg:4 μg:8 μg) and NaCl (150 mM) was added to a final volume of 125 µL to form DNA working solution. The PEI working solution was added into DNA working solution drop by drop and mixed well, and then was incubated at RT for 20 min and distributed evenly into the HEK293T cell culture. The plasmid PEI mixtures for T1 sgRNA, T2 sgRNA, and the bait-alone control were prepared and distributed into HEK293T cell culture. After 6 h, the low serum medium was replaced by growth medium, DMEM with 10% FCS. After 3 d, the HEK293T cells were collected and lysed, and genomic DNA was extracted. This was also performed for prey RAG1K–RAG1O (3 μg each), using RAG1D_T0 as bait (6 μg).

For LAM-HTGTS, 50 μg genomic DNA in each treatment condition was prepared in one library with a unique barcode, as has been described^22^. In brief, the genomic DNA was sheared by sonication to form fragments between 200 bp and 2 kb; this was followed by LAM-PCR using a biotin-labeled primer close to the bait site. The products from the LAM-PCR were attached to streptavidin magnetic beads and subjected to adapter ligation. The ligated products were further subjected to nested PCR using one primer (AP2I7) that matched the adapter and another (nested primer with a barcode of several nucleotides) that matched the region between the bait site and biotin-labeled primer. This was followed by tagged PCR using primer P7I7 and P5I5, which match the primers used in the nested PCR. The products ranging between 500 bp and 1 kb from tagged PCR were purified using 1% agarose gel and subjected to quality control and NovaSeq (Novogene). See Supplementary Table 6 for oligonucleotide sequences.

### Western blotting

The HEK293T control cells and those transfected with various combinations of bait and prey guide RNAs were collected 3 d post-transfection. Five million cells were lysed. The indicated antibodies (anti-Flag, 1:2,000, 12793S, Cell Signaling Technology; anti-Rabbit-IgG, 1:2,000, G-21234, Thermo Scientific; anti-β-actin, 1:4,000, SC-47778, Santa Cruz Biotechnology) and horseradish peroxidase (HRP) substrate (K-12042-D10, Advansta) were used for the western blotting experiments.

### GFP knock-out and detection by flow cytometry

The genes of sgRNAs GFP_T0, GFP_T1, GFP_T2, and RAG1L_T0 were cloned into the pMCB320 plasmid (Addgene no.89356)^44^. Then, 5 μg each of the GFP_T0-, GFP_T1-, GFP_T2-, and RAG1L_T0-pMCB320 plasmids was delivered into K562-iCas9-GFP cells by 4D-nucleofector (Lonza). Transduced cells were recovered in RPMI medium with 10% FCS, 0.2 mg mL^–1^ G418, 5 μg mL^–1^ blasticidin, and 1 μg mL^–1^ doxycycline. The nucleofection efficiencies were measured at 2 d post-nucleofection as ~60% for all conditions by flow cytometry (BD), using mCherry in the pMCB320 plasmid. The efficiency of GFP knock-out was measured on day 7 post-nucleofection by flow cytometry.

### Single-molecule manipulation

The 2.2-kb DNA was ligated with 1 kb biotin- and dig-labeled DNA through SbfI and XbaI overhangs by T4 DNA ligase (NEB). The ligated products were attached to the anti-dig-treated surface of the reaction chamber and streptavidin-coated magnetic beads. The attached DNA constructs were the substrates used in single-molecule assays. Each of the SpCas9 components, including dgRNA alone, dgRNA–SpCas9, and dgRNA–SpCas9 mutants, were present at a concentration of 1 nM. In the transcription-enhanced cleavage assay, 200 pM RNAP and 100 μM NTPs were also introduced.

DNA hat curves were taken at 0.2 pN to distinguish the intact double-stranded DNA, nicked DNA, and tangled DNAs on the basis of biophysical properties, including DNA elasticity and extension^27^. Only the intact double-stranded DNA molecules were used in the following single-molecule experiments. The extension of intact double-stranded DNA molecules was then re-calibrated at 0.2 pN extending force for 8 turns of negative supercoiling, no supercoiling, and 5 turns of positive supercoiling to double-check initial calibration and generate references of DNA extension. Following that, the applied force was increased to 1 pN (the DNA molecules remain with 5 turns of positive supercoiling), and the sample including dgRNA alone, dgRNA–SpCas9, or dgRNA–SpCas9 mutants was introduced in the reaction chamber in each assay. A fluctuation spike was caused by the flow of sample injection. When the reaction system became stable, the force was lowered back to 0.2 pN. The DNA molecules were either kept at 5 turns of positive supercoiling or 8 turns of negative supercoiling to observe R-loop formation, loss of supercoiling, and loss of the magnetic bead.

### Biochemistry data analyses

The cleavage efficiencies of DSBs and SSBs in EtBr-agarose gels were quantified using ImageJ and normalized by subtracting the backgrounds of controls. The efficiencies were further analyzed, and figures were plotted using GraphPad.

### Single-molecule data analysis

Single-molecule data were processed using the PicoJAI software suite (PicoTwist SARL).

#### Temporal analysis

The time required for R-loop formation or dissociation, the time required to transition from the R-loop state to the torsionally relaxed state (loss of supercoiling), and the time required to transition from the torsionally relaxed state to irreversible loss of the magnetic bead, were characterized at the level of individual molecular events by analyzing DNA extension versus time traces obtained on individual DNA molecules. The mean dwell times for the corresponding states observed in pre- and post-catalytic complexes were obtained by fitting the resulting distributions of times to single exponential decays. Python and GraphPad were also used to compare the significance level of different treatments.

#### Spatial analysis

Nanometer-scale changes in DNA extension during R-loop formation and dissociation were characterized at the level of individual molecular events by analyzing DNA extension versus time traces obtained on individual DNA molecules, and were then converted to DNA unwinding in base-pairs by converting changes in DNA writhe to changes in DNA unwinding via the linking number formalism, as has been described previously^32,33^, using the calibrated change in DNA extension (typically ~55 nm per unit of writhe in our experimental conditions of force and salinity, see Extended Data Fig. 7a) and a DNA pitch of 10.5 bp turn^–1^. Thus, for instance, since the seeding sequence of each crRNA is 20 nt, in theory the fully hybrid RNA-DNA (mature R-loop) unwinds approximately two turns of DNA, resulting in the addition of two units of positive writhe into the DNA and a concomitant increase in the extension of negatively supercoiled DNA of about 110 nm. Following single-strand DNA incision, the DNA molecules have the potential to reach the torsionally relaxed state (complete loss of supercoiling, corresponding to the maximum-extension state seen in Extended Data Fig. 7a), and following cleavage of both strands, the DNA molecules have the potential to break, as seen by irreversible loss of the magnetic bead.

### LAM-HTGTS data analyses

LAM-HTGTS data analysis was conducted as previously reported^22^. In brief, illumina reads were de-multiplexed and adapter sequences were trimmed using the fastq-multx tool from ea-utils and the SeqPrep utility, respectively. Reads were normalized by Seqtk and mapped to the hg19 reference genome using TranslocWrapper to map translocations. Reads were also aligned to the bait region using HTGTS Rep to identify rejoining events and were then combined to translocations using Rejoin. Duplicates of translocations were removed to eliminate the artificial duplication of PCR, with features of exact bait sequence and exact prey sequence, which may not have been perfect because some of them might be true biological duplications.

### Reporting summary

Further information on research design is available in the Nature Portfolio Reporting Summary linked to this article.

## Online content

Any methods, additional references, Nature Portfolio reporting summaries, source data, extended data, supplementary information, acknowledgements, peer review information; details of author contributions and competing interests; and statements of data and code availability are available at 10.1038/s41594-023-01104-6.

### Supplementary information

Supplementary Information Supplementary Fig. 1.

Reporting Summary

Supplementary Tables 1–6 1. Information for libraries obtained by LAM-HTGTS. 2. In-Del and 5′-locating data obtained by Sanger sequencing. 3. Summary of mean lifetimes (or duration, DUR) obtained in single-molecule experiments. 4. Guide RNA oligonucleotides used for ensemble biochemistry and single-molecule assays. 5. DNA oligonucleotides used for point mutagenesis and Sanger sequencing (black and red) and DNA oligonucleotides used for cloning sgRNA genes into pX330-SpCas9 and pMCB320 vectors (blue). 6. Oligonucleotides used for Nova-seq library preparation.

Supplementary Data 1 Single molecule time traces.

### Source data

Source Data Figs. 2–5,6 Single-molecule statistical source data.

Source Data Extended Data Figs. 1, 8, and 9 Single molecule statistical source data.

Source Data Fig. 1 Unprocessed gels and blots.

Source Data Extended Data Figs. 1–3 and 5 Unprocessed gels and blots.

## Data Availability

LAM-HTGTS sequencing data have been deposited at the Gene Expression Omnibus with the accession number GSE192459. Raw single-molecule time traces are available upon reasonable request. Source data are provided with this paper.

## Code availability

The LAM-HTGTS analysis pipeline and its associated utilities’ source codes are openly accessible through the following links:

LAM-HTGTS: https://github.com/JinglongSoM/LAM-HTGTS

Fastq-multx: https://expressionanalysis.github.io/ea-utils/

SeqPrep: https://github.com/jstjohn/SeqPrep

Seqtk: https://github.com/lh3/seqtk

For the xvin program used in single-molecule data analysis, researchers can obtain access by reaching out to the lead corresponding author.
