# Author Correction: Shifted PAMs generate DNA overhangs and enhance SpCas9 post-catalytic complex dissociation

**DOI:** 10.1038/s41594-024-01247-0

**Published:** 2024-03-05

**Authors:** Jinglong Wang, Julien Le Gall, Richard L. Frock, Terence R. Strick

**Affiliations:** 1 Institut Jacques Monod, Université de Paris Cité, Paris, France; 2 Institut de Biologie, Ecole Normale Supérieure, Université PSL, Paris, France; 3 Department of Radiation Oncology, School of Medicine, Stanford University, Stanford, CA USA; 4 Programme « Equipe Labélisée » de la Ligue Nationale Contre le Cancer, Paris, France; 5 Present Address: School of Medicine, Stanford University, Stanford, CA USA

**Keywords:** Single-molecule biophysics, Enzyme mechanisms, Biophysical methods, DNA restriction-modification enzymes

Correction to: *Nature Structural & Molecular Biology* 10.1038/s41594-023-01104-6, published online 12 October 2023.

In the version of the article initially published, there was an error in the NIH grant number in the Acknowledgements section, which has now been amended to read “R01 GM130942” in the HTML and PDF versions of the article.

