# Supplementary material for: Shifted PAMs generate DNA overhangs and enhance SpCas9 post-catalytic complex dissociation
Source: Nat Struct Mol Biol. 2023 Oct 12;30(11):1707–18. doi: 10.1038/s41594-023-01104-6 (PMC10643121; doi:10.1038/s41594-023-01104-6)
Supplement: Supplementary file 1 — Supplementary Fig. 1. [file 41594_2023_1104_MOESM1_ESM.pdf]

nature structural & molecular biology

Article

https://doi.org/10.1038/s41594-023-01104-6

Shifted PAMs generate DNA overhangs and enhance SpCas9 post-catalytic complex dissociation

In the format provided by the authors and unedited

nature structural & molecular biology

Article https://doi.org/10.1038/s41594-023-01104-6

Shifted PAMs generate DNA overhangs and enhance SpCas9 post-catalytic complex dissociation

In the format provided by the authors and unedited

SUPPLEMENTARY INFORMATION

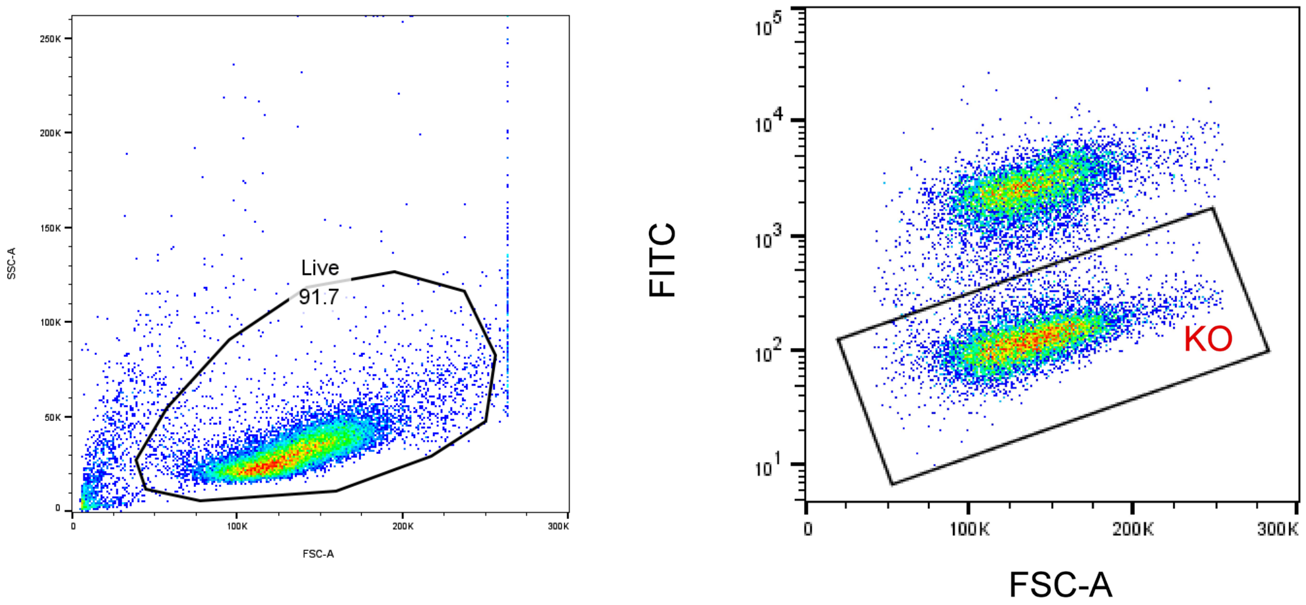

Supplementary Figure 1. FACS Gating Strategies. In a first step (left panel), we select 91.7% live cells as defined by the black contour line of the SSC-A vs FSC-A plot. In a second step (right panel) we detect GFP expression using the FITC channel and compare it to the FSC-A channel. We thus identify live cells expressing GFP and thus which have successfully carried out CRISPR-induced rejoining. Live cells which do not express GFP are indicated as “KO”. The right panel given as example corresponds to the GFP_T0 condition presented in Extended Data Fig. 4a (second panel from the left).
