# Supplementary material for: Shifted PAMs generate DNA overhangs and enhance SpCas9 post-catalytic complex dissociation
Source: Nat Struct Mol Biol. 2023 Oct 12;30(11):1707–18. doi: 10.1038/s41594-023-01104-6 (PMC10643121; doi:10.1038/s41594-023-01104-6)
Supplement: Supplementary file 2 — Reporting Summary [file 41594_2023_1104_MOESM2_ESM.pdf]

nature portfolio

Corresponding author(s): Terence Strick

Last updated by author(s): May 16, 2023

Reporting Summary

Nature Portfolio wishes to improve the reproducibility of the work that we publish. This form provides structure for consistency and transparency in reporting. For further information on Nature Portfolio policies, see our Editorial Policies and the Editorial Policy Checklist.

Statistics

For all statistical analyses, confirm that the following items are present in the figure legend, table legend, main text, or Methods section.

n/a	Confirmed	
☐	☒	The exact sample size (*n*) for each experimental group/condition, given as a discrete number and unit of measurement
☐	☒	A statement on whether measurements were taken from distinct samples or whether the same sample was measured repeatedly
☒	☐	The statistical test(s) used AND whether they are one- or two-sided<br>*Only common tests should be described solely by name; describe more complex techniques in the Methods section.*
☒	☐	A description of all covariates tested
☒	☐	A description of any assumptions or corrections, such as tests of normality and adjustment for multiple comparisons
☐	☒	A full description of the statistical parameters including central tendency (e.g. means) or other basic estimates (e.g. regression coefficient) AND variation (e.g. standard deviation) or associated estimates of uncertainty (e.g. confidence intervals)
☒	☐	For null hypothesis testing, the test statistic (e.g. *F*, *t*, *r*) with confidence intervals, effect sizes, degrees of freedom and *P* value noted<br>*Give P values as exact values whenever suitable.*
☒	☐	For Bayesian analysis, information on the choice of priors and Markov chain Monte Carlo settings
☒	☐	For hierarchical and complex designs, identification of the appropriate level for tests and full reporting of outcomes
☒	☐	Estimates of effect sizes (e.g. Cohen's *d*, Pearson's *r*), indicating how they were calculated

Our web collection on statistics for biologists contains articles on many of the points above.

Software and code

Policy information about availability of computer code

Data collection	Single-molecule data were collected using the PicoJai software suite (PicoTwist S.A.R.L, v. TS-2020); LAM-HTGTS data were collected by Illumina NovaSeq 6000.
Data analysis	Data were analyzed using the Xvin and PIAS software suites (PicoTwist S.A.R.L., 2019 version); IGV (version 2.8.0); FlowJo (version 10.8); GraphPad (version 9.3.0); and Image J (no version info). For code for LAM-HTGTS, please refer to https://github.com/JinglongSoM/LAM-HTGTS

For manuscripts utilizing custom algorithms or software that are central to the research but not yet described in published literature, software must be made available to editors and reviewers. We strongly encourage code deposition in a community repository (e.g. GitHub). See the Nature Portfolio guidelines for submitting code & software for further information.

Data

Policy information about availability of data

All manuscripts must include a data availability statement. This statement should provide the following information, where applicable:

- Accession codes, unique identifiers, or web links for publicly available datasets
- A description of any restrictions on data availability
- For clinical datasets or third party data, please ensure that the statement adheres to our policy

LAM-HTGTS sequencing data are deposited at the Gene Expression Omnibus with the accession number GSE192459. The minimal single-molecule dataset is provided as source data for the manuscript. Single-molecule time-traces are available upon request.

Human research participants

Policy information about studies involving human research participants and Sex and Gender in Research.

Reporting on sex and gender	N/A
Population characteristics	N/A
Recruitment	N/A
Ethics oversight	N/A

Note that full information on the approval of the study protocol must also be provided in the manuscript.

Field-specific reporting

Please select the one below that is the best fit for your research. If you are not sure, read the appropriate sections before making your selection.

☒ Life sciences ☐ Behavioural & social sciences ☐ Ecological, evolutionary & environmental sciences

For a reference copy of the document with all sections, see nature.com/documents/nr-reporting-summary-flat.pdf

Life sciences study design

All studies must disclose on these points even when the disclosure is negative.

Sample size	To ensure the construction of robust statistical models that adhere to the requirements of exponential decay curves and normal distributions, this study utilized a minimum of 50 intact DNA molecules for each condition in the single-molecule experiments. Additionally, no less than 30 samples per condition were employed for Sanger sequencing. In the case of LAM-HTGTS, approximately 10 million cells were used for each condition to capture the complete diversity of DSB patterns.
Data exclusions	dCas9 complexes were found to form only partial R-loops in approximately 15% of cases and these immature complexes were excluded from analysis (Fig. 6).
Replication	Single-molecule experiments were repeated a minimum of three times for each condition. Biochemical assays for the primary data were performed in triplicate, while supplementary data experiments were repeated at least twice for each condition. The LAM-HTGTS data underwent a minimum of two replications. All replication attempts were successful.
Randomization	The experiments were conducted in a batch manner to compare the effects of different guide RNAs in the single-molecule and biochemical assays. Each batch included all the guide RNAs to minimize the potential impact of other factors, such as variations in the quality of Cas9 and buffer composition. The same strategy was applied to the sample preparation for LAM-HTGTS to ensure consistency and control for external factors.
Blinding	The investigators in this study made efforts to minimize bias by implementing blinding procedures. Specifically, for the single-molecule assay, data collection was conducted using single-molecule microscopy, ensuring unbiased observation. In the case of Sanger sequencing and Illumina sequencing, all samples were labeled with condition-irrelevant names, and data collection was carried out by third parties. Data analysis for all experiments was performed using scripts or software with consistent settings. Biochemical assays were conducted in batch mode, and data were collected using Chemidoc and analyzed using ImageJ with identical settings to maintain consistency and minimize bias.

Reporting for specific materials, systems and methods

We require information from authors about some types of materials, experimental systems and methods used in many studies. Here, indicate whether each material, system or method listed is relevant to your study. If you are not sure if a list item applies to your research, read the appropriate section before selecting a response.

Materials & experimental systems

n/a	Involved in the study	
☐	☒	Antibodies
☐	☒	Eukaryotic cell lines
☒	☐	Palaeontology and archaeology
☒	☐	Animals and other organisms
☒	☐	Clinical data
☒	☐	Dual use research of concern

Methods

n/a	Involved in the study	
☒	☐	ChIP-seq
☐	☒	Flow cytometry
☒	☐	MRI-based neuroimaging

Antibodies

Antibodies used	Rabbit anti-Flag (Cell Signalling Technology #14793S, 1:2000, 10ml final); Mouse anti-Actin-HRP (clone 4, Santa Cruz # sc-47778, 1:4000, 10ml final); Goat anti-Rabbit IgG-HRP (Thermo Scientific # G-21234, 1:2000, 10ml final)
Validation	Primary antibodies are well-validated for western blotting as per manufacturer recommendations (Anti-Flag has been used for western blotting in over 270 publications; mouse anti-actin-HRP has been cited in over 13,000 publications).

Eukaryotic cell lines

Policy information about cell lines and Sex and Gender in Research

Cell line source(s)	HEK293T and K562 were from ATCC. K562-iCas9-GFP was generated by transfecting K562 with lentivirus.
Authentication	STR(short tandem repeat)-based PCR plus genotyping
Mycoplasma contamination	Mycoplasma contamination was not detected by MycoAlert® Mycoplasma Detection Kit (Lonza #LT07-118).
Commonly misidentified lines (See ICLAC register)	No commonly misidentified cell lines were used in this study.

Flow Cytometry

Plots

Confirm that:

☒ The axis labels state the marker and fluorochrome used (e.g. CD4-FITC).

☒ The axis scales are clearly visible. Include numbers along axes only for bottom left plot of group (a 'group' is an analysis of identical markers).

☒ All plots are contour plots with outliers or pseudocolor plots.

☒ A numerical value for number of cells or percentage (with statistics) is provided.

Methodology

Sample preparation	K562-iCas9-GFP cells were nucleofected with pMCB320-sgRNA-mCherry (Lonza nucleofector) and subsequently cultured in media containing Doxycycline for 7 days. Afterward, live cells were collected and directly analyzed by flow cytometry.
Instrument	BD LSRFortessa X-20
Software	FlowJo (version 10.8)
Cell population abundance	Cell viability was assessed by Trypan blue staining, and the cell count was determined using the Luna II automated cell counter, which indicated an approximate concentration of 0.5 million cells/ml (majority of cells were viable).
Gating strategy	FSC-A/SSC-A

☒ Tick this box to confirm that a figure exemplifying the gating strategy is provided in the Supplementary Information.
