# Supplementary material for: Shifted PAMs generate DNA overhangs and enhance SpCas9 post-catalytic complex dissociation
Source: Nat Struct Mol Biol. 2023 Oct 12;30(11):1707–18. doi: 10.1038/s41594-023-01104-6 (PMC10643121; doi:10.1038/s41594-023-01104-6)

Single Molecule Time Traces

Figure 2b (Part 1)

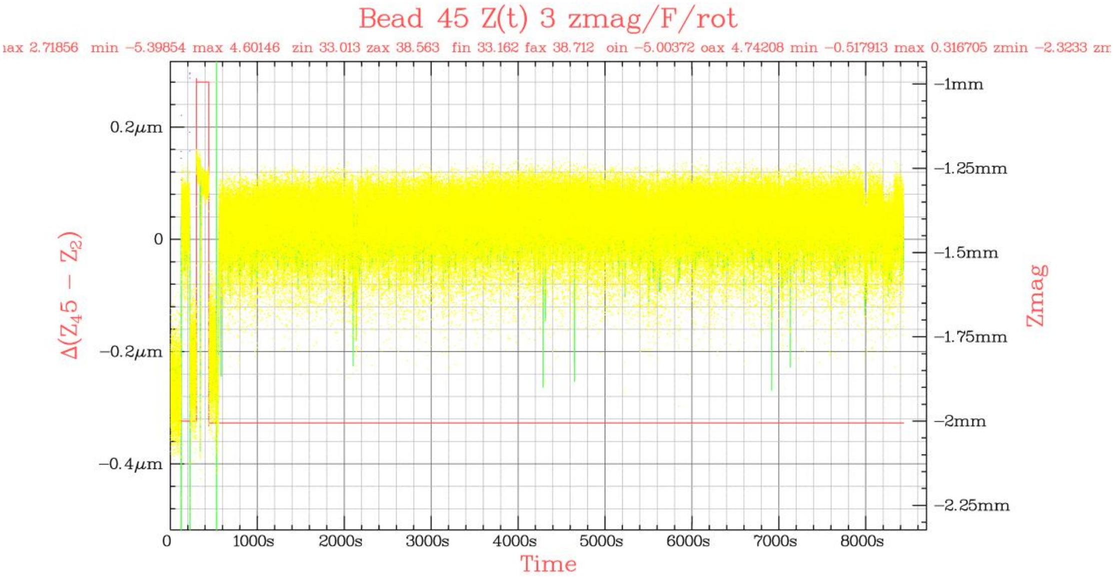

Figure 2b (Part 2)

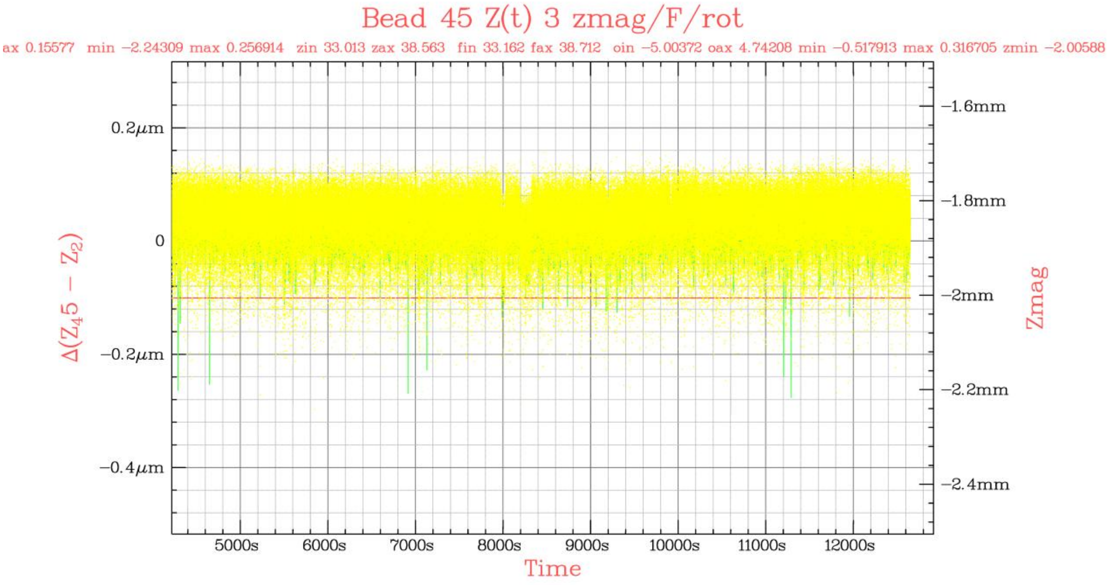

Figure 2e

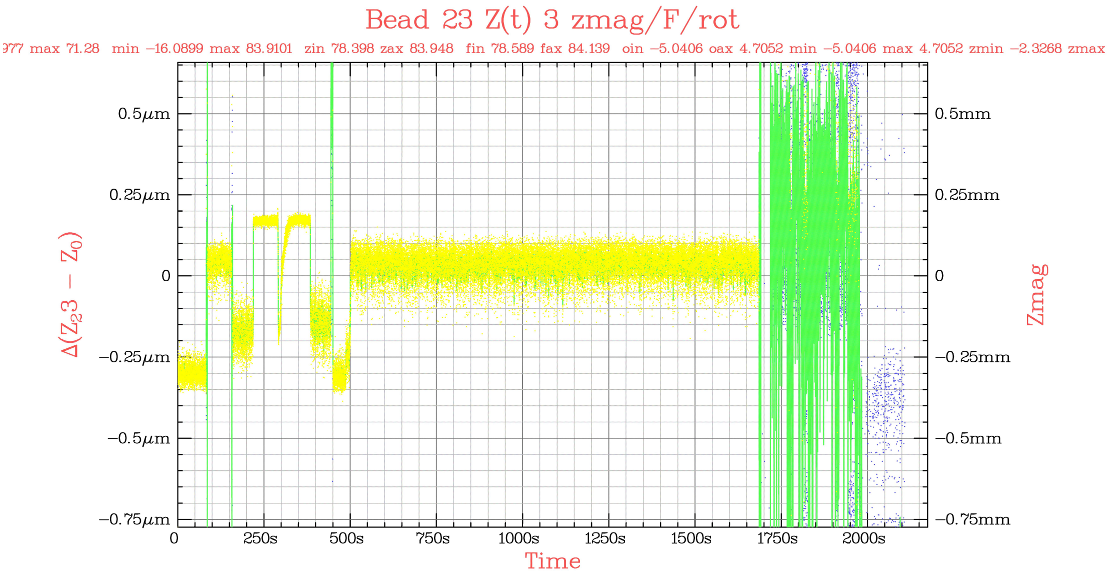

Figure 2h

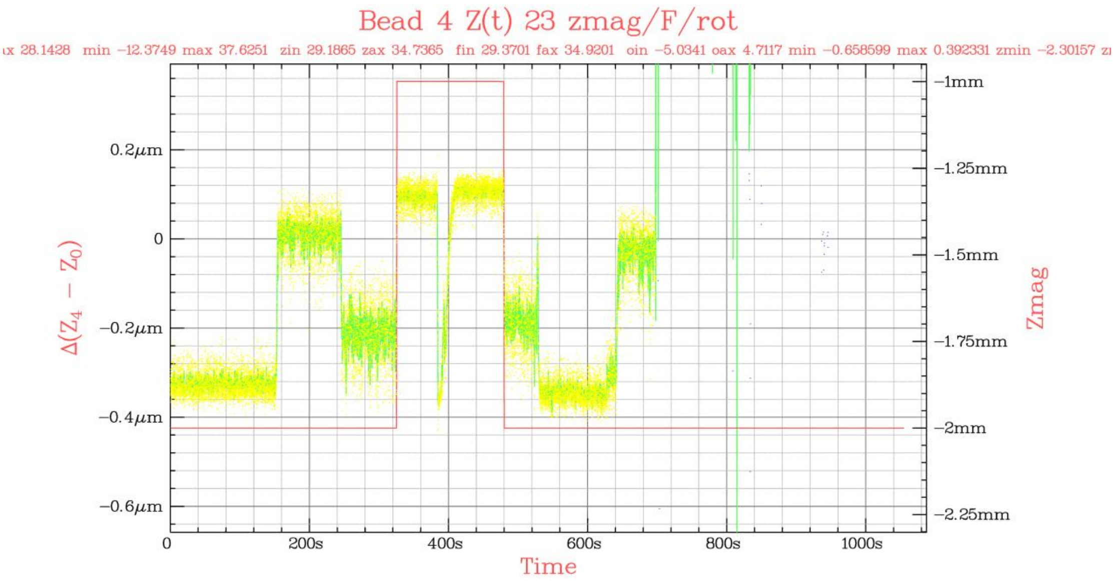

Figure 2k (Part 1)

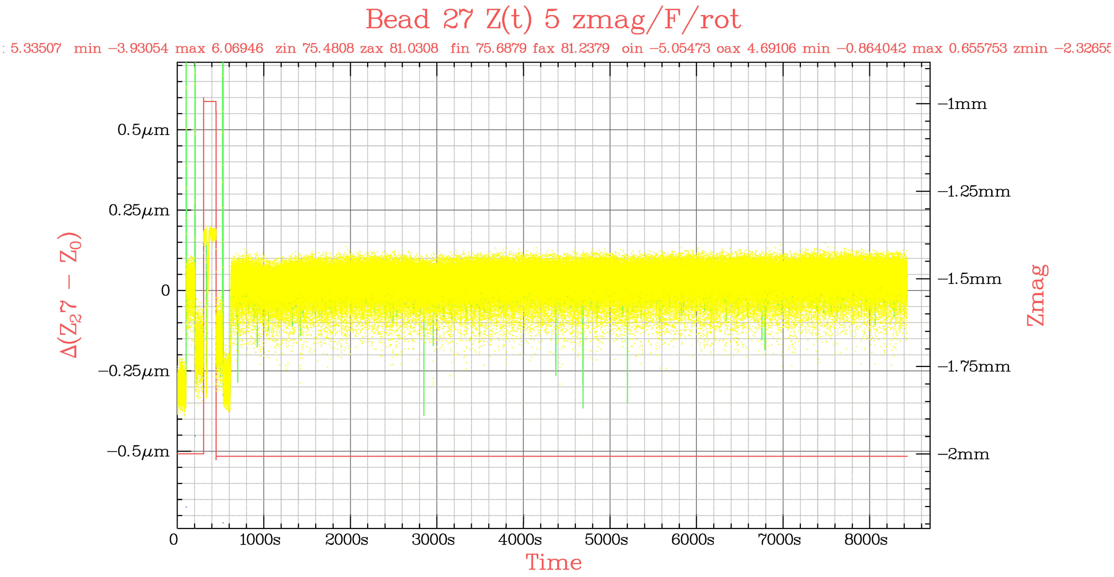

Figure 2k (Part 2)

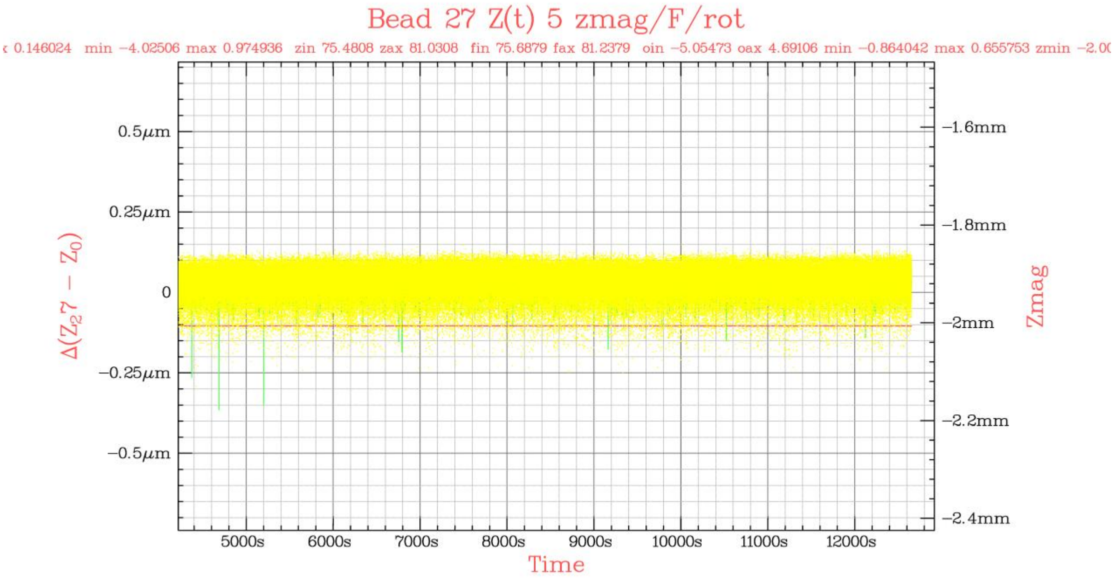

Figure 3a

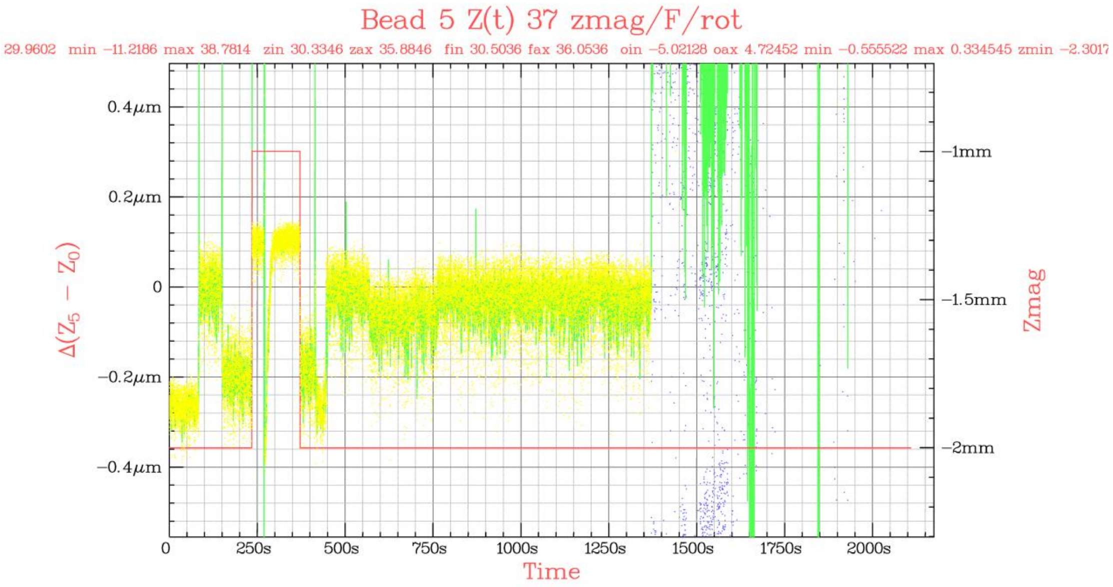

Figure 3c (Part 1)

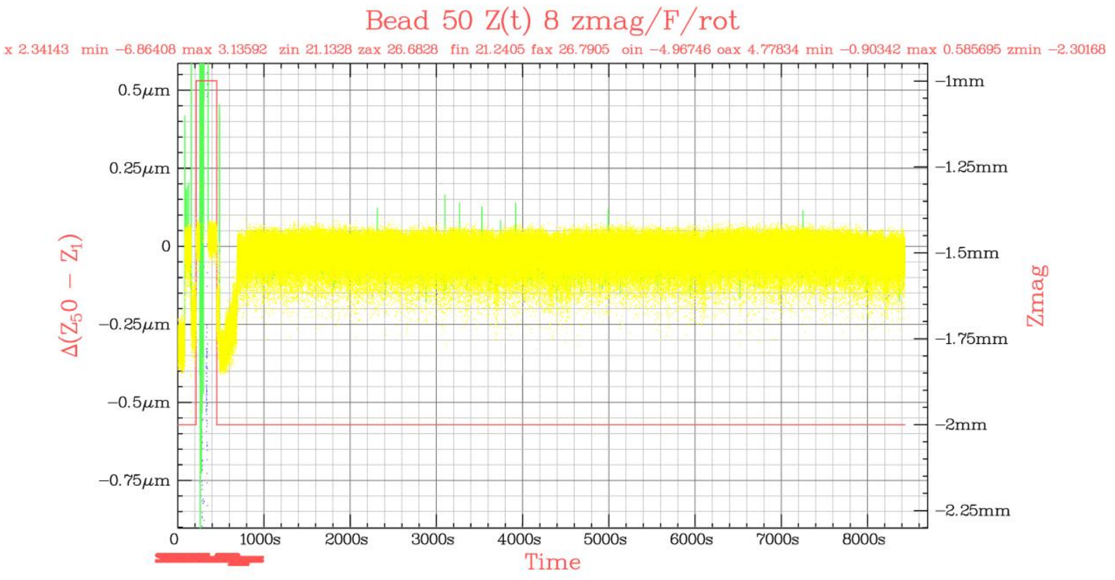

Figure 3c (Part 2)

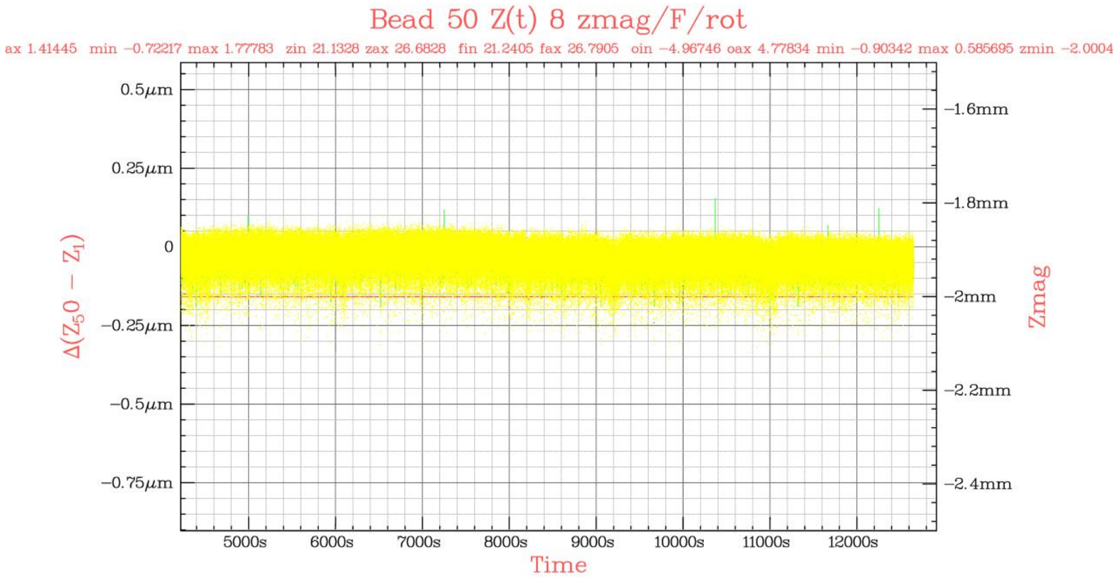

Figure 3e

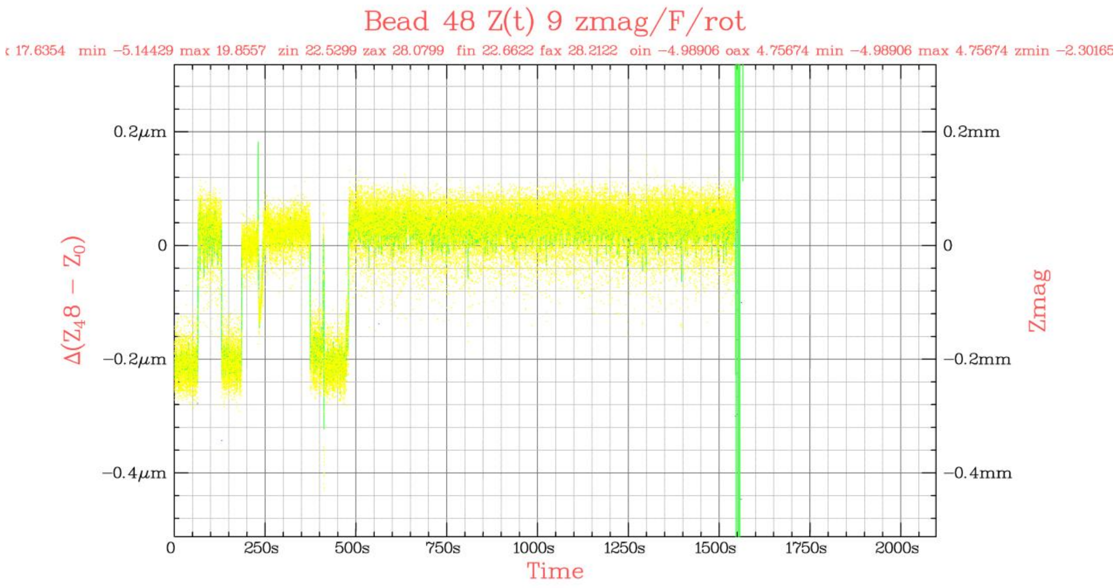

Figure 4a

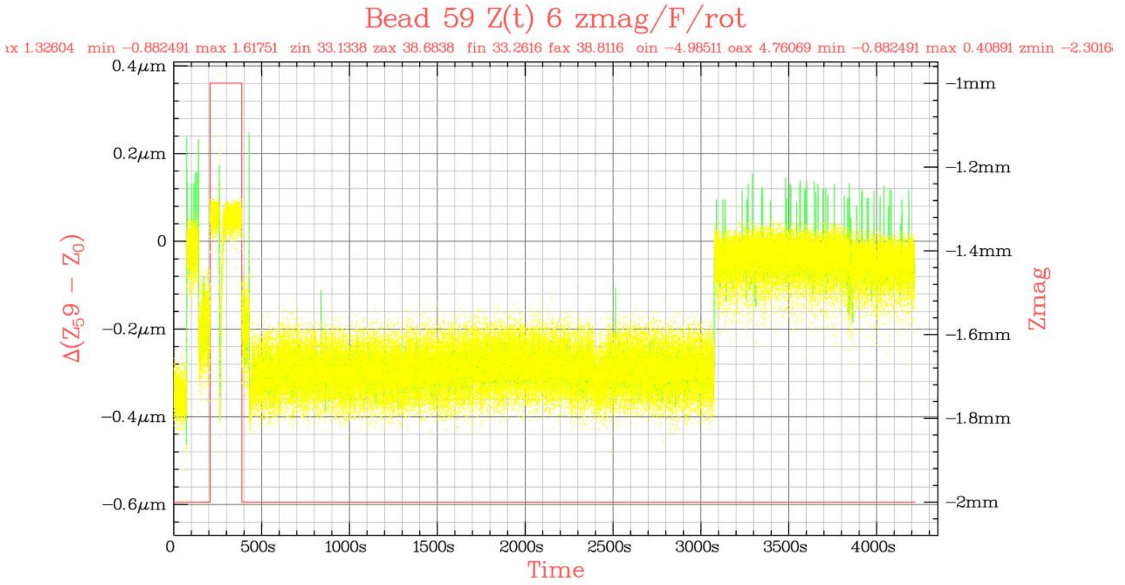

Figure 4d

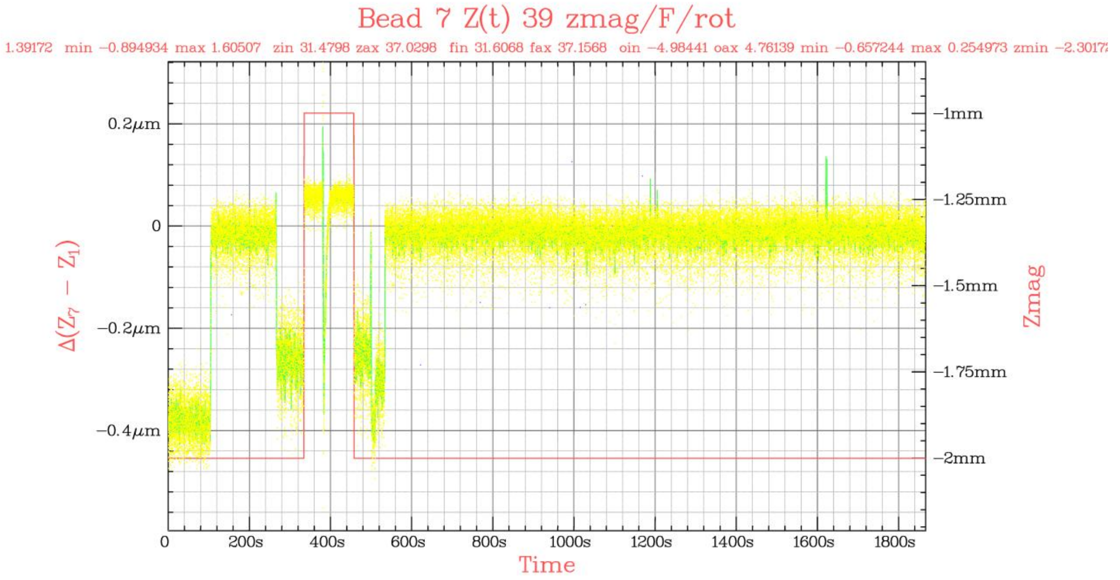

Figure 4g

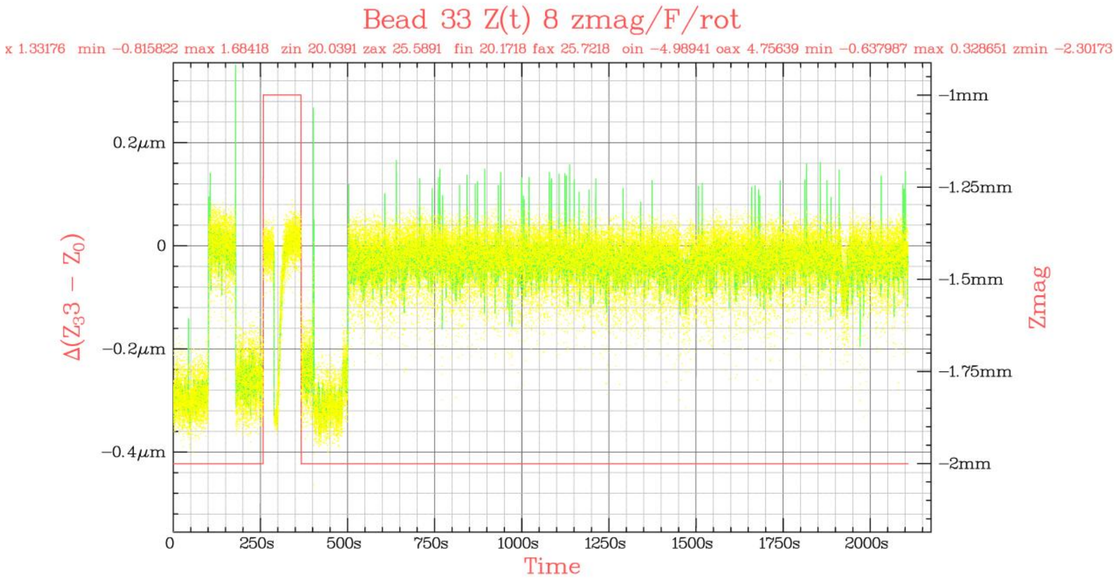

Figure 4j

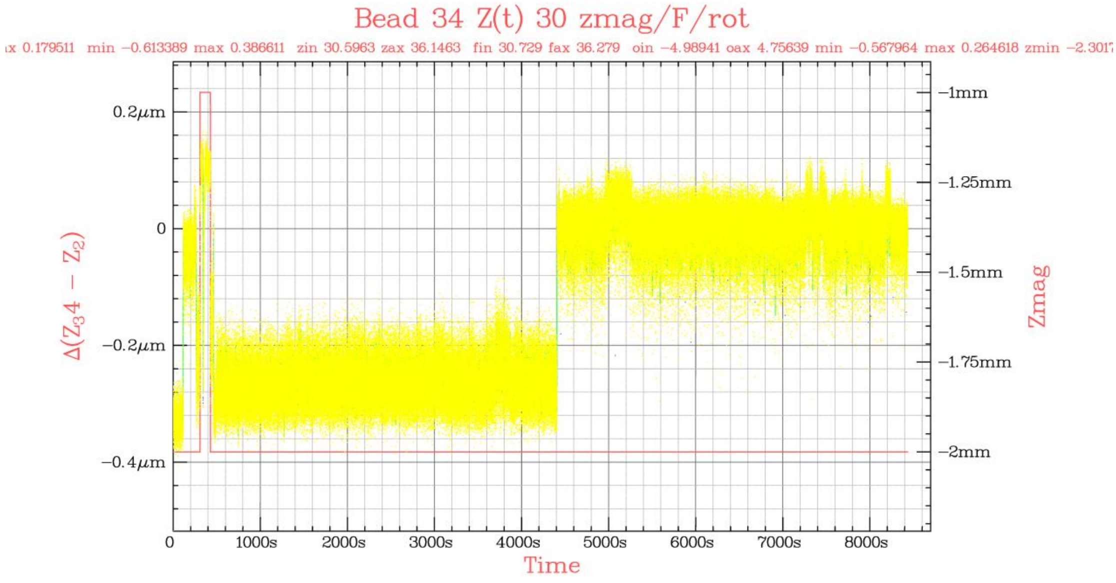

Figure 5a

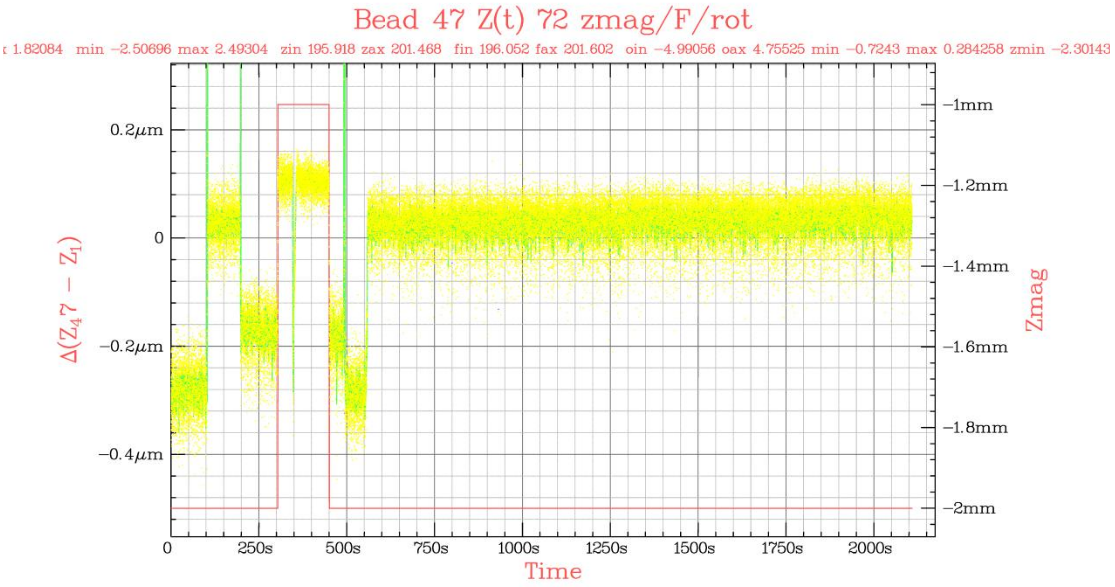

Figure 5d

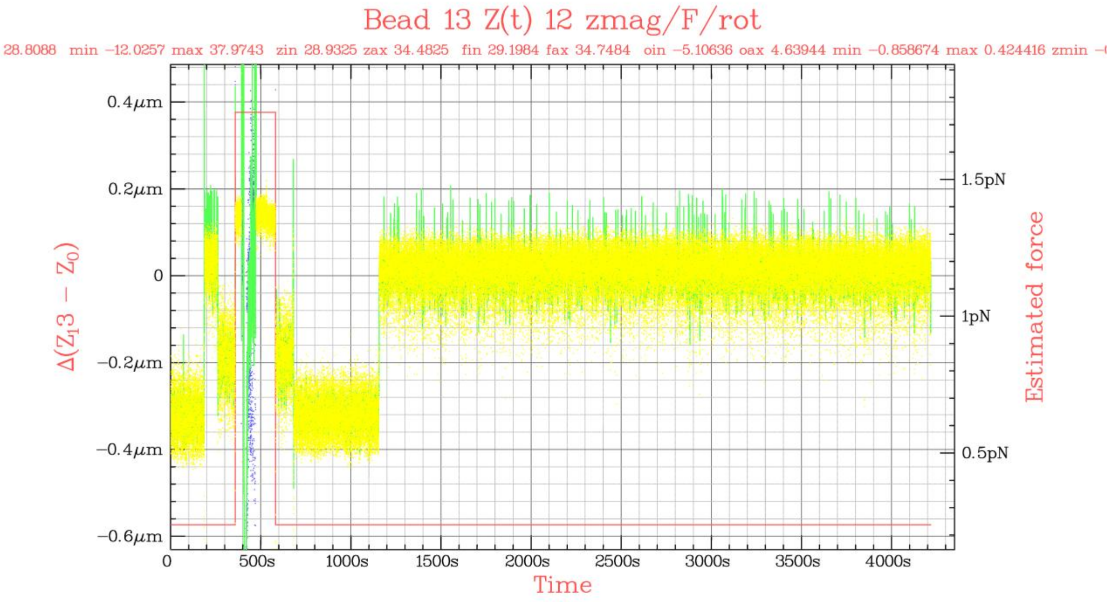

Figure 5g (Part 1)

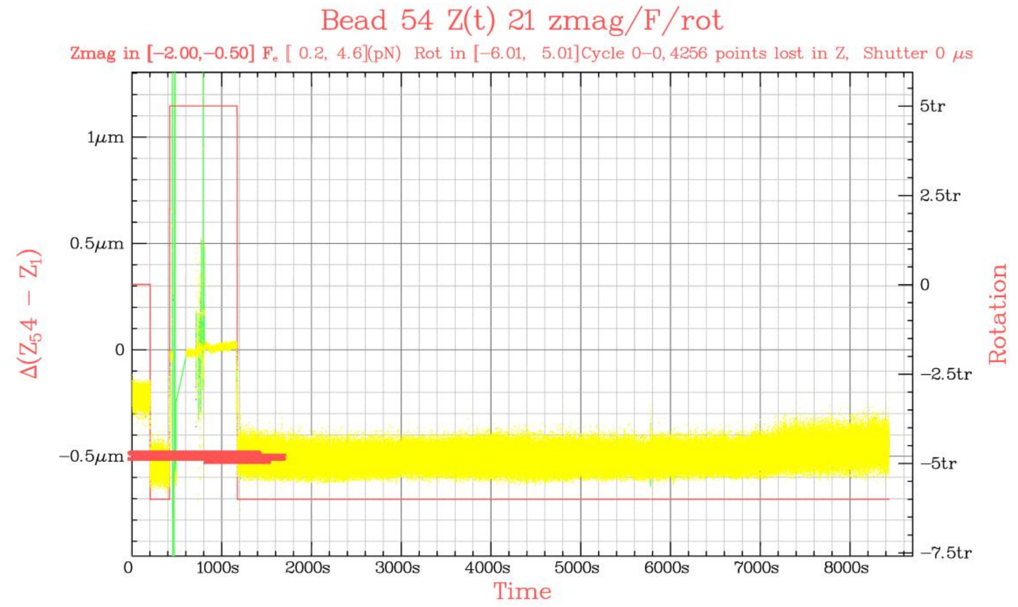

Figure 5g (Part 2)

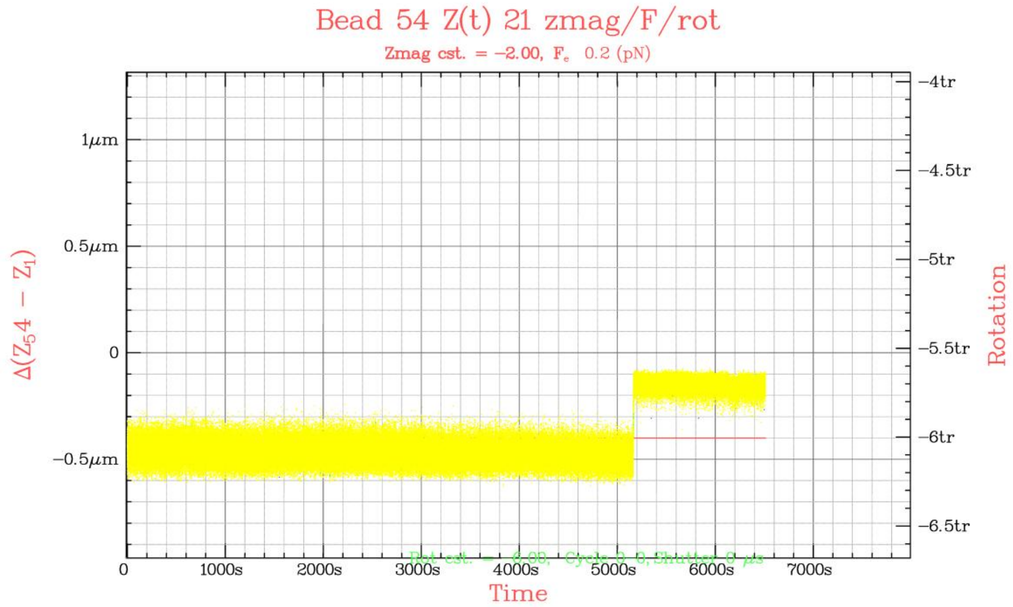

Figure 5l

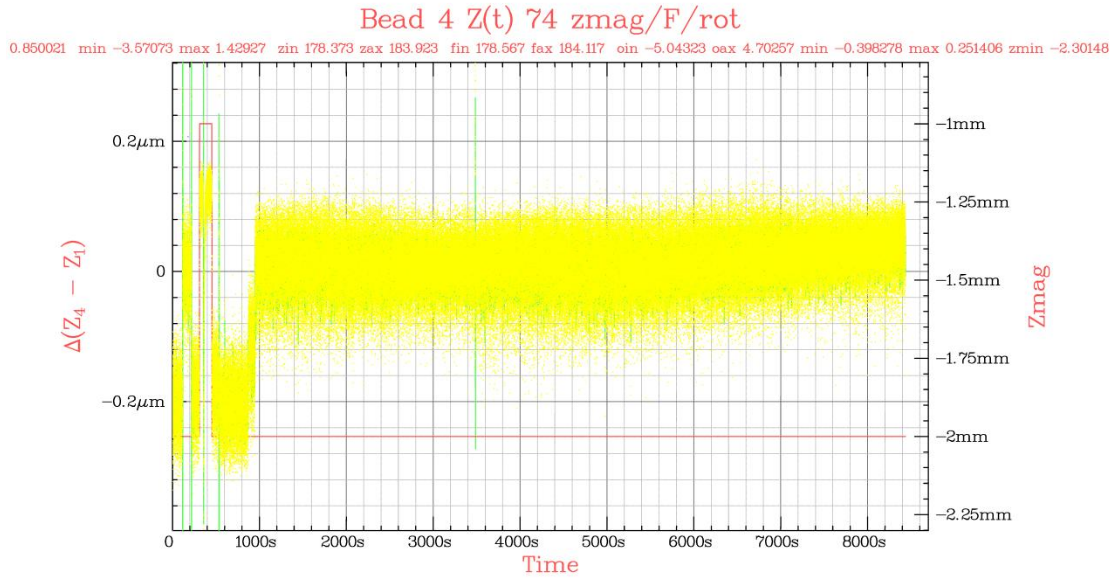

Bead 4 Z(t) 74 zmag/F/rot
0.850021 min −3.57073 max 1.42927 zin 178.373 zax 183.923 fin 178.567 fax 184.117 oin −5.04323 oax 4.70257 min −0.398278 max 0.251406 zmin −2.30148
0.2μm
0
−0.2μm
Δ(Z4 − Z1)
−1mm
−1.25mm
−1.5mm
−1.75mm
−2mm
−2.25mm
Zmag
0
1000s
2000s
3000s
4000s
5000s
6000s
7000s
8000s
Time

Extended Data Fig. 7a

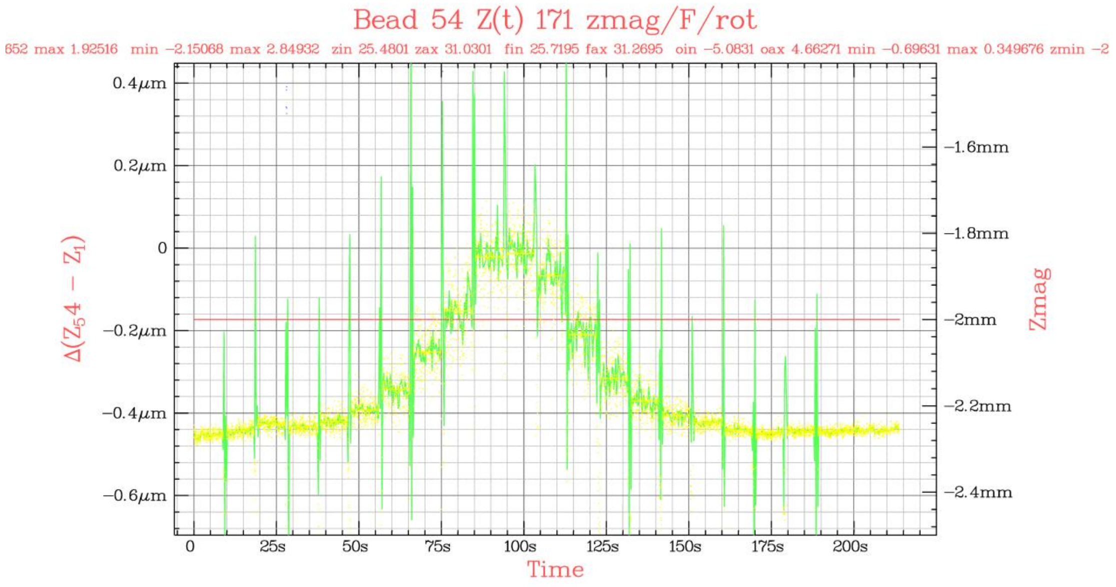

Extended Data Fig. 7b

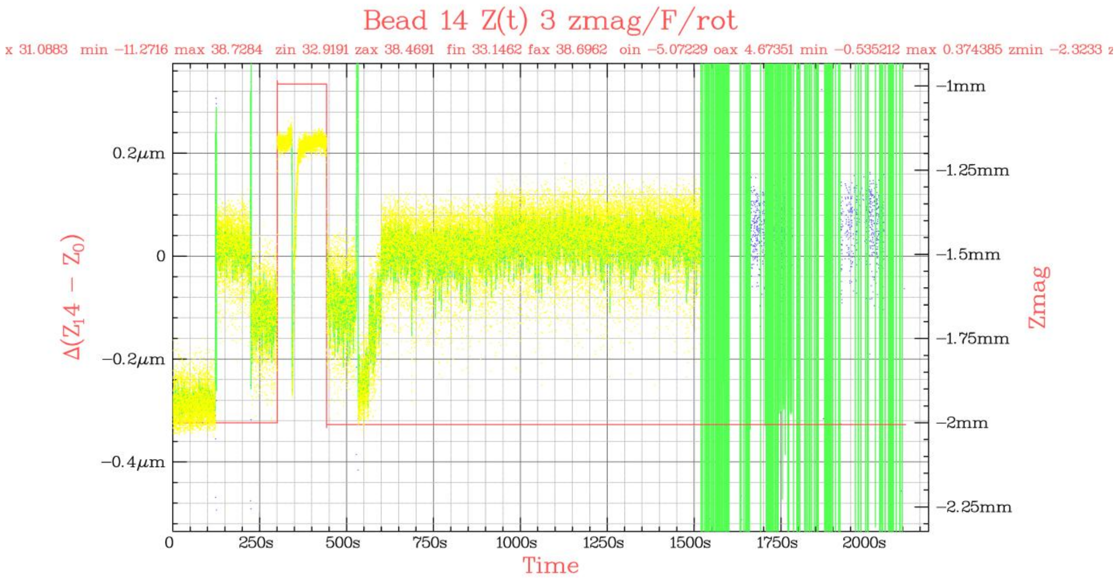

Extended Data Fig. 7c

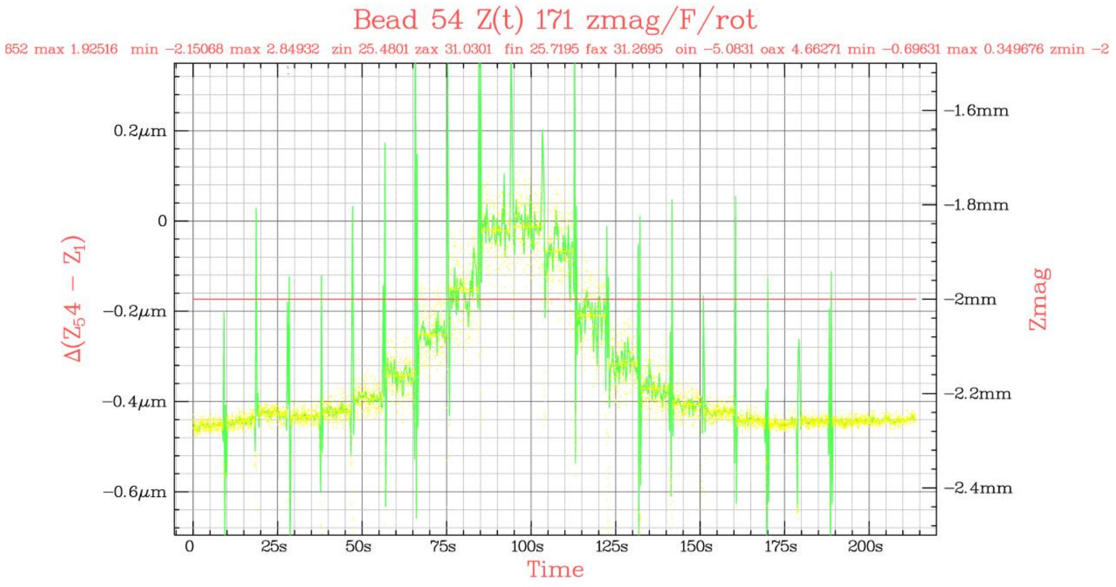

Extended Data Fig. 7d

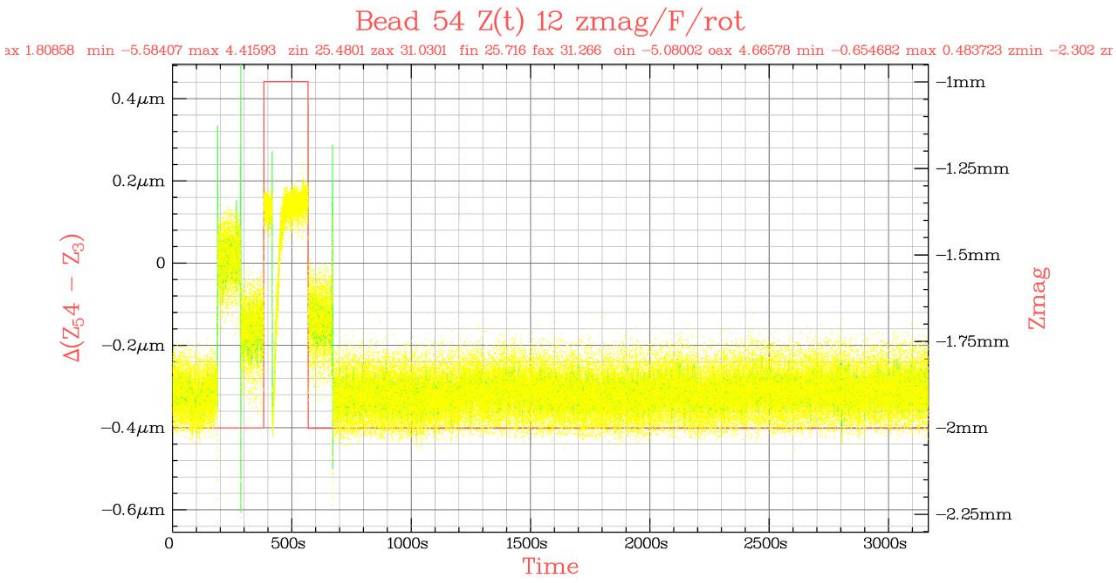

Supplement: Supplementary file 4 — Single molecule time traces. [file 41594_2023_1104_MOESM4_ESM.pdf]
