# Supplementary material for: Shifted PAMs generate DNA overhangs and enhance SpCas9 post-catalytic complex dissociation
Source: Nat Struct Mol Biol. 2023 Oct 12;30(11):1707–18. doi: 10.1038/s41594-023-01104-6 (PMC10643121; doi:10.1038/s41594-023-01104-6)

Unprocessed Gels and Blots

Note: For independent replicate (Rep) figures, the Rep1 is the representative figure shown in the manuscript by default.

Figure 1b, 1d (Rep1)

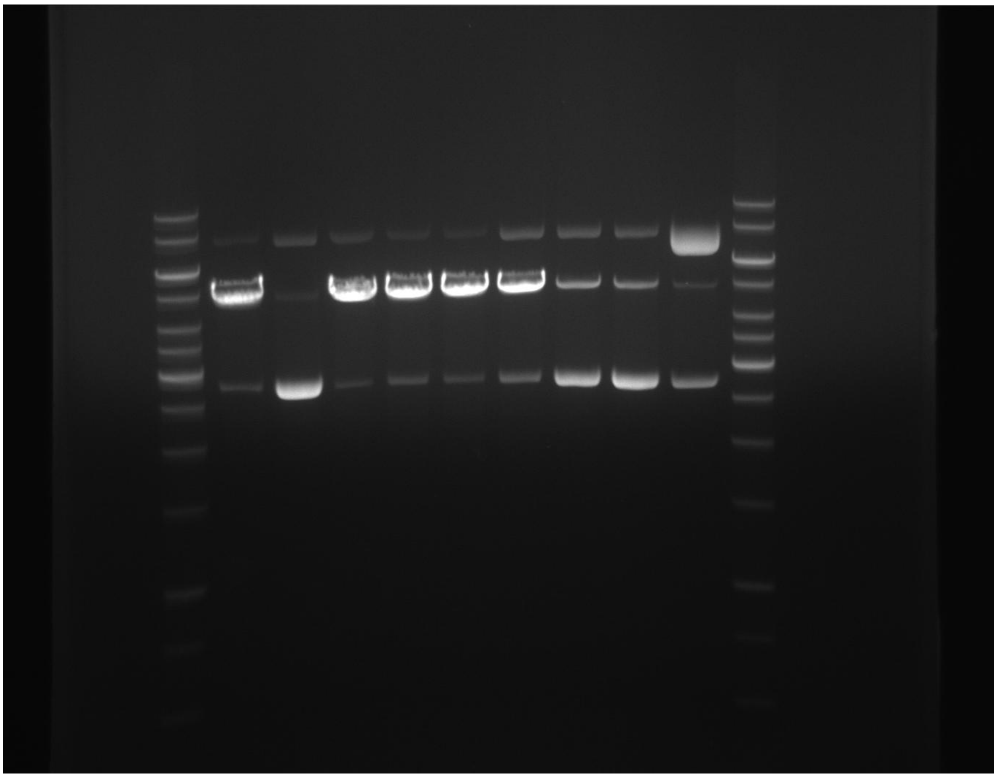

Figure 1b, 1d (Rep2)

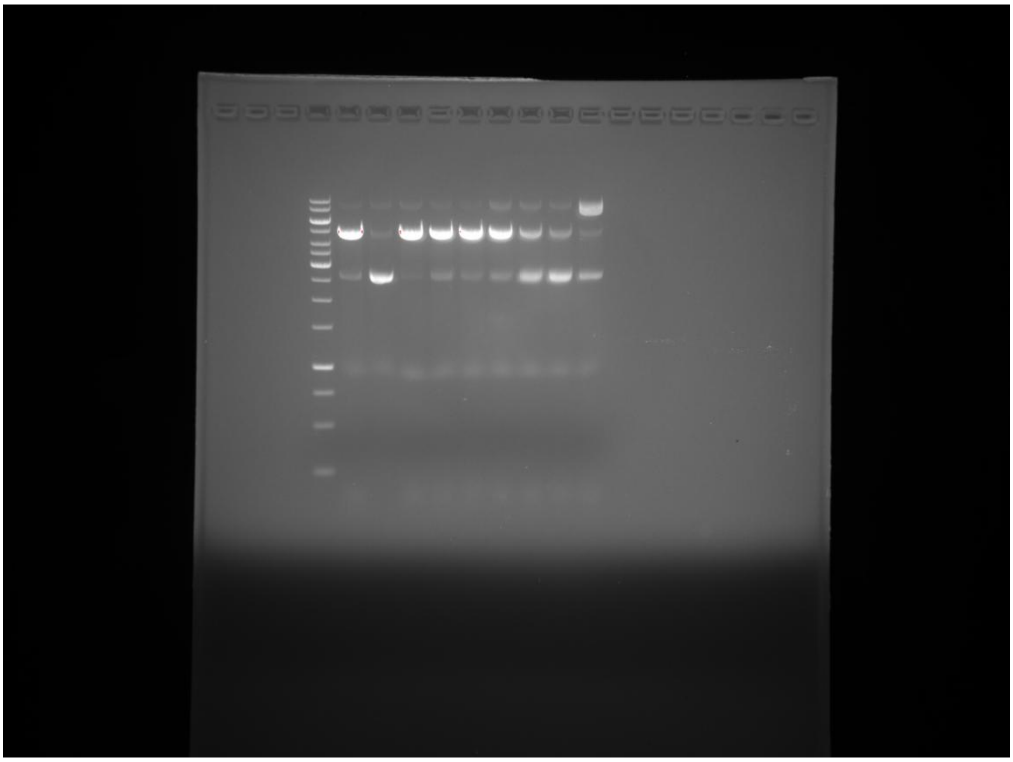

Figure 1b, 1d (Rep3)

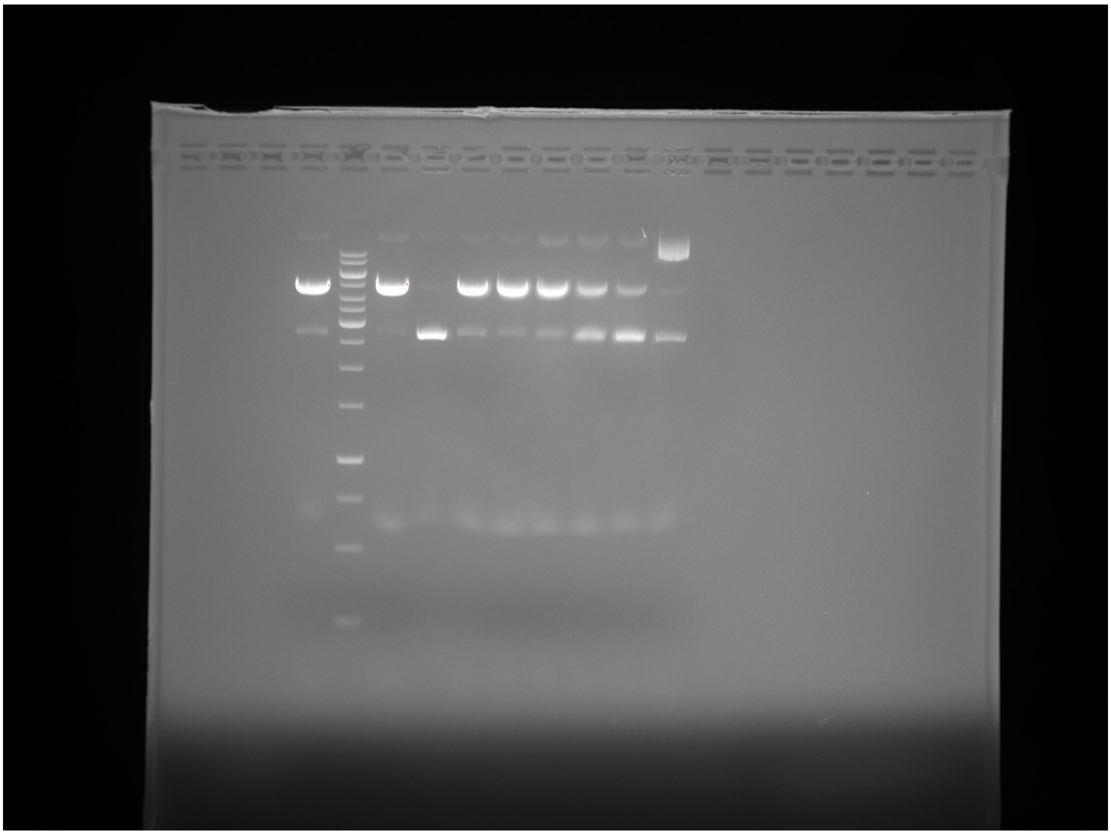

Figure 1c, 1d (Rep1)

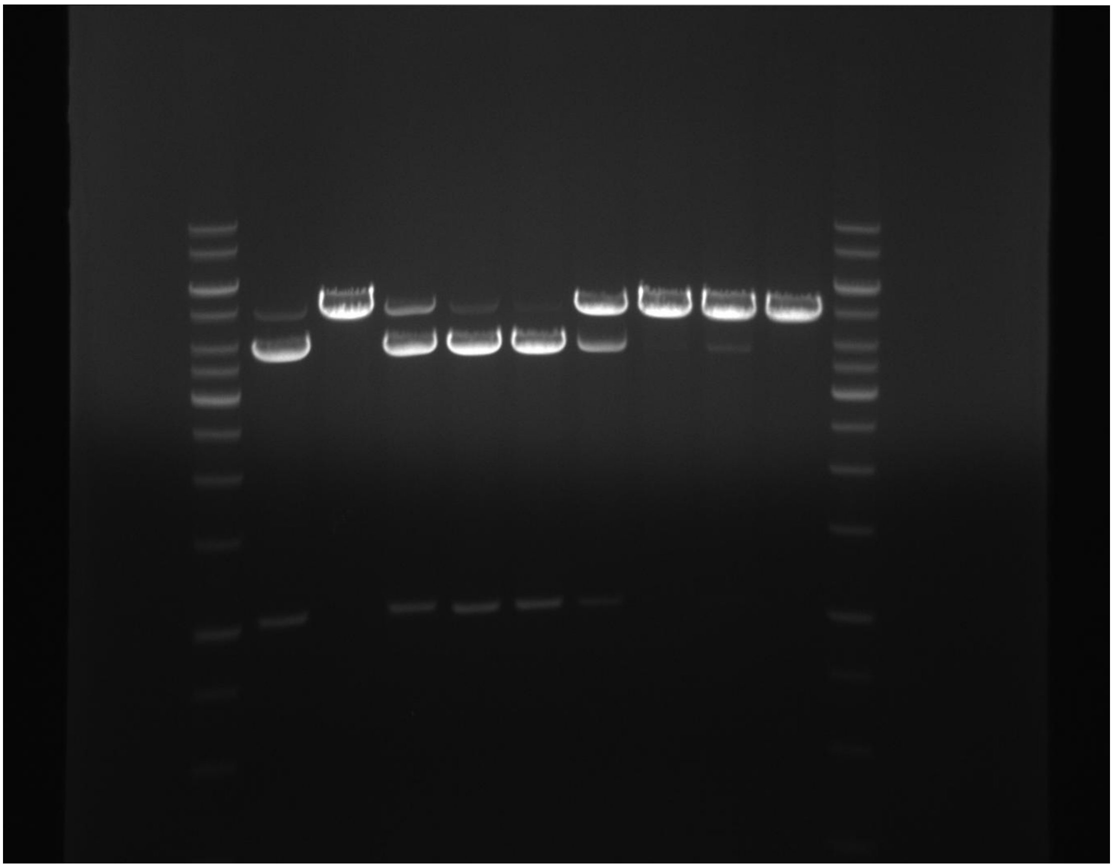

Figure 1c, 1d (Rep2)

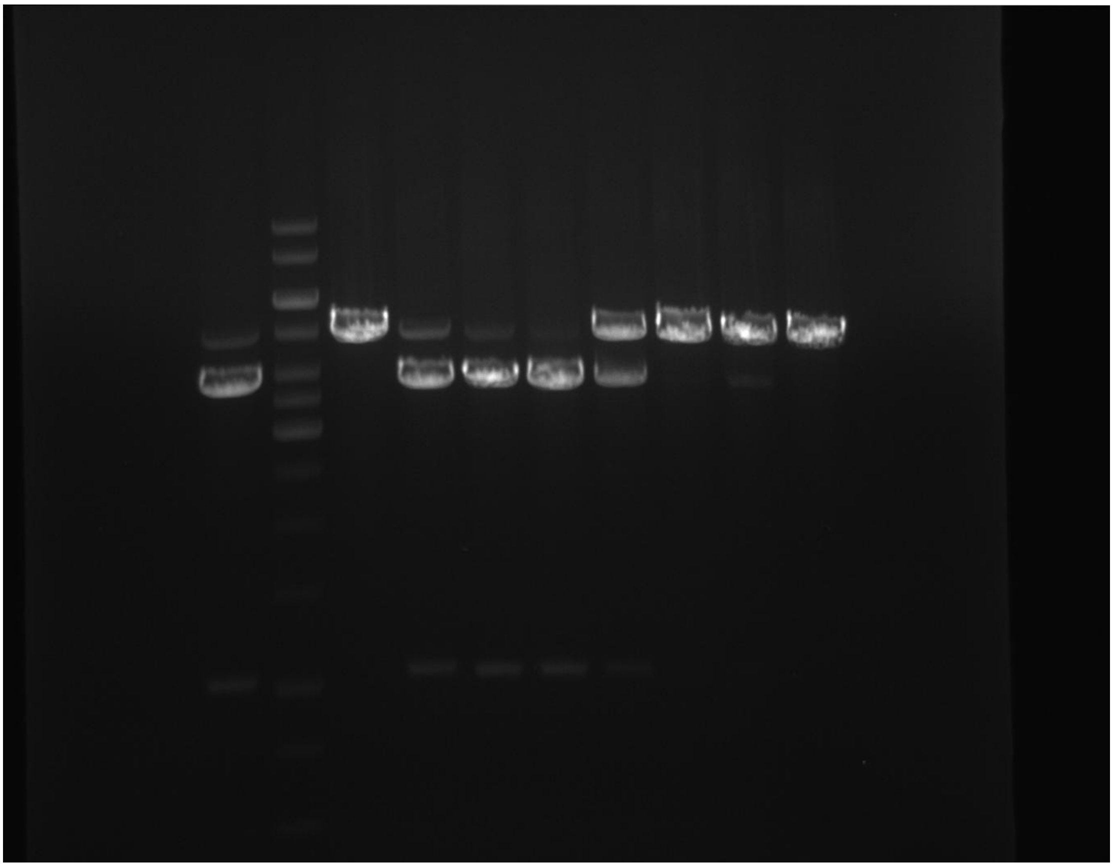

Figure 1c, 1d (Rep3)

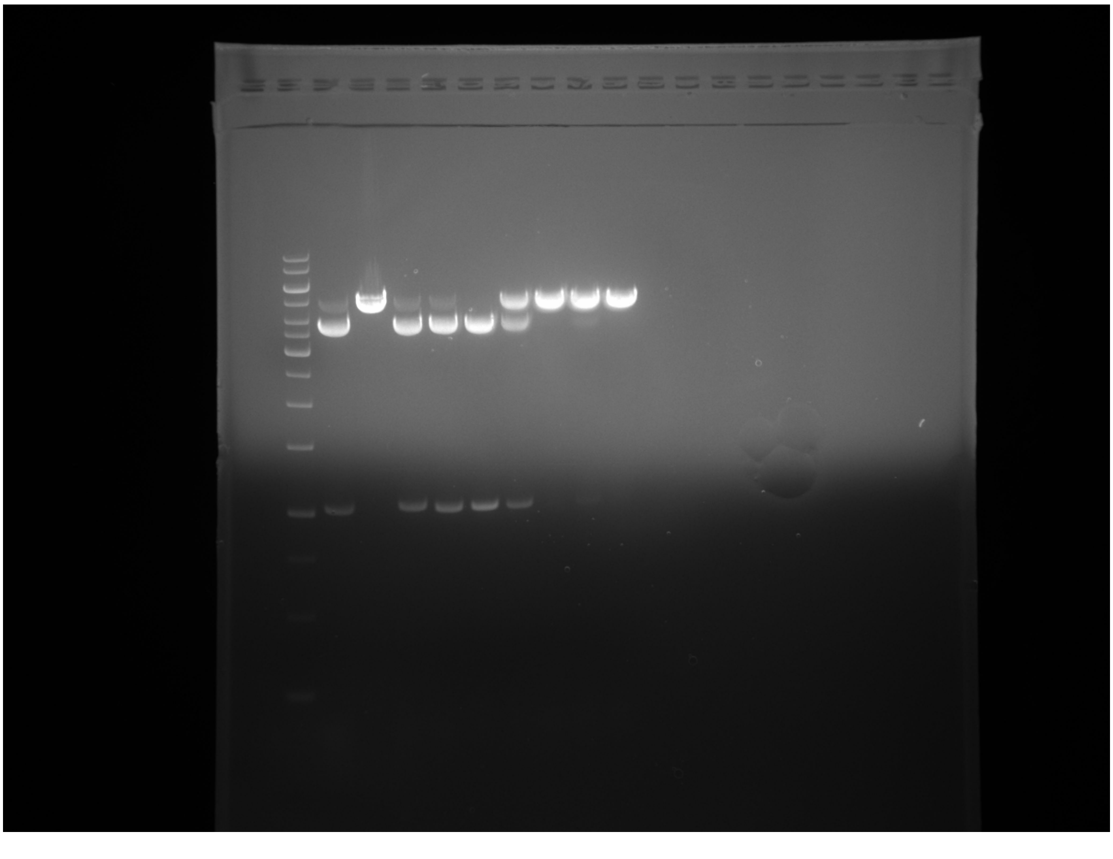

Supplement: Supplementary file 7 — Unprocessed gels and blots. [file 41594_2023_1104_MOESM7_ESM.pdf]
