# Supplementary material for: Shifted PAMs generate DNA overhangs and enhance SpCas9 post-catalytic complex dissociation
Source: Nat Struct Mol Biol. 2023 Oct 12;30(11):1707–18. doi: 10.1038/s41594-023-01104-6 (PMC10643121; doi:10.1038/s41594-023-01104-6)
Supplement: Supplementary file 8 — Unprocessed gels and blots. [file 41594_2023_1104_MOESM8_ESM.pdf]

Extended Data Fig. 1b, 1d (Rep1)

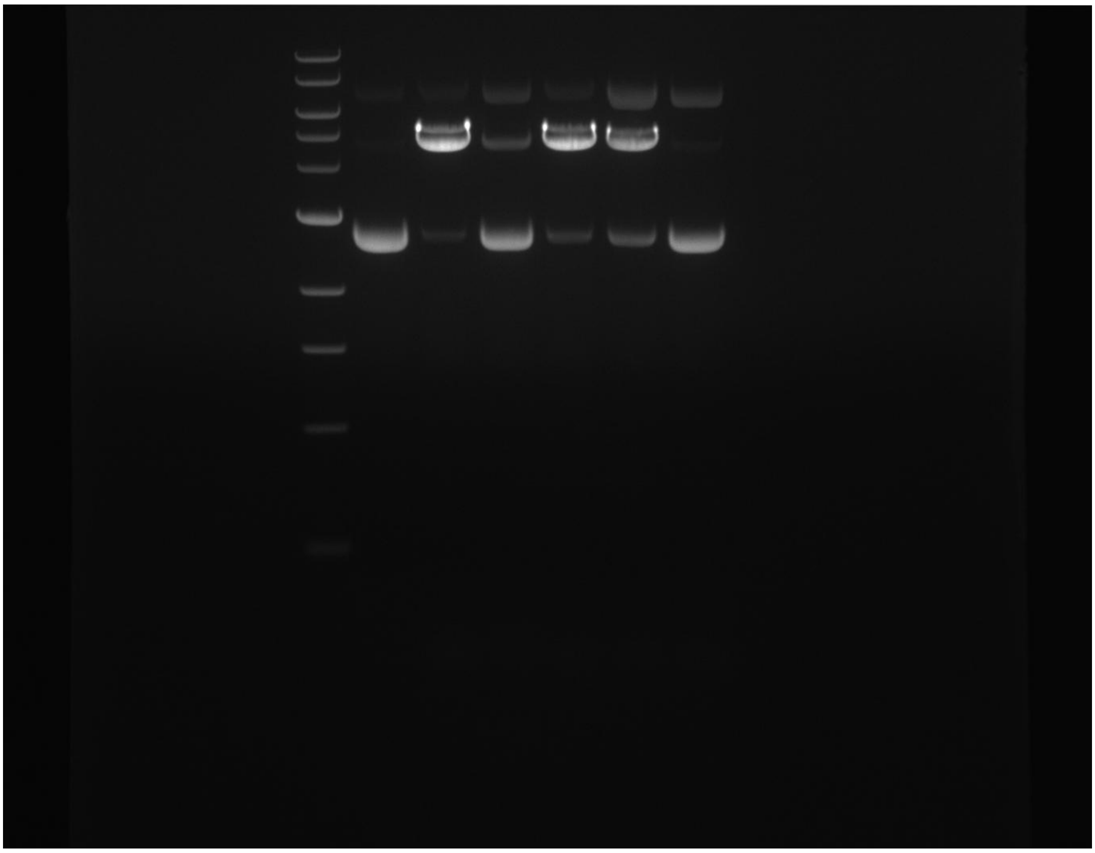

Extended Data Fig. 1b, 1d (Rep2)

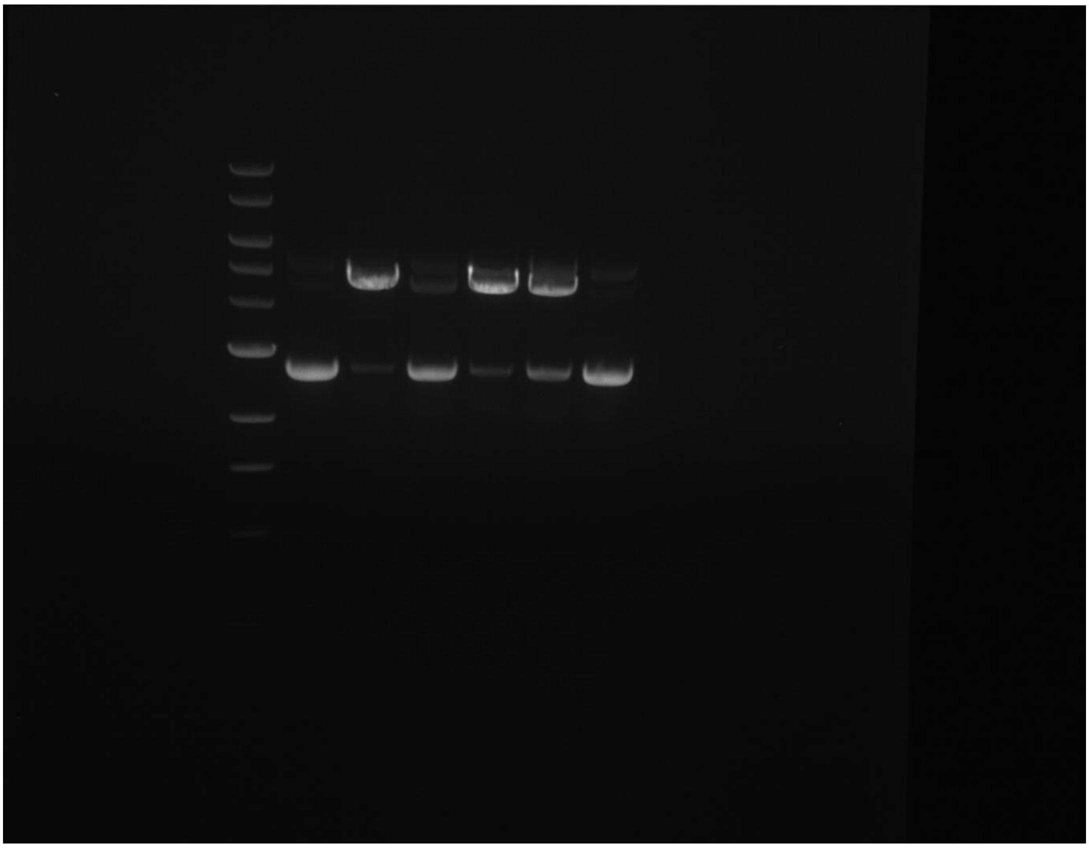

Extended Data Fig. 1b, 1d (Rep3)

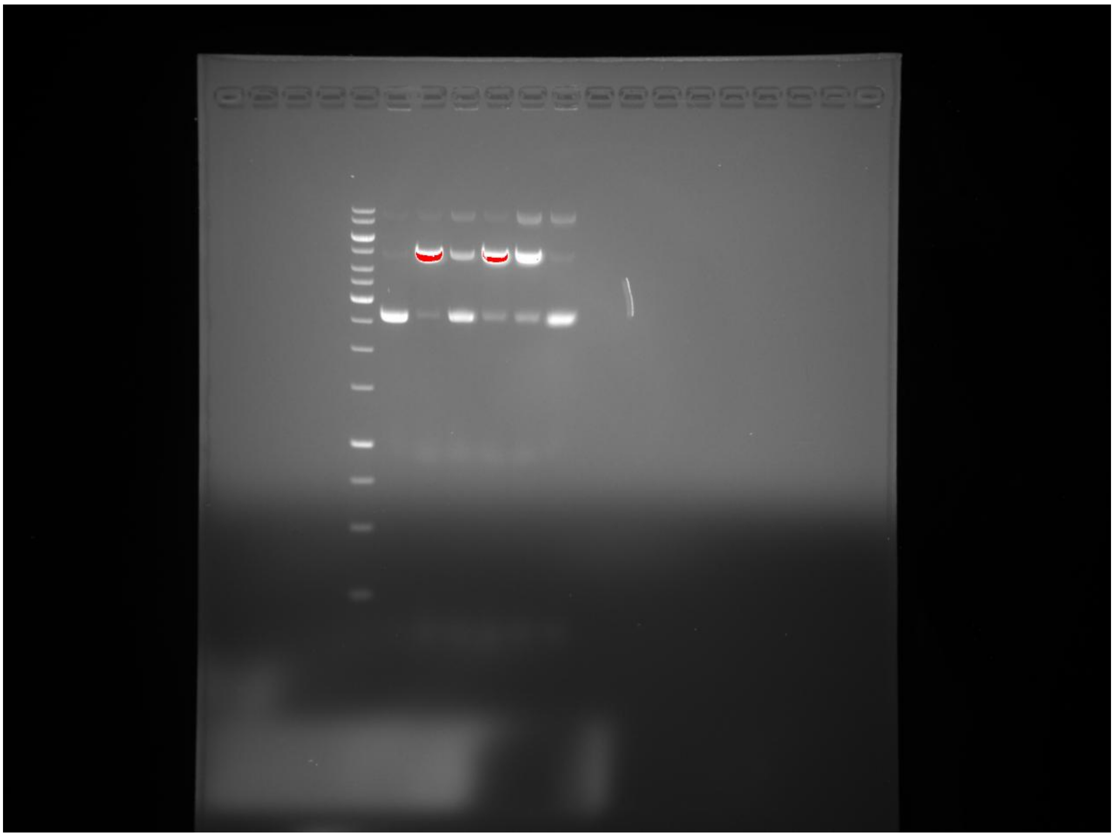

Extended Data Fig. 1c, 1d (Rep1)

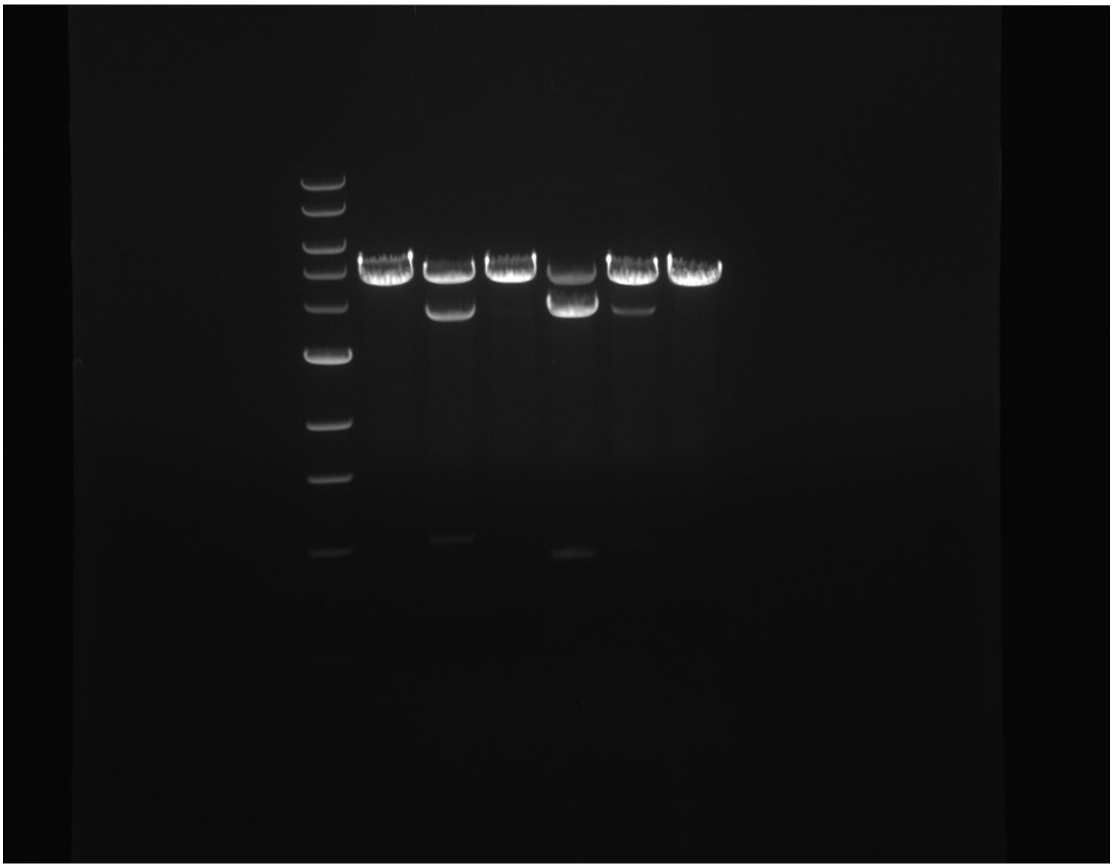

Extended Data Fig. 1c, 1d (Rep2)

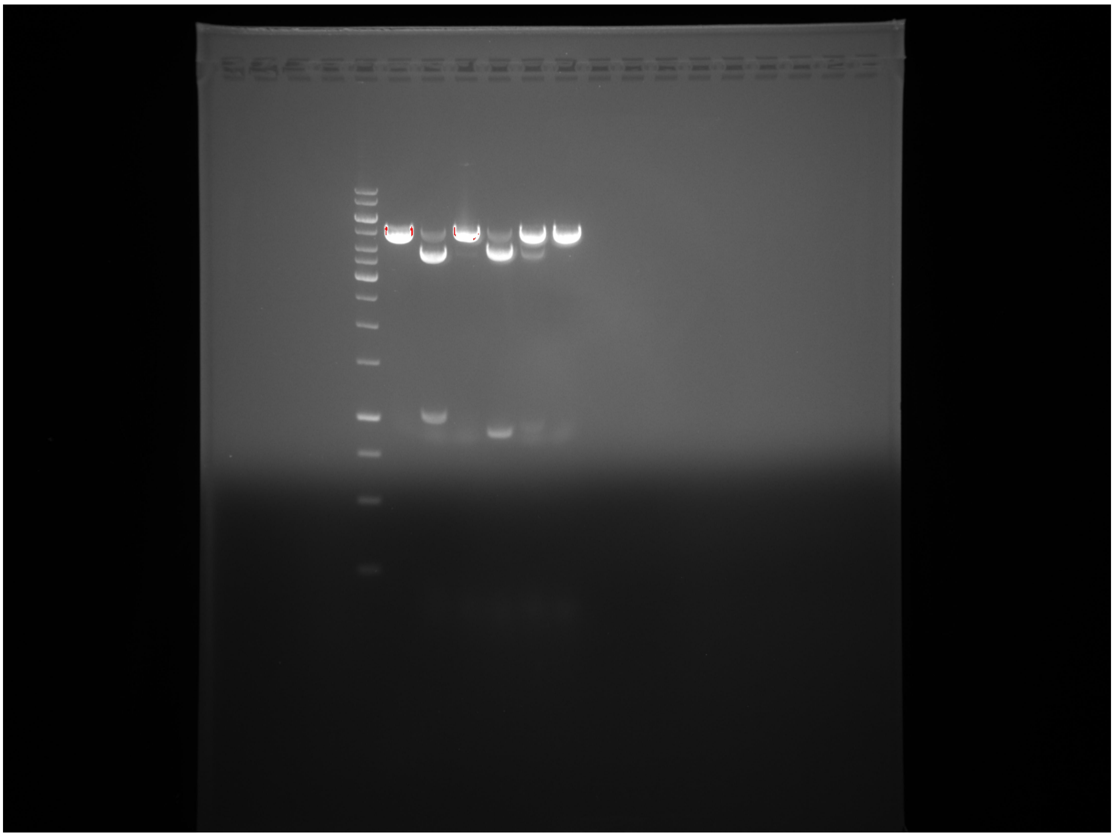

Extended Data Fig. 1c, 1d (Rep3)

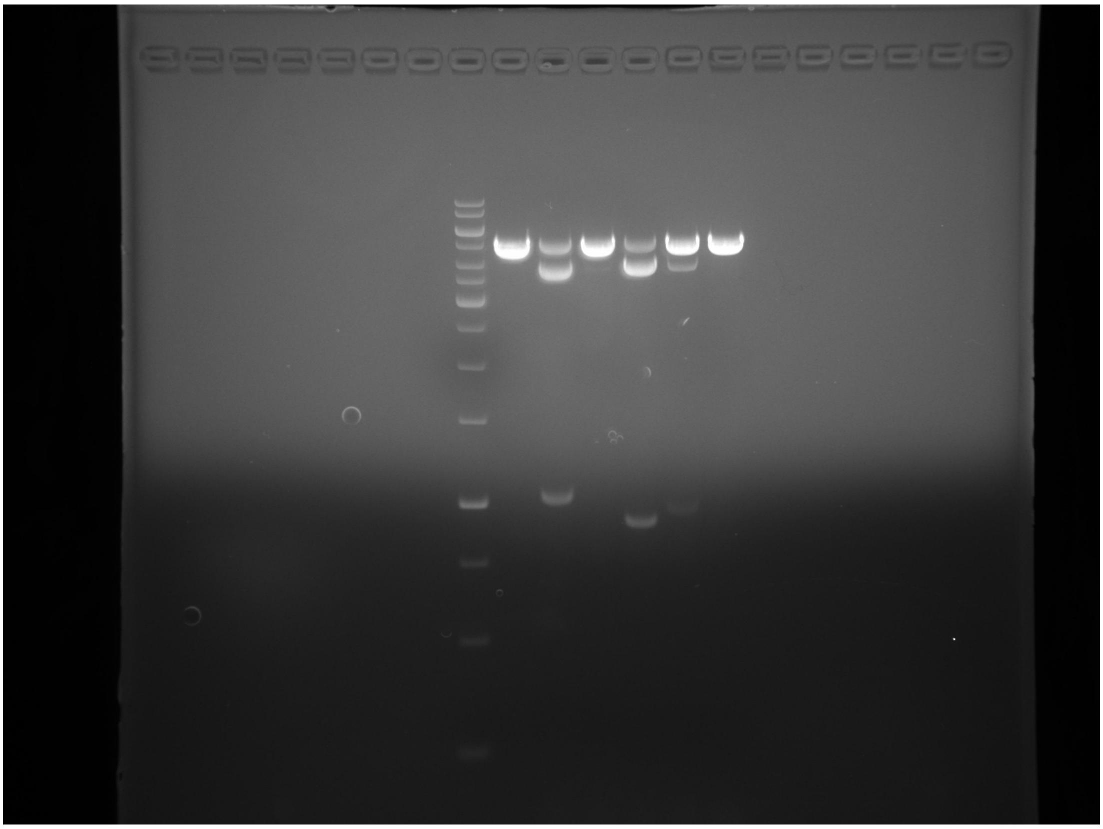

Extended Data Fig. 1c, 1d (Rep4)

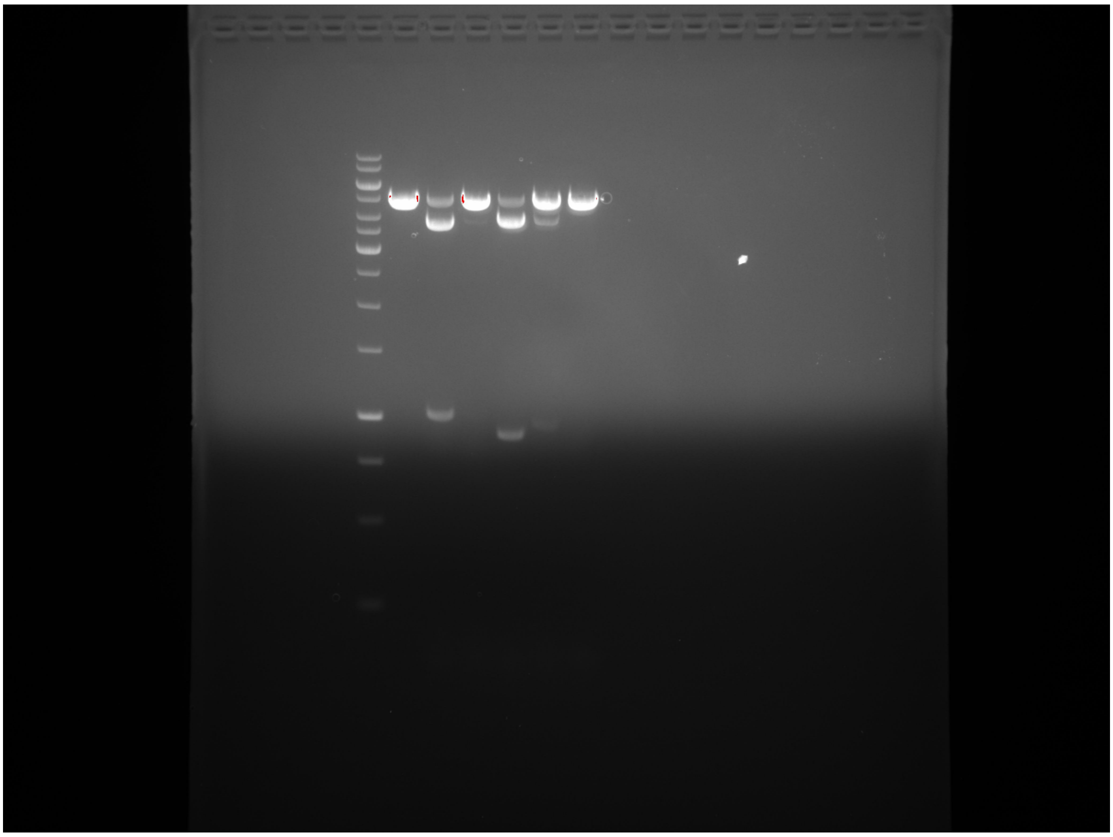

Extended Data Fig. 1e (5 mins, Rep1)

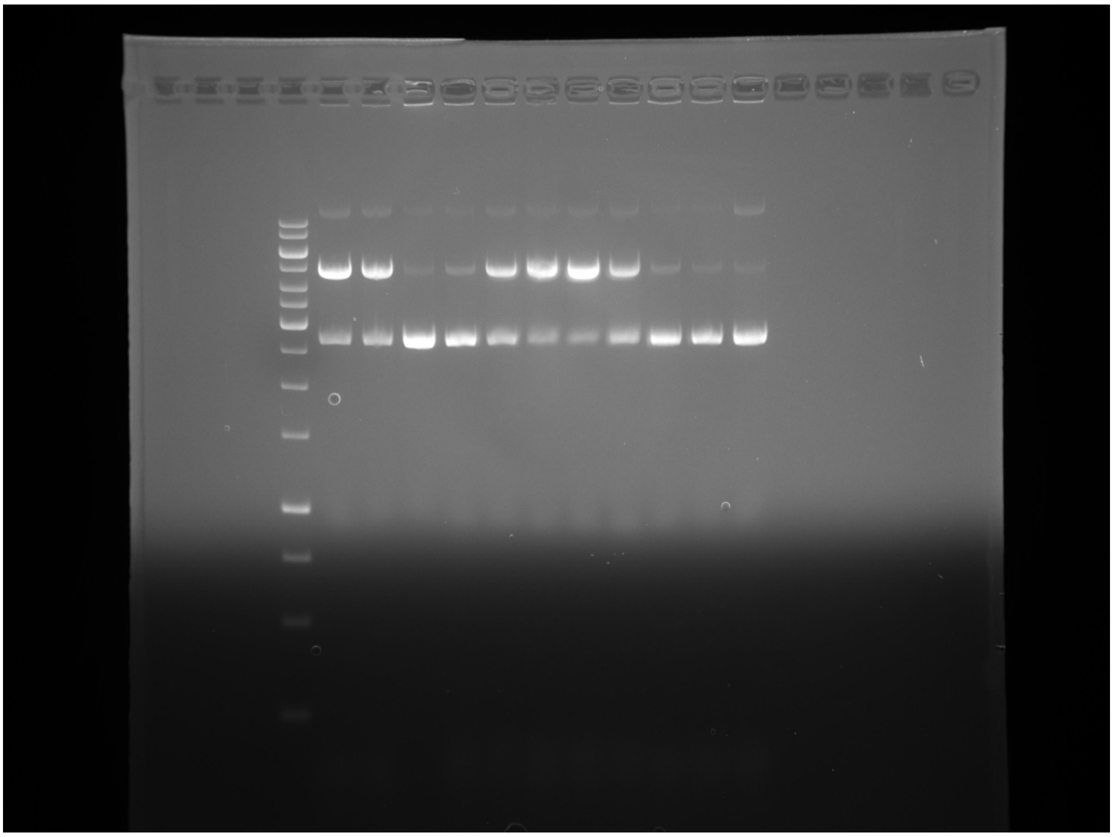

Extended Data Fig. 1e (5 mins, Rep2)

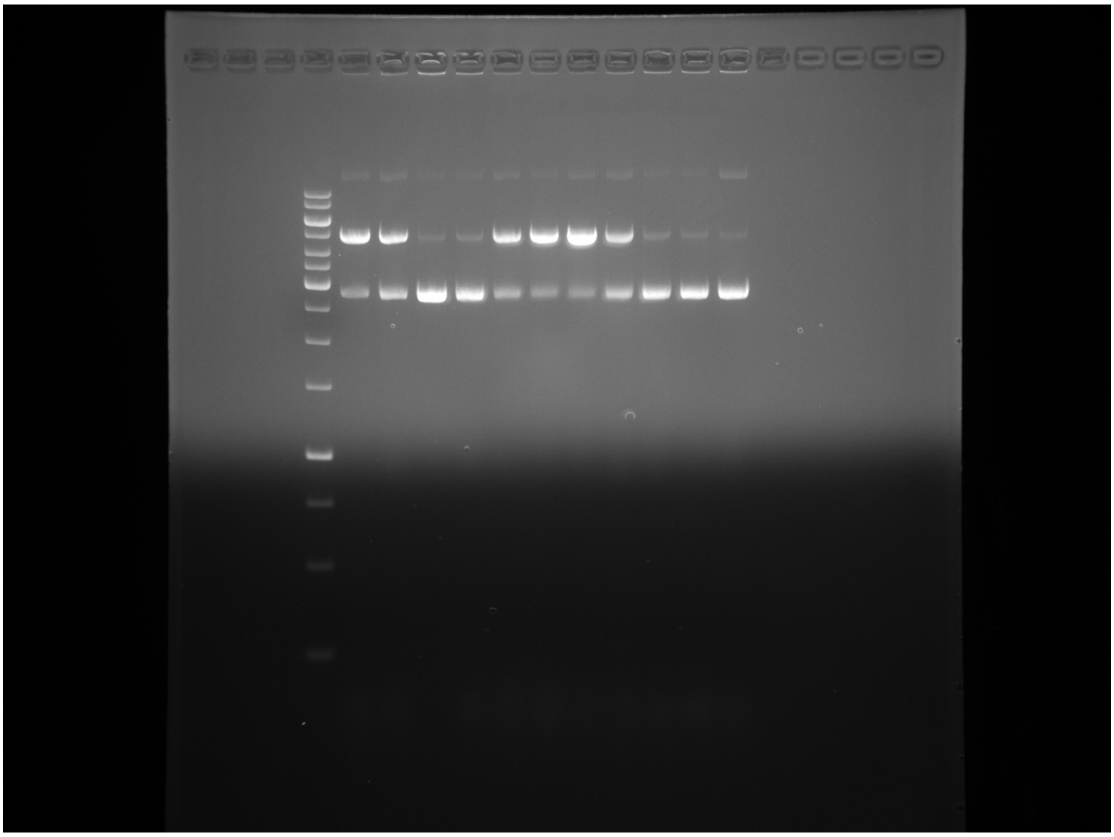

Extended Data Fig. 1e (15 mins, Rep1)

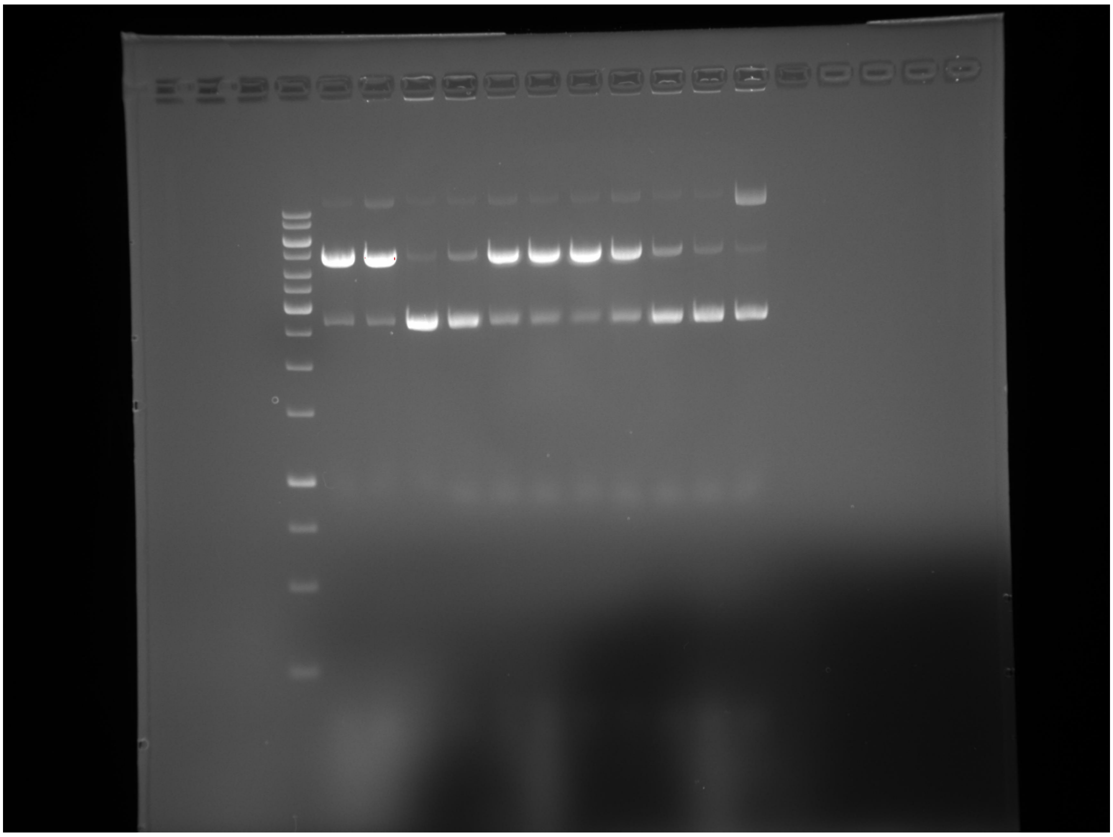

Extended Data Fig. 1e (15 mins, Rep2)

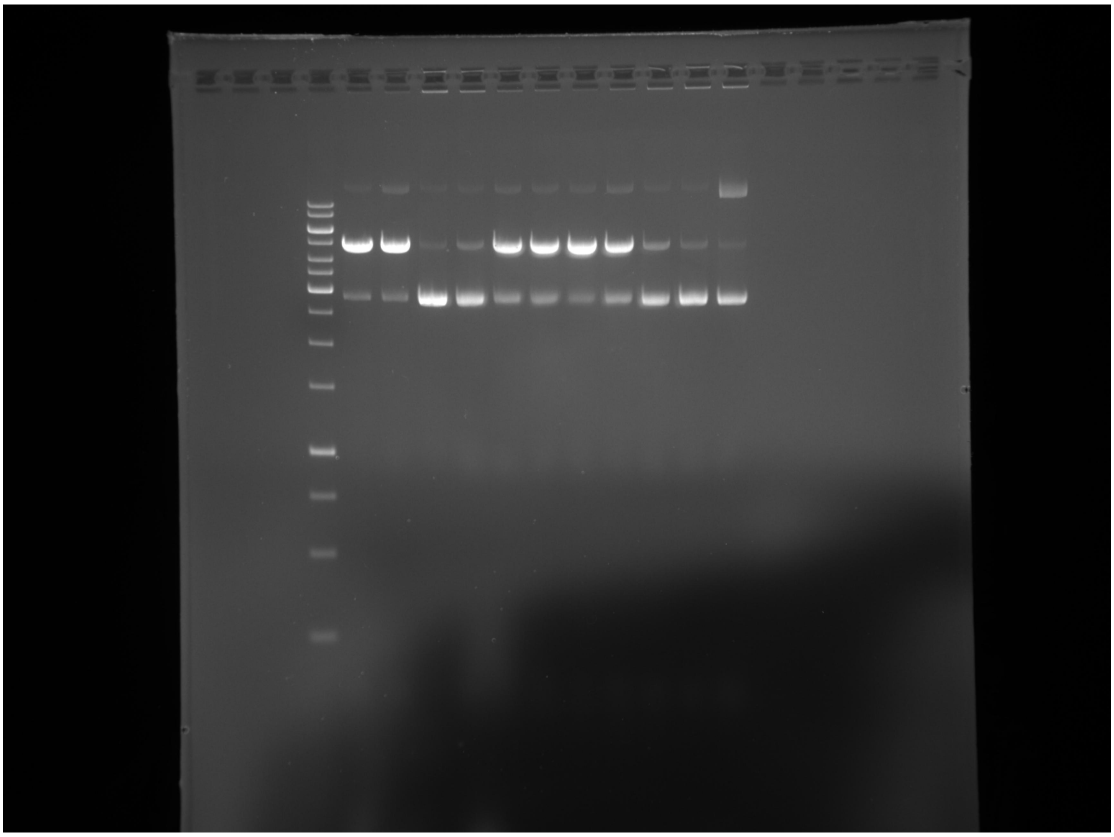

Extended Data Fig. 1e (60 mins, Rep1)

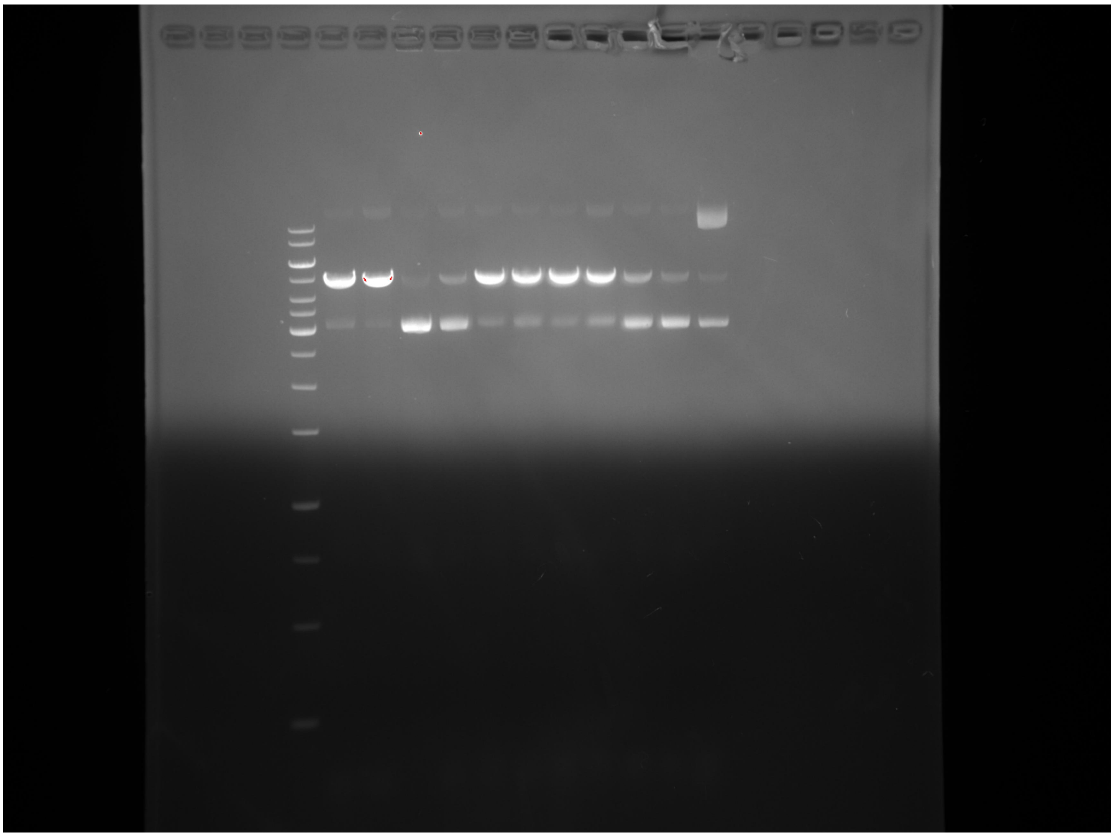

Extended Data Fig. 1e (60 mins, Rep2)

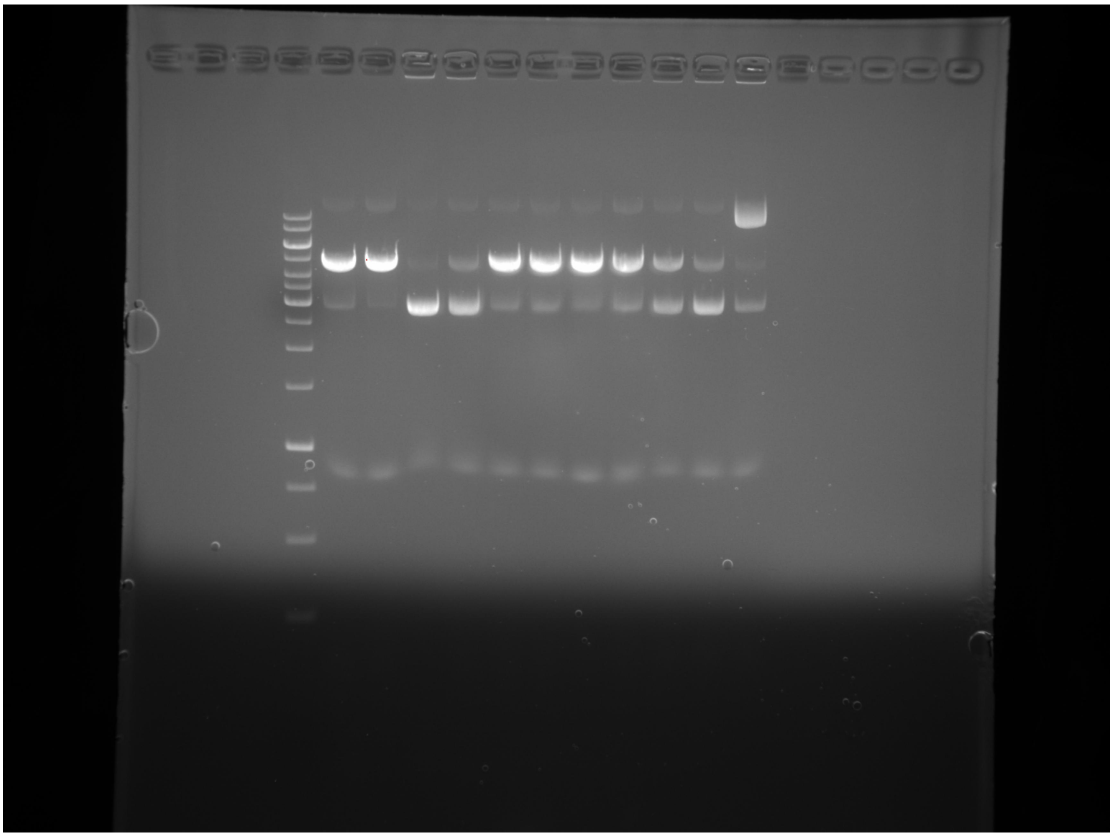

Extended Data Fig. 1e (180 mins, Rep1)

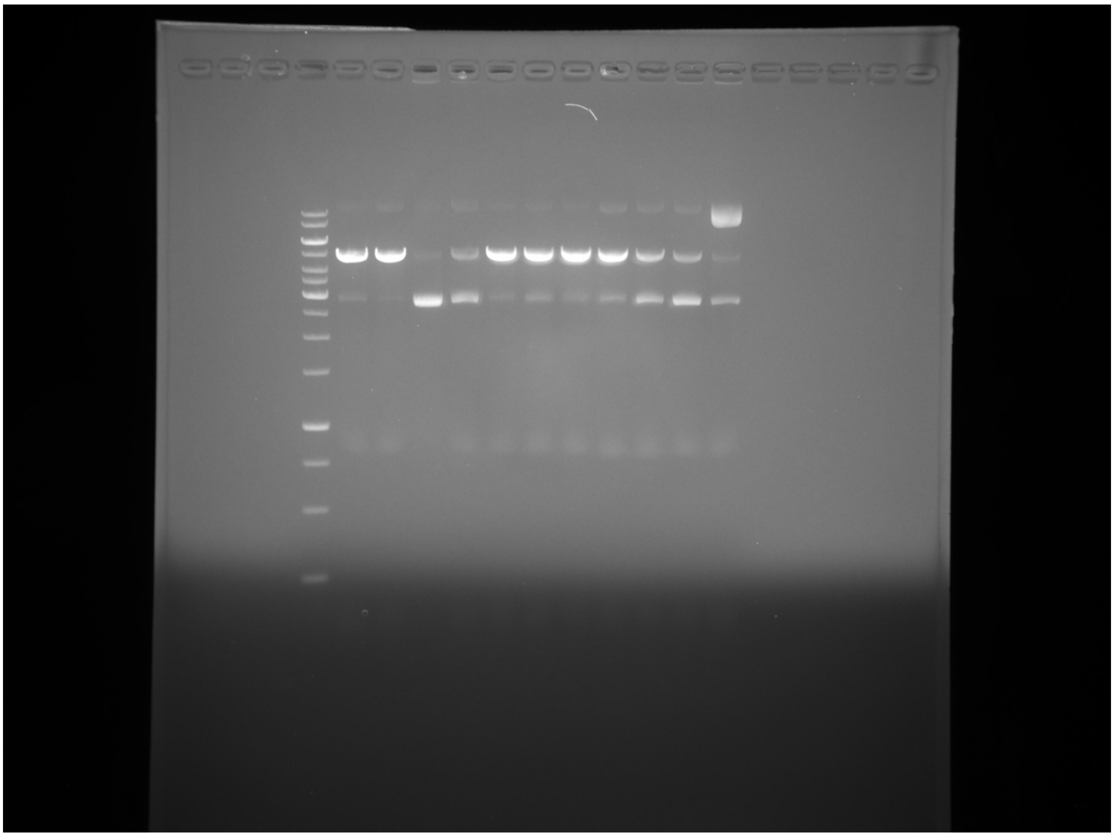

Extended Data Fig. 1e (180 mins, Rep2)

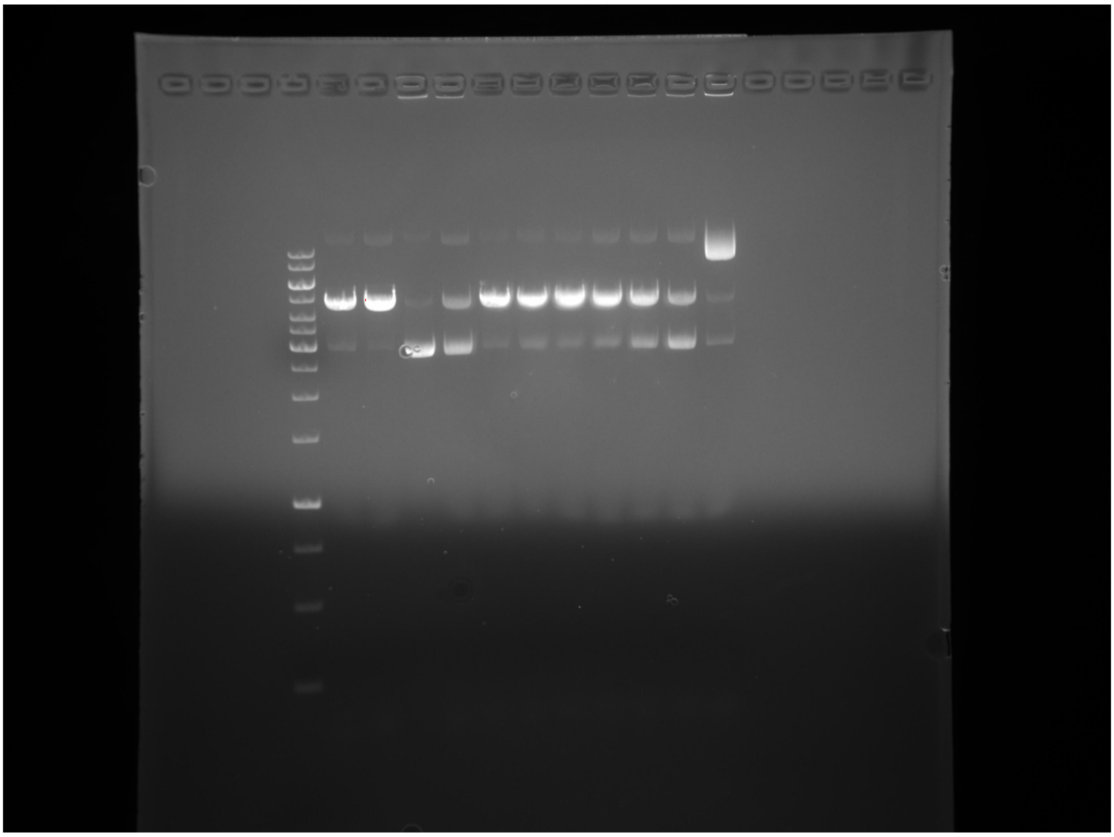

Extended Data Fig. 2a, 2c (Rep1)

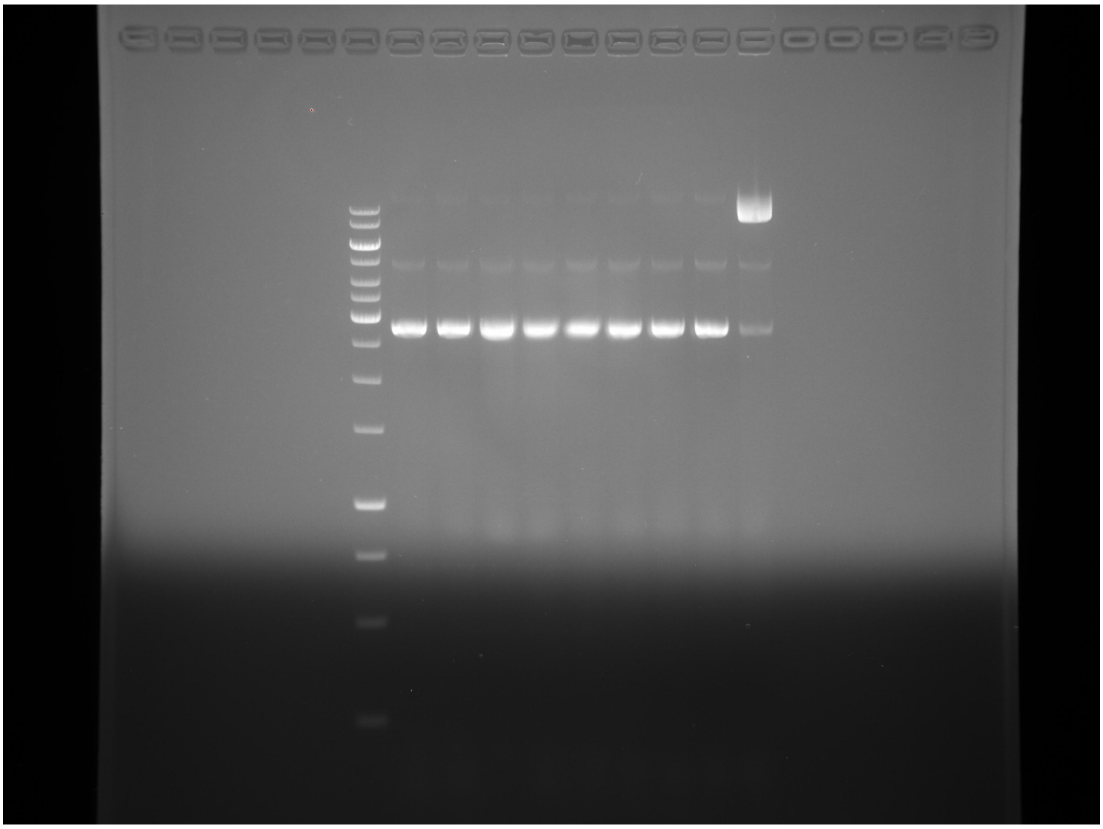

Extended Data Fig. 2a, 2c (Rep2)

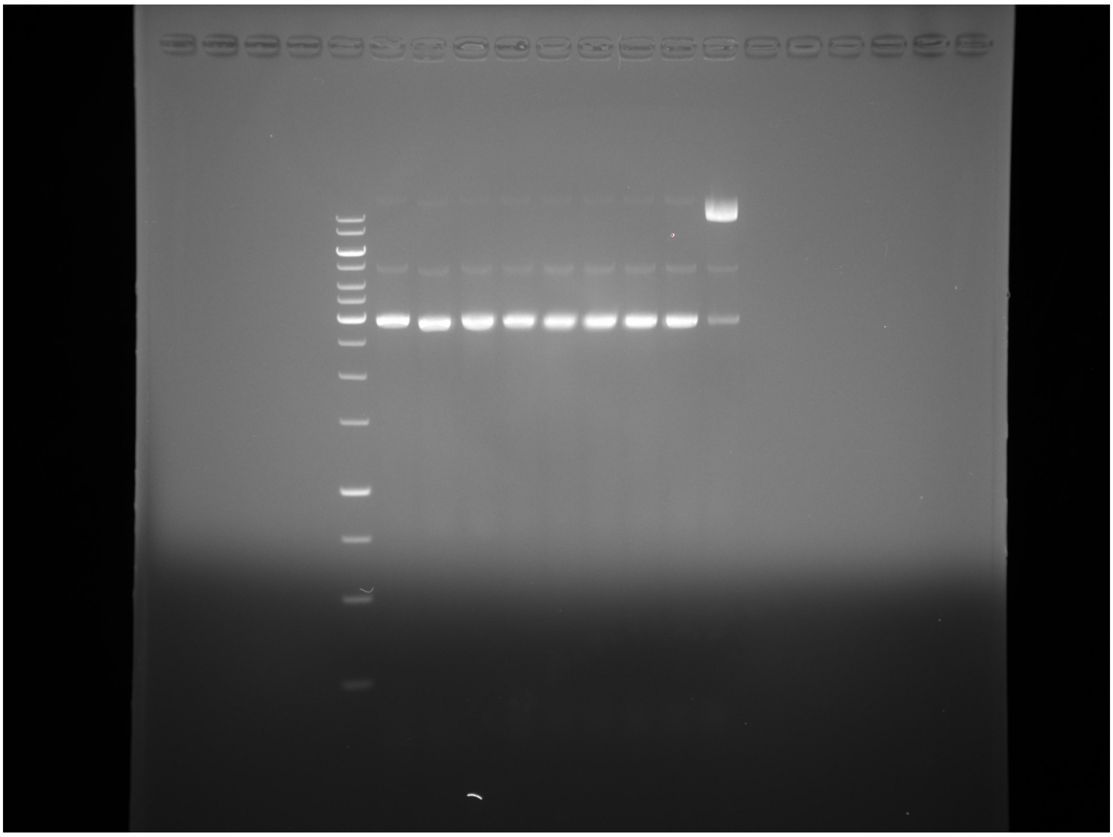

Extended Data Fig. 2a, 2c (Rep3)

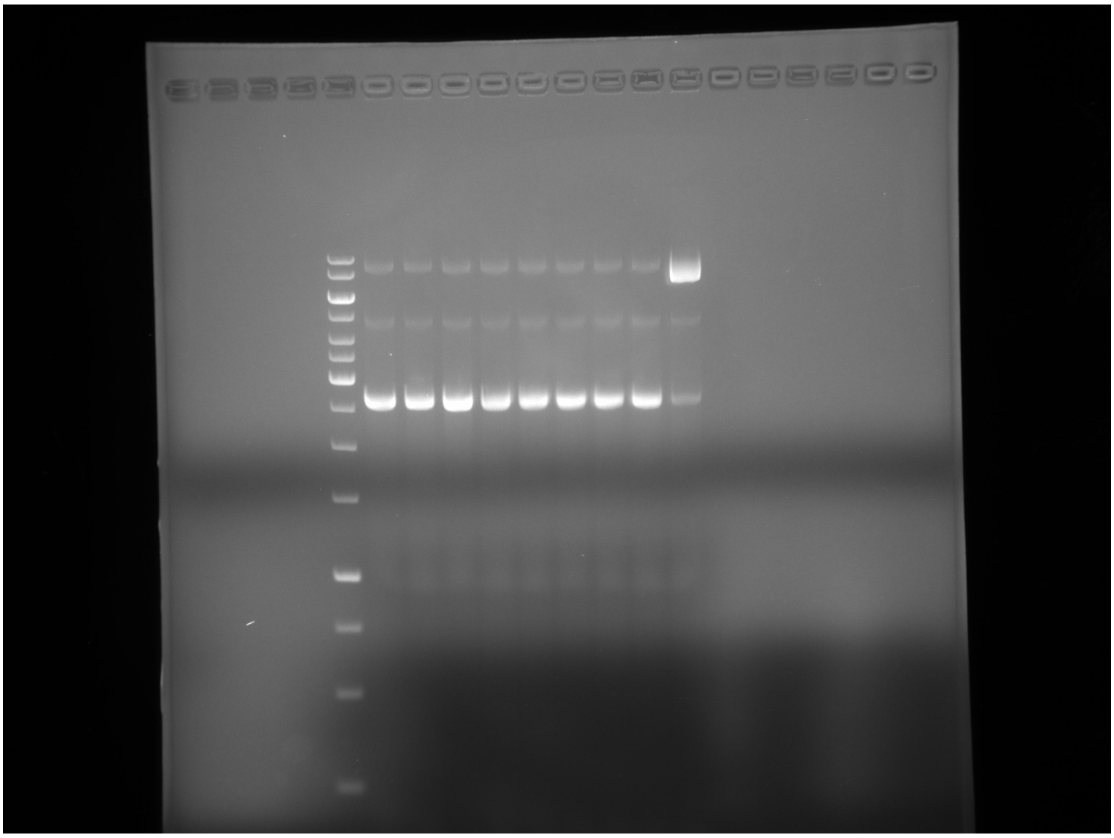

Extended Data Fig. 2b, 2c (Rep1)

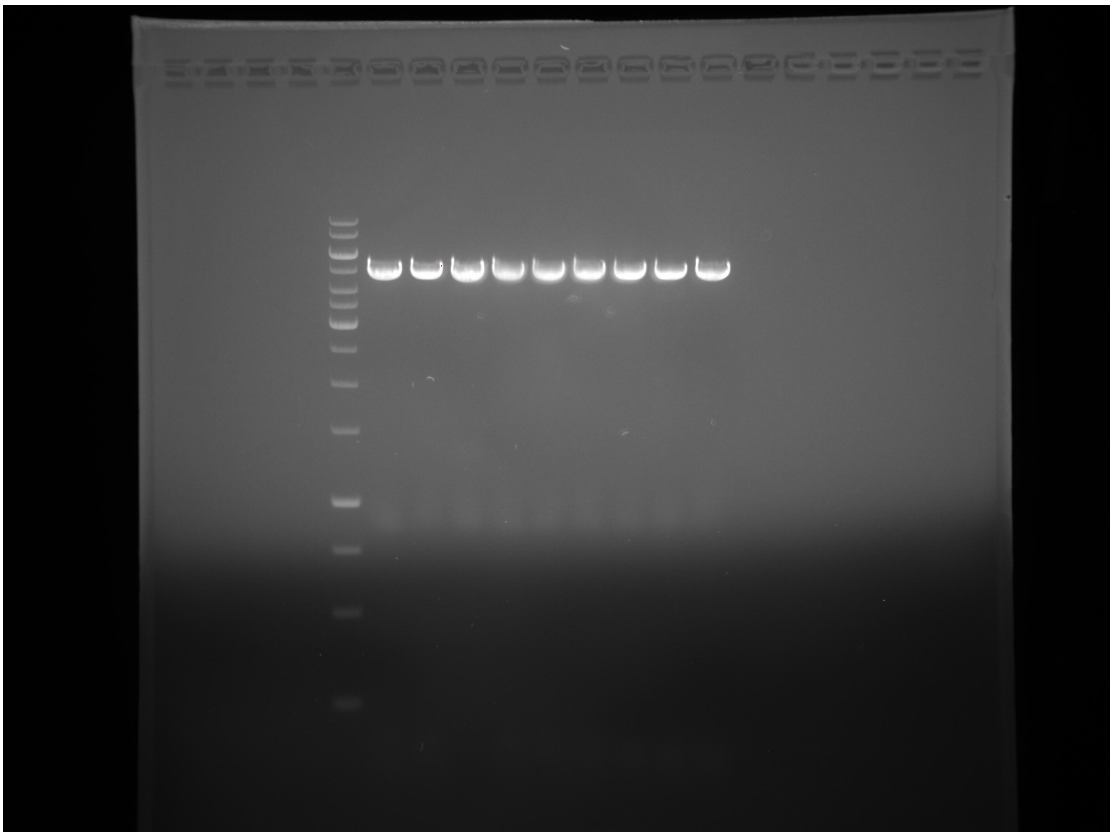

Extended Data Fig. 2b, 2c (Rep2)

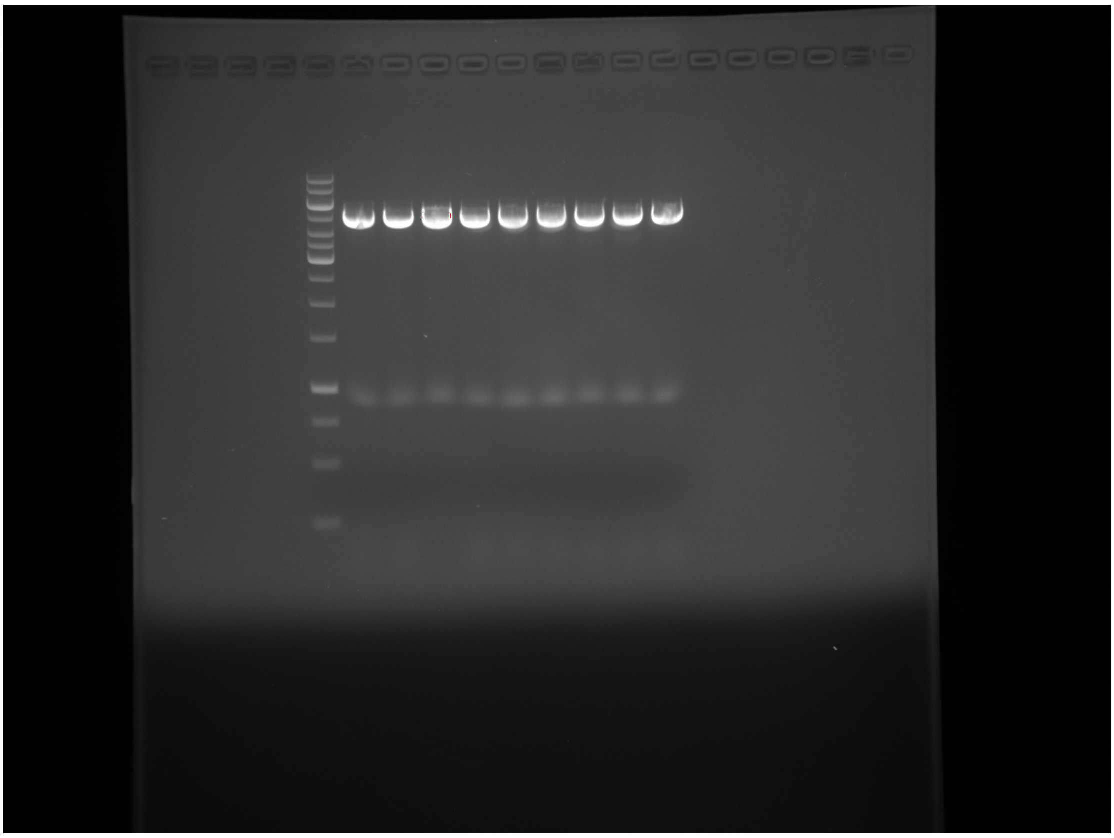

Extended Data Fig. 2b, 2c (Rep3)

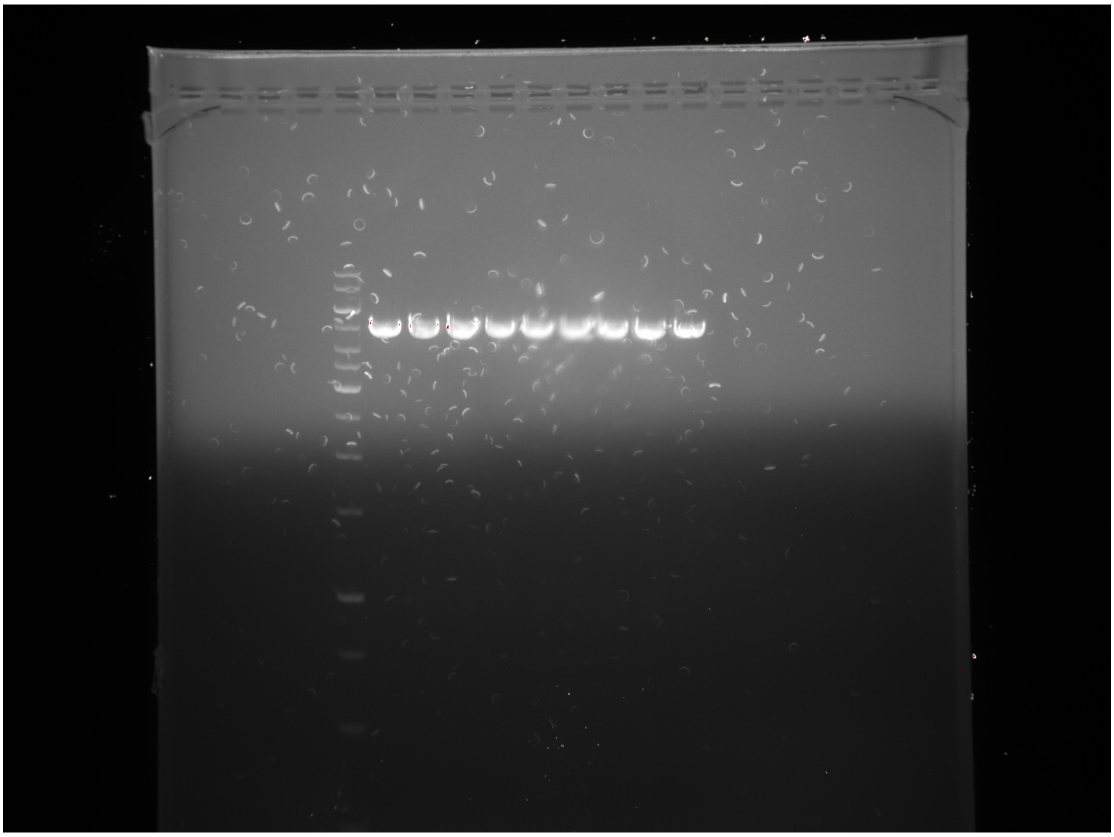

Extended Data Fig. 2d, 2e (Rep1)

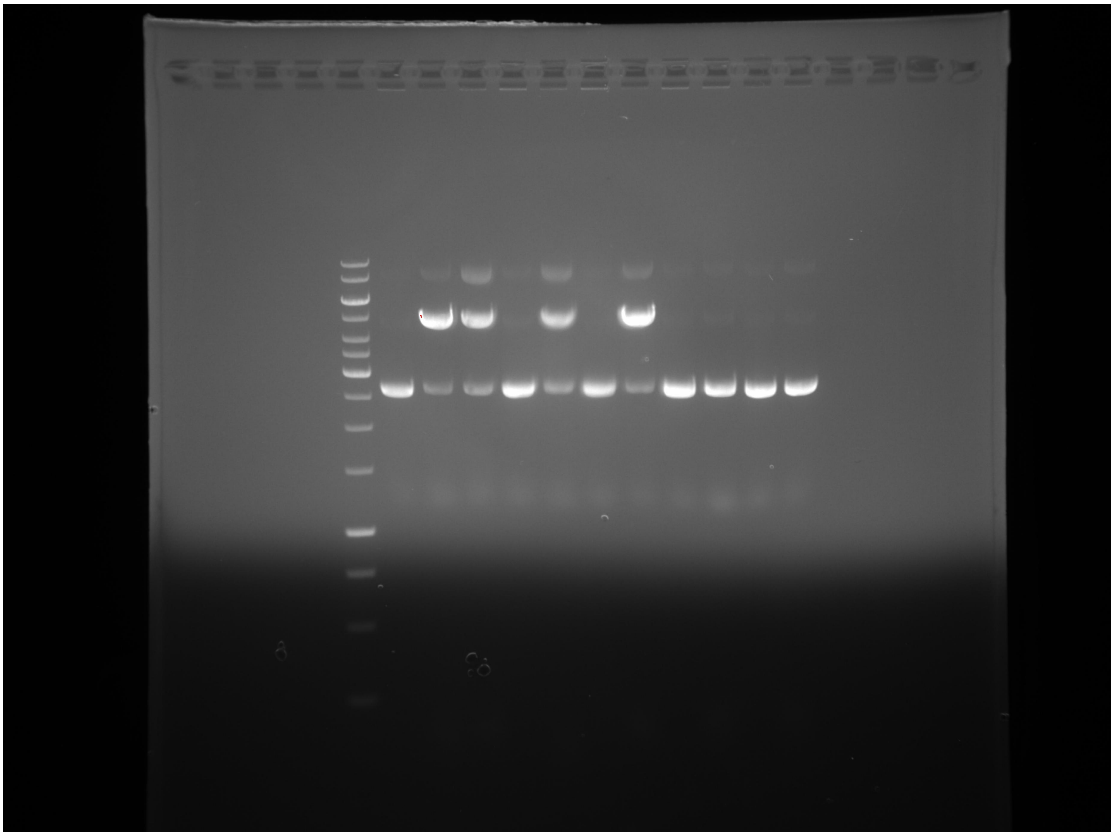

Extended Data Fig. 2d, 2e (Rep2)

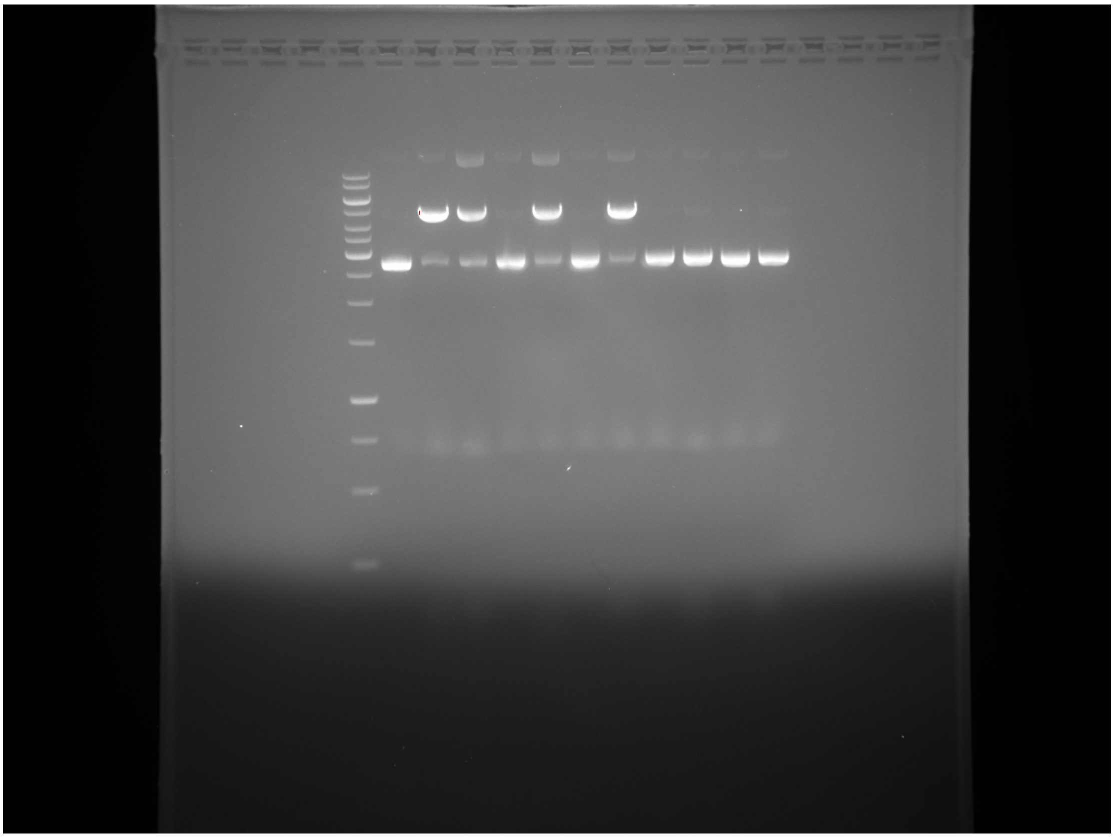

Extended Data Fig. 2d, 2e (Rep3)

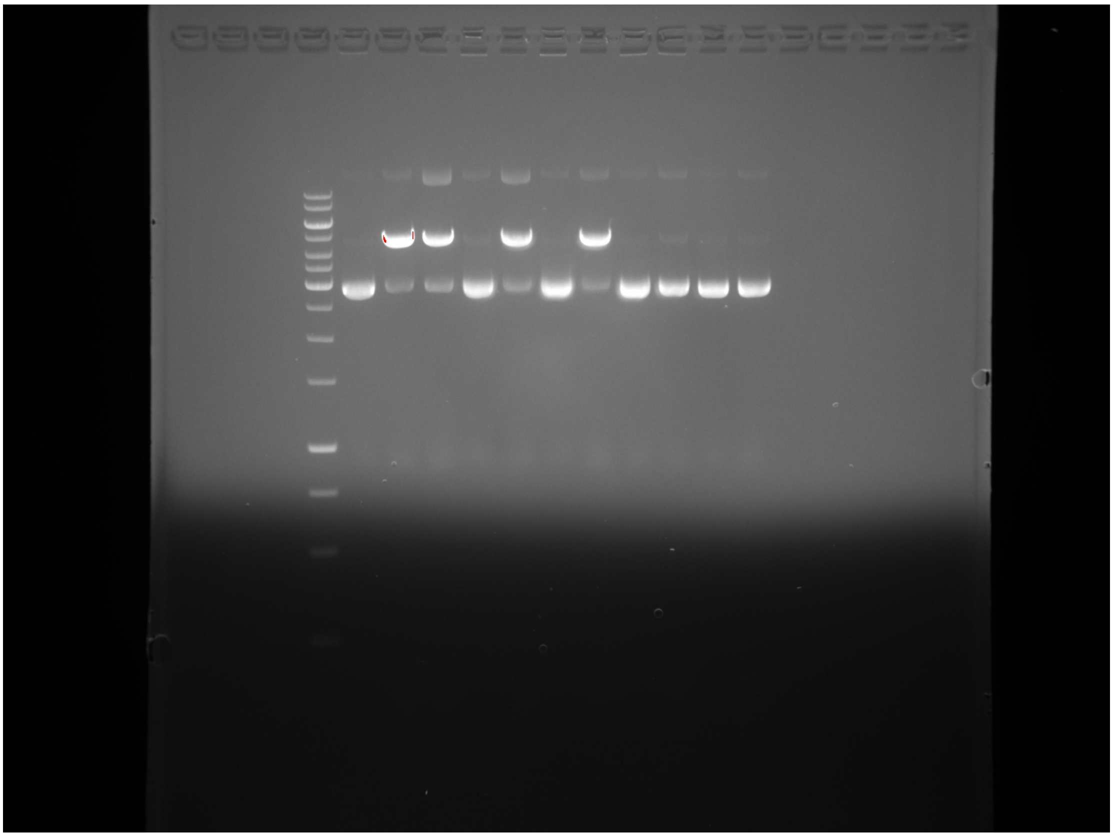

Extended Data Fig. 2f, 2g (Rep1)

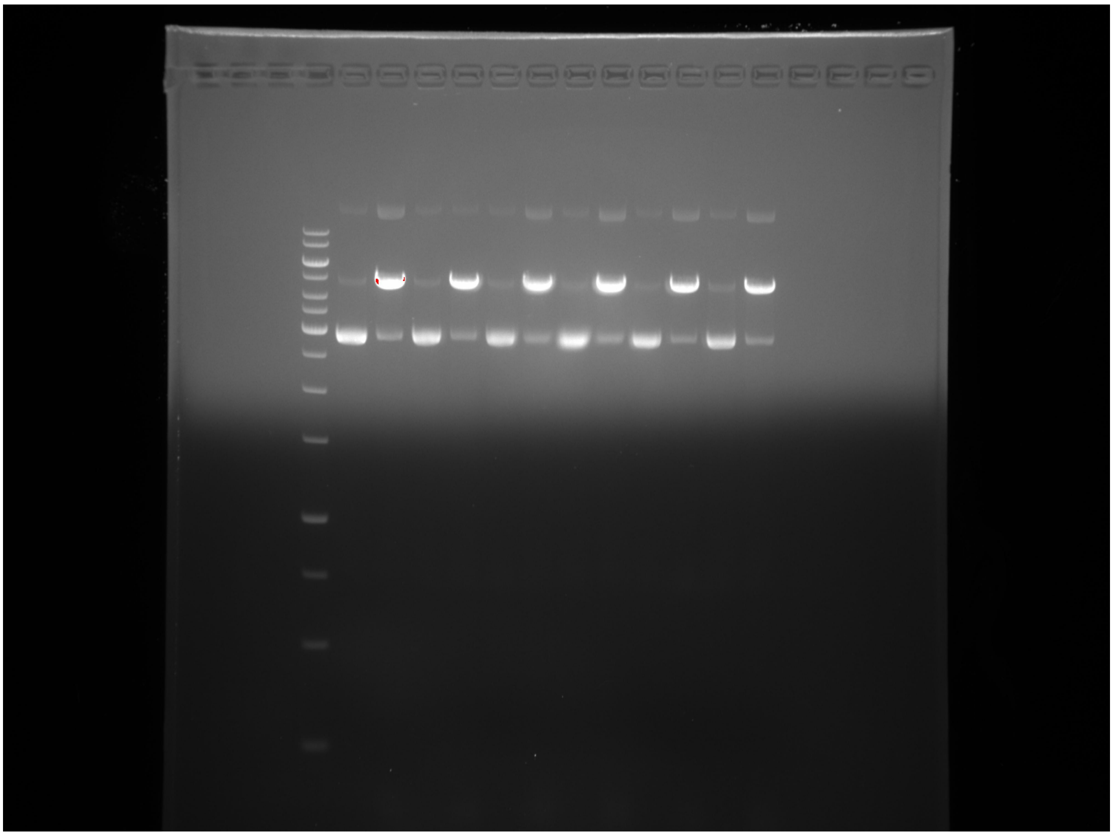

Extended Data Fig. 2f, 2g (Rep2)

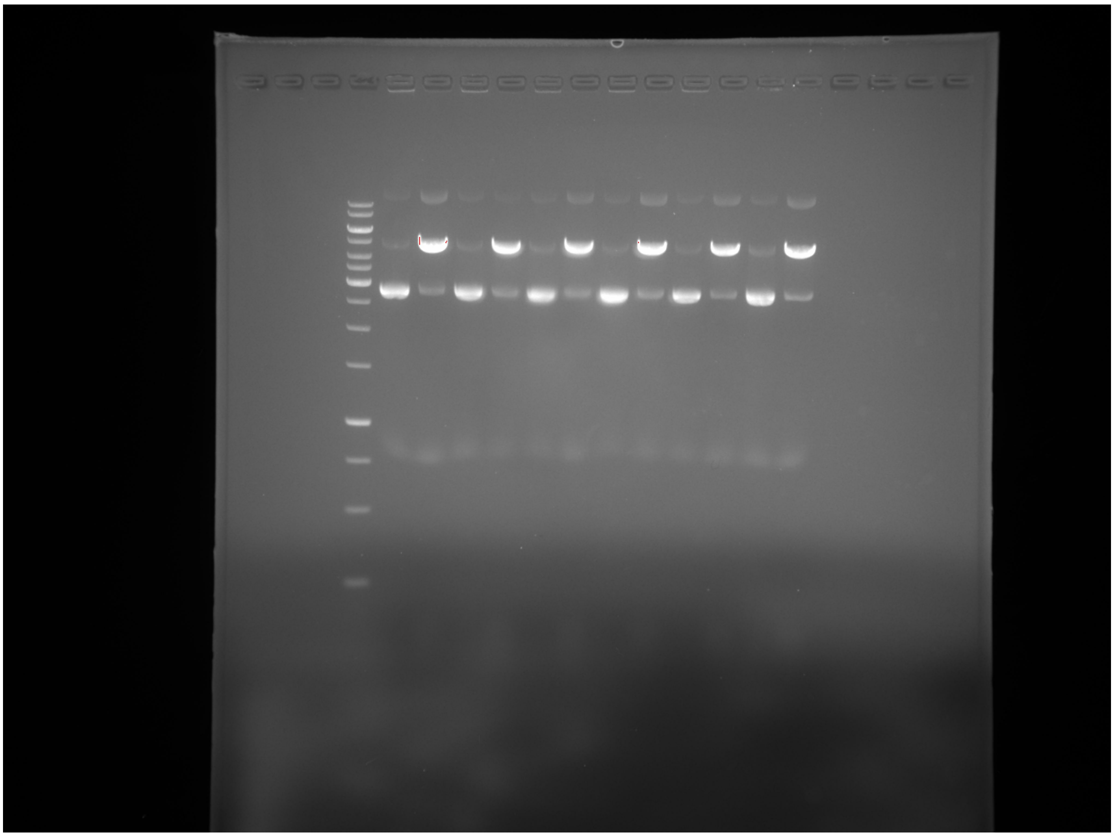

Extended Data Fig. 3a, 3c (Rep1)

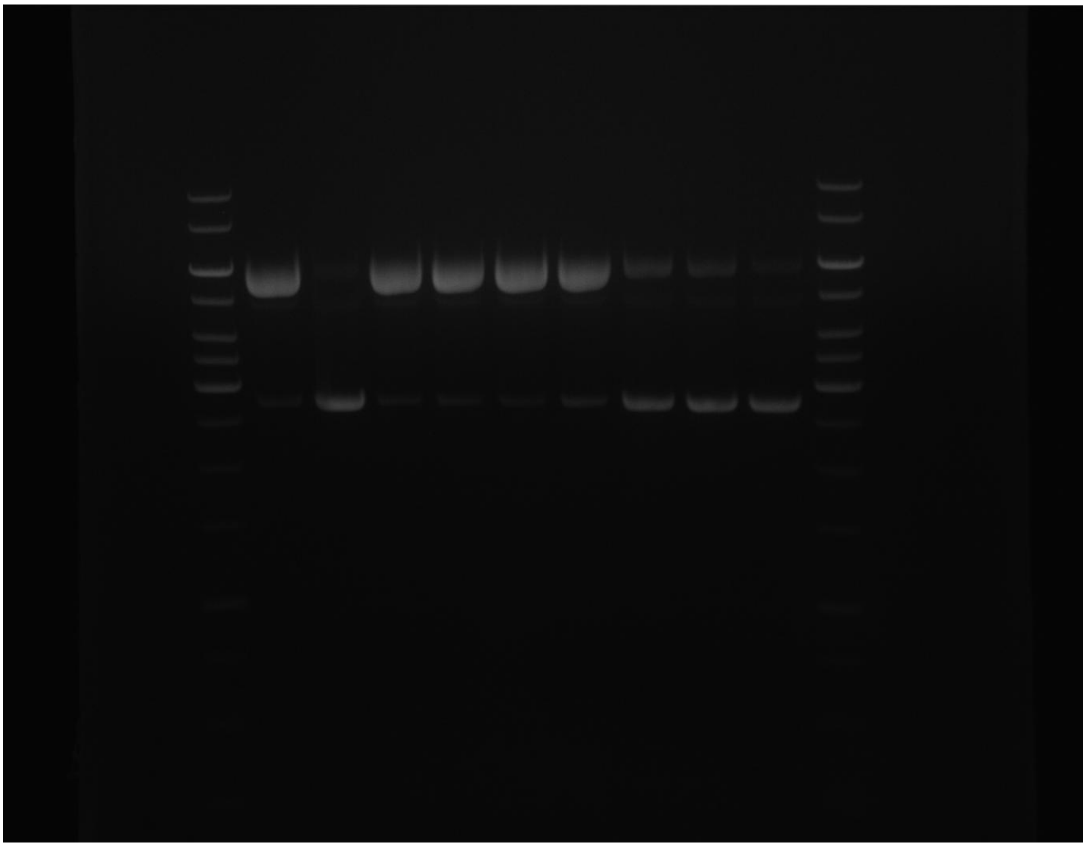

Extended Data Fig. 3a, 3c (Rep2)

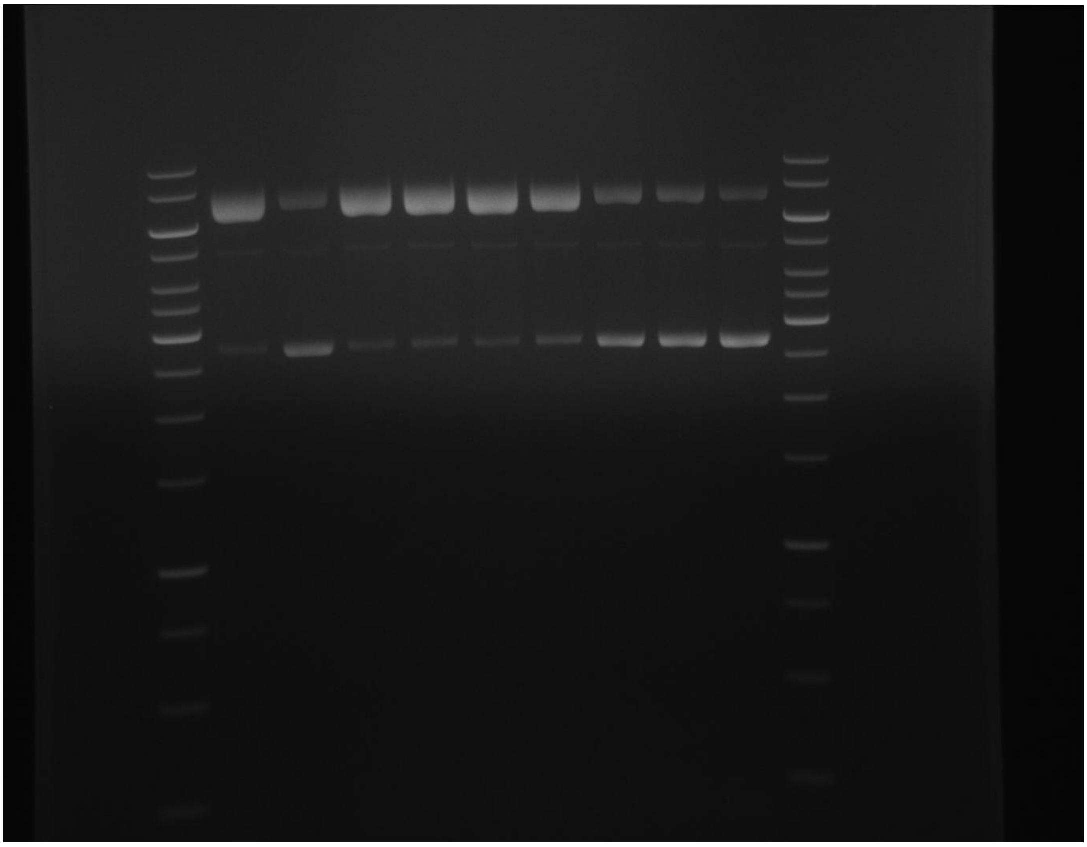

Extended Data Fig. 3a, 3c (Rep3)

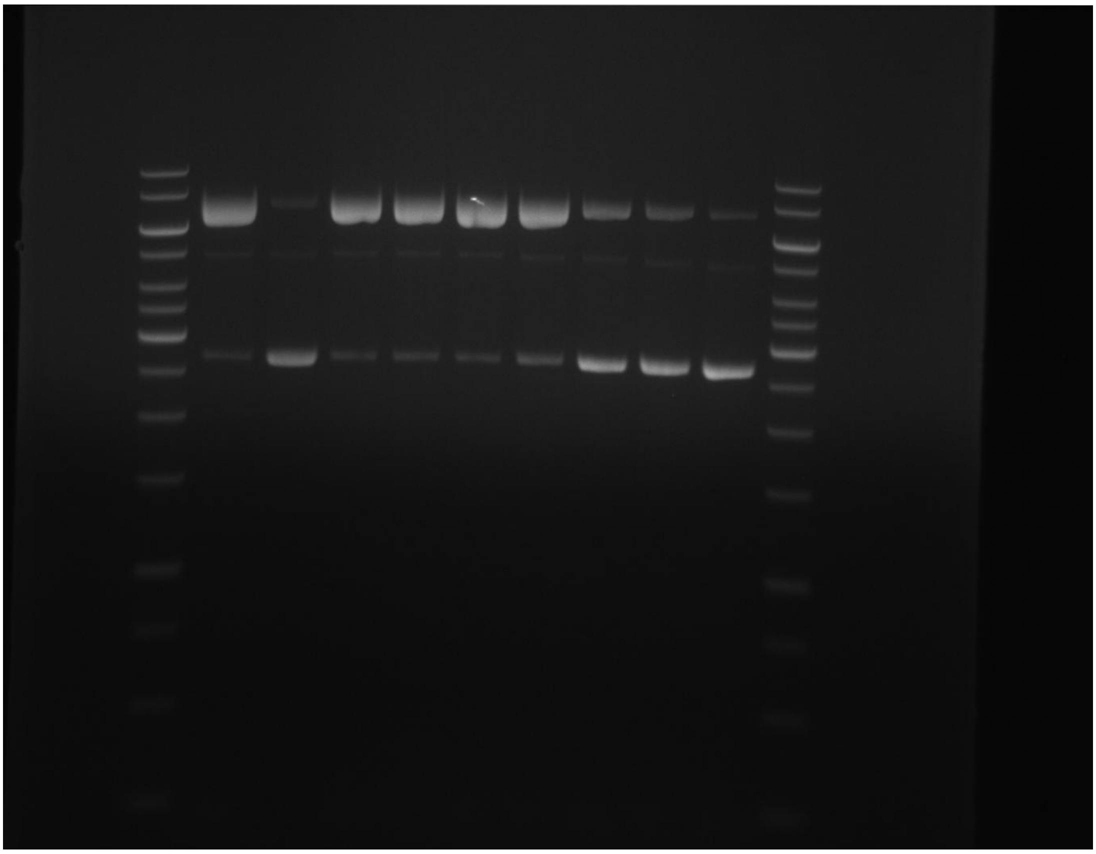

Extended Data Fig. 3b, 3c (Rep1)

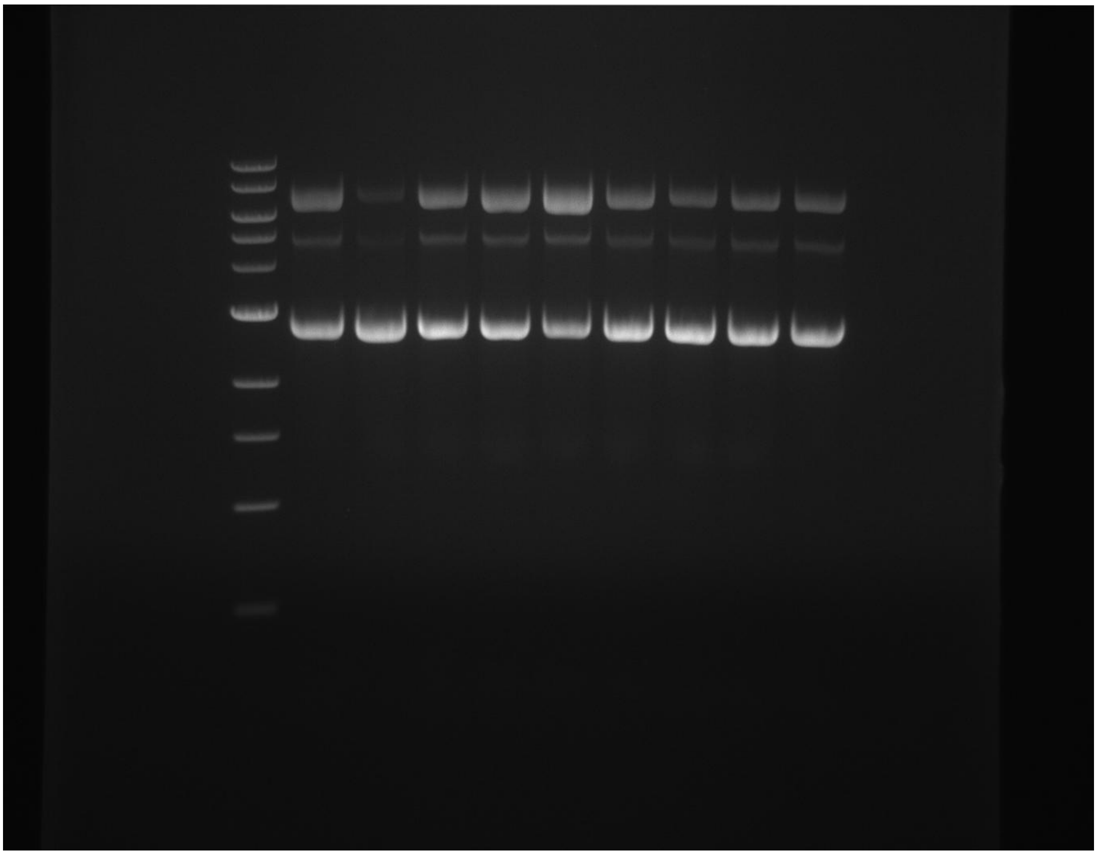

Extended Data Fig. 3b, 3c (Rep2)

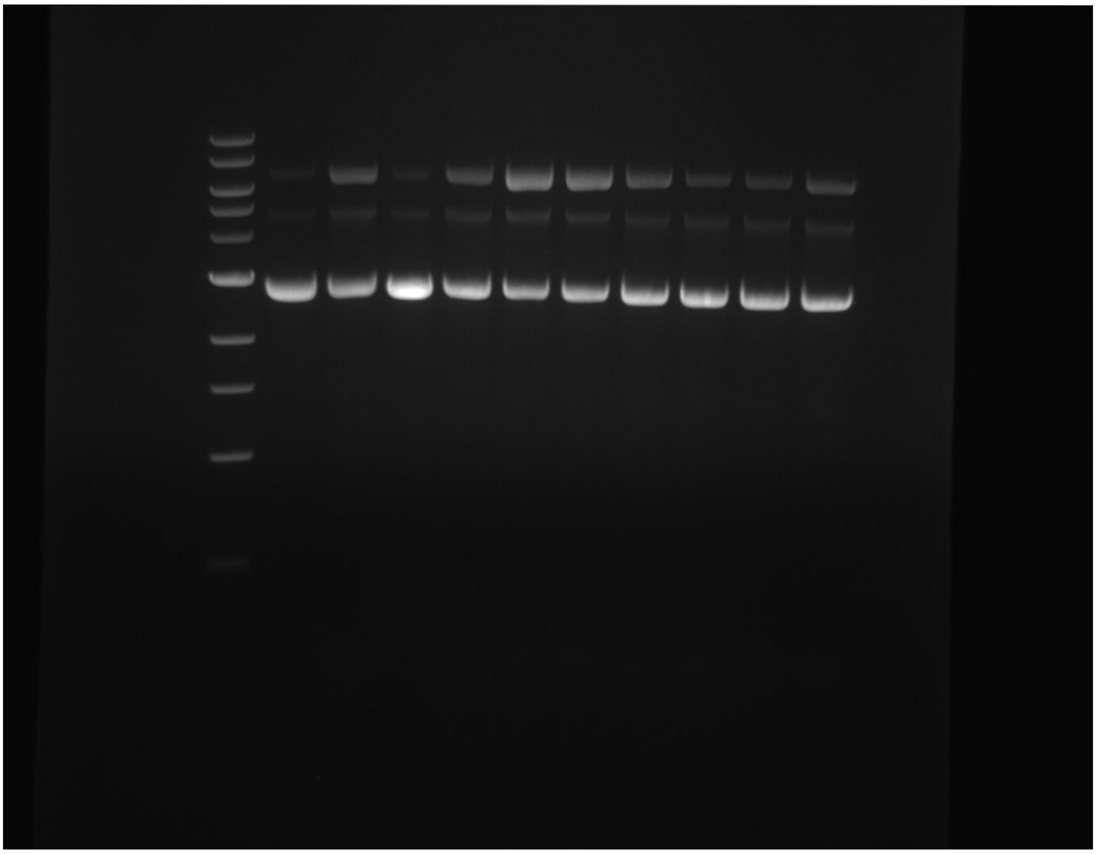

Extended Data Fig. 5b

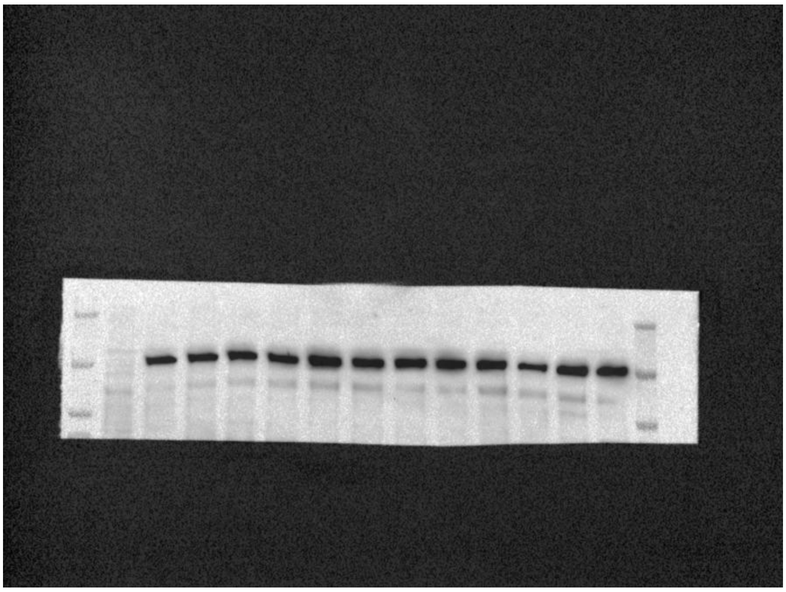

SpCas9-Flag

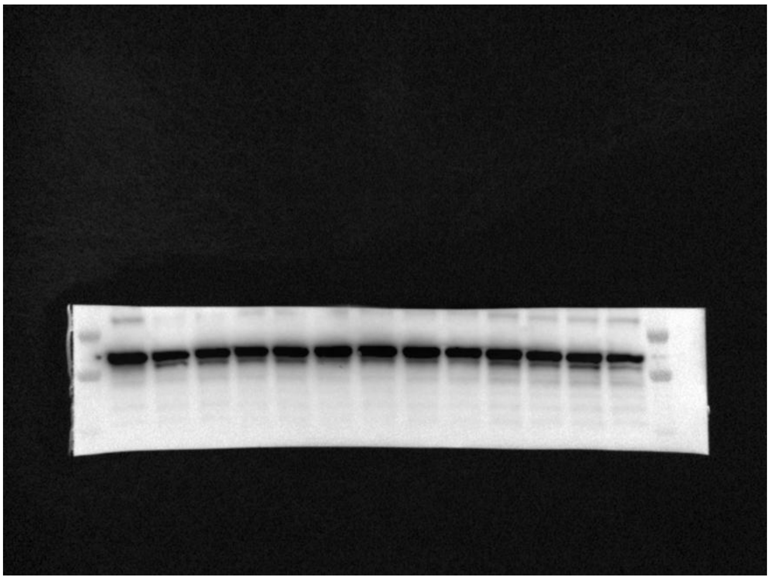

β-actin
